# PEBP4 alleviates muscle wasting in lung cancer cachexia via KEAP1–NRF2-mediated redox homeostasis

**DOI:** 10.1038/s41419-026-08925-5

**Published:** 2026-06-03

**Authors:** Yaru Xia, Yuqi Han, Weiquan Li, Tiexi Yu, Diaoyi Tan, Daojia Miao, Wen Li, Jiaming Wu, Qianqian Luo, Jiemin Li, Pian Liu, Hongli Liu, Guixiao Huang, Xiaoping Zhang, Hongmei Yang

**Affiliations:** 1 Department of Pathogenic Biology, School of Basic Medicine, Tongji Medical College, Huazhong University of Science and Technology, Wuhan, China; 2 Department of Urology, Union Hospital, Tongji Medical College, Huazhong University of Science and Technology, Wuhan, China; 3 Cancer Center, Union Hospital, Tongji Medical College, Huazhong University of Science and Technology, Wuhan, China; 4 Department of Urology, The Third Affiliated Hospital of Shenzhen University: Shenzhen Luolu Hospital Group Luolu People’s Hospital, Shenzhen, China; 5 Shenzhen Huazhong University of Science and Technology Research Institute, Shenzhen, China

**Keywords:** Cancer metabolism, Non-small-cell lung cancer

## Abstract

Cancer-associated cachexia (CAC) is a multifactorial metabolic syndrome characterized by progressive skeletal muscle wasting. However, the molecular link between tumor metabolic stress and muscle degradation remains elusive. Here, we identify phosphatidylethanolamine-binding protein 4 (PEBP4) as a key regulator of muscle homeostasis under cachectic conditions. PEBP4 expression is markedly suppressed in lung cachectic models and is inversely correlated with tumor-derived lactate levels. Mechanistically, PEBP4 stabilizes NRF2 by competitively binding to KEAP1, enhancing antioxidant defense, inhibiting NF-κB signaling, and downregulating muscle atrophy-related genes *MuRF*1 and *Fbxo32* (also known as *Atrogin-1*). In vitro and in vivo overexpression of PEBP4 mitigates oxidative stress, preserves muscle mass, and improves strength and endurance in Lewis lung carcinoma tumor-bearing mice. These protective effects are significantly attenuated by NRF2 inhibition, highlighting its critical role in PEBP4-mediated signaling. Collectively, our findings uncover a tumor lactate–PEBP4–NRF2 axis linking cancer metabolism to redox imbalance and muscle wasting, and suggest the therapeutic potential of targeting the PEBP4–NRF2 pathway in lung cancer–associated cachexia.

## Introduction

Cancer-associated cachexia (CAC) is a multifactorial metabolic syndrome characterized by progressive skeletal muscle wasting and adverse clinical outcomes. Affecting up to 80% of patients with advanced malignancies [1–3], CAC markedly compromises treatment tolerance and survival [4, 5], yet effective therapies remain unavailable [6–8].

A hallmark of CAC is skeletal muscle atrophy, primarily attributed to disrupted protein homeostasis [9]. Tumor-derived factors suppress anabolic IGF-1/PI3K/AKT/mTOR signaling while activating catabolic pathways such as the ubiquitin–proteasome system and the autophagy–lysosome pathway [10]. In addition to these well-characterized cascades, increasing evidence highlights oxidative stress as a significant contributor to CAC progression [11–13].

In chronic conditions such as sarcopenia [14–16], sepsis [17], and heart failure [18], reactive oxygen species (ROS) promote muscle loss through multiple mechanisms [14, 19]. In CAC, tumor-induced metabolic and inflammatory stress exacerbates ROS accumulation in muscle, triggering FOXO and NF-κB signaling to amplify proteolysis [11–13, 20]. However, upstream regulators of ROS overload and impaired antioxidant defense in cachectic muscle remain mostly unidentified.

Nuclear factor erythroid 2-related factor 2 (NRF2) is a master regulator of redox homeostasis, and its activity is tightly controlled by KEAP1-mediated ubiquitination [21], and GSK-3β/β-TrCP-dependent degradation [22, 23]. NRF2 protects skeletal muscle from oxidative injury and proteotoxic stress in atrophic conditions [24, 25]. While NRF2 plays a crucial role in survival and therapy resistance in cancer, its regulation in cancer-associated cachexia remains poorly understood.

Phosphatidylethanolamine-binding protein 4 (PEBP4) is a cytoplasmic scaffold protein that coordinates intracellular signaling via kinase phosphorylation and protein–protein interactions, regulating pathways such as extracellular signal–regulated kinase (ERK) and protein kinase B (AKT). Although PEBP4 has been implicated in cancer progression [26–28] and inflammatory regulation [29–31], its potential role in redox regulation and CAC remains poorly understood. Transcriptomic analyses of publicly available Gene Expression Omnibus (GEO) datasets reveal significant downregulation of PEBP4 in cachectic skeletal muscle, with enrichment in oxidative stress–related pathways, particularly those associated with NRF2 signaling.

Proteomic profiling revealed that PEBP4 interacted with KEAP1, potentially interfering with KEAP1-mediated NRF2 degradation and enhancing downstream antioxidant responses. We observed a marked suppression of PEBP4 expression in cachectic muscle, which was likely driven by tumor-derived lactate–induced histone lactylation. This downregulation might impair NRF2 activation, exacerbate ROS accumulation, and accelerate muscle proteolysis. Our findings uncover a tumor lactate–PEBP4–KEAP1–NRF2 signaling axis that links cancer metabolism reprogramming with skeletal muscle oxidative stress and atrophy. These findings suggest that restoring PEBP4 function may present a promising therapeutic strategy for preserving muscle mass in cancer patients.

## Materials and methods

### Cell culture and drug treatments

C2C12 myoblasts (SCSP-505), Lewis lung carcinoma (LLC; SCSP-5252), and HEK293T (SCSP-502) cells were obtained from the Cell Bank of the Chinese Academy of Sciences (Shanghai, China). All cells were maintained in Dulbecco’s Modified Eagle Medium (DMEM; 11965092, Gibco) supplemented with 10% fetal bovine serum (FBS; C04001-500, Biological Industries) and 1% penicillin–streptomycin (BL505A, Biosharp) at 37 °C in a humidified incubator containing 5% CO_2_.

To induce myogenic differentiation, C2C12 cells were cultured to approximately 80% confluence, after which the growth medium was replaced with DMEM supplemented with 2% horse serum (26050088, Gibco). The differentiation medium was refreshed every other day, and mature myotubes formed after 4–6 days were collected for subsequent experiments. LLC-conditioned medium (LCM) was prepared by culturing LLC cells in serum-free DMEM for 48 h after they reached approximately 90% confluence. The collected supernatant was mixed with complete DMEM (containing 10% FBS) at a 1:3 (v/v) ratio and applied to differentiated C2C12 myotubes.

Cells were treated with ML385 (20 μM), MG132 (10 μM), chloroquine (CQ; 5 μM), cycloheximide (CHX; 10 μM), GSK2837808A (10 μM), 2-deoxy-D-glucose (2-DG; 20 mM), and CCT128930 (10 nM), all purchased from MedChemExpress (USA). Hydrogen peroxide (H_2_O_2_; 100 μM) and sodium lactate (50 mM) were obtained from Sigma-Aldrich (USA). All compounds were dissolved in dimethyl sulfoxide as stock solutions, except for H_2_O_2_ and sodium lactate, which were dissolved in sterile water. Working concentrations were freshly prepared by diluting the stock solutions in cell culture medium immediately before use.

### Plasmid construction and transfection

Full-length *Mus Keap1* complementary DNA (cDNA) was cloned into the pcDNA3.1-Myc vector to generate a Myc-tagged construct. The *Pebp4* cDNA was inserted into the Ubi-MCS-3Flag vector to obtain a Flag-tagged PEBP4 protein. A series of KEAP1 truncation constructs (∆BTB, ∆IVR, ∆DGR, and the DGR domain alone) were generated by polymerase chain reaction (PCR) and subcloned into pcDNA3.1-Myc (GENE CREATE, Wuhan, China). KEAP1 point mutants (C77S, C151S, Y85F, S172A, and S104A) were produced via site-directed mutagenesis and inserted into the pcDNA3.1-Myc vector (GENEWIZ, Suzhou, China). All plasmid constructs were verified by Sanger sequencing.

Plasmids and siRNAs were transiently transfected into C2C12 or HEK293T cells using Lipofectamine™ 2000 (Thermo Fisher Scientific, 11668019) following the manufacturer’s protocol. The siRNA sequences used were as follows: si-*Saa3* (sense, 5′-GGGAGUUGACAGCCAAAGATT-3′; antisense, 5′-UCUUUGGCUGUCAACUC

CCTT-3′) and si-*Scgb1a1* (sense, 5′-CAUCUGCCCAGGAUUUCUUTT-3′; antisense, 5′-AAGAAAUCCUGGGCAGAUGTT-3′). A non-targeting siRNA was used as a negative control. All siRNAs were synthesized by JTSBIO Co., Ltd., Wuhan, China. Lentiviral particles encoding PEBP4 (GeneChem, Shanghai, China) or CRISPR/Cas9 constructs targeting KEAP1 (Focus Biology, Shanghai, China), with the sgRNA sequence 5′-GGACTTTCGTAGCCCCCATG-3′, were used to infect C2C12 cells at a multiplicity of infection (MOI) of 30, according to the manufacturers’ instructions.

### RNA isolation and quantitative real-time PCR

Total RNA was isolated from C2C12 myoblasts and skeletal muscle tissues using the MagZol Reagent (Magen, R4801), following the manufacturer’s protocol. cDNA was synthesized from 1 μg of total RNA using the HiScript II Q RT SuperMix (Vazyme, R223-01). Quantitative real-time PCR (qPCR) was performed using SYBR Green Master Mix (Vazyme, Q312-02) on a StepOnePlus™ Real-Time PCR System (Applied Biosystems). *Gapdh* served as the housekeeping gene for normalization. Relative messenger RNA (mRNA) expression levels were calculated using the 2^^−ΔΔCT^ method. The primer sequences used are provided in Supplementary Table S1.

### Protein analysis

C2C12 cells and muscle tissues were lysed in RIPA buffer (P0013C, Beyotime) containing phenylmethylsulfonyl fluoride (PMSF), and protease and phosphatase inhibitors. Protein concentrations were determined using the bicinchoninic acid assay (23227, Thermo Fisher Scientific). Equal amounts of protein (30 μg) were separated by 8–12% SDS–PAGE, transferred onto polyvinylidene difluoride membranes (03010040001, Millipore), blocked with 5% non-fat milk in PBST, and incubated overnight at 4 °C with primary antibodies, followed by horseradish peroxidase-conjugated secondary antibodies. Protein signals were visualized using enhanced chemiluminescence reagents (BL520B, Biosharp) and imaged with a ChemiDoc XRS+ system (Bio-Rad). Band intensities were normalized to internal controls and expressed as fold changes relative to the control group. Antibody information is provided in Supplementary Table S2.

For immunoprecipitation (IP) analysis, C2C12 cell pellets were lysed in RIPA buffer, centrifuged at 13,000 × *g* for 10 min at 4 °C, and incubated overnight at 4 °C with antibodies against PEBP4, NRF2, KEAP1, Flag, Myc, or immunoglobulin G (IgG). Immune complexes were captured using Protein A/G magnetic beads (pre-blocked with 0.5% PBST), washed, boiled in loading buffer, and analyzed by immunoblotting.

Nuclear and cytoplasmic fractions were isolated from transfected C2C12 cells using a commercial nuclear and cytoplasmic extraction kit (P0027, Beyotime, China) supplemented with PMSF. The isolated fractions were immediately subjected to Western blot analysis.

### Cell viability and oxidative stress assays

C2C12 cells were seeded in 96-well plates (1 × 10^4^ cells/well) and treated with serial dilutions of ML385, H_2_O_2_, or sodium lactate for 48 h or 72 h. After treatment, 110 μL of the Cell Counting Kit-8 (diluted 1:10 in fresh DMEM; A311-01, Vazyme) was added and incubated at 37 °C for 1 h. Absorbance was then measured at 450 nm using a spectrophotometer (Multiskan FC, Thermo Fisher Scientific).

Intracellular ROS levels were detected using dihydroethidium (DHE; 20 μM, KGA7502-5, KeyGEN BioTECH). Cells were incubated in serum-free medium containing DHE for 1 h at 37 °C, then collected, washed, and analyzed by flow cytometry (ID7000, Sony, Japan; 10,000 events/sample). ROS fluorescence intensity was quantified using FlowJo software (BD Biosciences).

Glutathione (GSH) levels in cells and tissues were determined using a commercial detection kit (A061-1-2, Jiancheng Bioengineering Institute, China) according to the manufacturer’s instructions. Absorbance was measured using a microplate reader, and concentrations were calculated from standard curves.

### Histochemistry and CSA analyses

Tissues were fixed in 4% paraformaldehyde, embedded in paraffin, sectioned at 4 μm, deparaffinized, and stained with hematoxylin for 3 min and eosin for 2 min. For ROS detection, 10 μm frozen sections were stained with DHE (BaiQianDu Biotechnology, Wuhan, China). Images were captured using an Olympus VS120 slide scanner. The cross-sectional area (CSA) of muscle fibers was quantified in ImageJ by measuring at least 100 fibers per sample. Investigators were blinded to group allocation during image analysis.

### Immunofluorescence and co-localization analysis

C2C12 myoblasts and myotubes were seeded in six-well plates (for myosin heavy chain [MHC] staining) or confocal dishes (for co-localization analysis). After incubation, the cells were fixed with 4% paraformaldehyde for 30 min at room temperature and washed with phosphate-buffered saline (PBS). For MHC labeling, the cells were permeabilized and blocked with 5% bovine serum albumin and 0.1% Triton X-100 for 1 h. For co-localization experiments, blocking was performed with 10% donkey serum and 0.2% Triton X-100. Primary antibodies (listed in Supplementary Table S2) were applied overnight at 4 °C, followed by incubation with fluorophore-conjugated secondary antibodies (1:200) for 2 h in the dark. Nuclei were counterstained with 4′,6-diamidino-2-phenylindole (DAPI) for 10 min. Images were captured using a Leica DMi8 microscope (for MHC staining) or a Nikon AXE confocal microscope (for co-localization analysis).

### Chromatin immunoprecipitation (ChIP) assay

ChIP–qPCR was performed in C2C12 myotubes under the indicated treatment conditions using a commercial ChIP assay kit (P2078, Beyotime) according to the manufacturer’s instructions. Chromatin was immunoprecipitated with an anti-H3K18la antibody, with normal IgG used as a negative control. Detailed antibody information is provided in Supplementary Table S2. Primers targeting the −2 kb upstream promoter region of the mouse *Pebp4* gene were designed based on H3K18la-enriched peaks (−1700 to −1400 and −1100 to −900) identified from publicly available CUT&Tag data for primary mouse myotubes (GSE195856) [32]. DNA enrichment was quantified by qPCR and normalized to input DNA. Primer sequences are provided in Supplementary Table S1.

### Animal studies and induction of cancer-associated cachexia

Seven-week-old male C57BL/6J mice were purchased from Hubei BIONT (Wuhan, China) and maintained under controlled environmental conditions (21 ± 1 °C, 55 ± 10% humidity, 12-h light/dark cycle) with ad libitum access to food and water. All experimental procedures were approved by the Animal Ethics Committee of Huazhong University of Science and Technology (IACUC No.4643). Mice were randomly assigned to experimental groups using random selection. Each group consisted of 6–8 mice, as specified in the corresponding figure legends.

To establish a lung cancer–associated cachexia model, mice were subcutaneously injected in the dorsal region with 100 μL of LLC cells (5 × 10⁷ cells/mL), while control mice received PBS, as previously described [33]. Body weight, food intake, and tumor volume were measured every 2–3 days throughout the experimental period.

Recombinant adeno-associated virus serotype 9 (AAV9) vectors (AAV9-vector or AAV9-PEBP4; 2 × 10¹¹ viral genomes in 50 μL PBS; Weizhen Biotechnology, Jinan, China) were injected intramuscularly at three sites in the gastrocnemius (GA) muscle. LLC tumor implantation was performed, followed by intramuscular AAV9-PEBP4 injection two days later, as previously described [34].

For NRF2 inhibition studies, ML385 (30 mg/kg) was administered intraperitoneally once daily for seven consecutive days before euthanasia.

Experimental endpoints were defined according to widely accepted murine cachexia criteria, which were characterized by ≥10% loss of body weight (excluding tumor mass), skeletal muscle wasting, and functional decline. Tumor growth was monitored closely to ensure that humane endpoint limits, including tumor size and clinical signs of distress, were not exceeded [35, 36]. No animals were excluded from analysis unless humane endpoints were reached.

At the time of euthanasia, the heart, inguinal white adipose tissue (iWAT), epididymal white adipose tissue (eWAT), GA, and tibialis anterior (TA) muscles were collected for histological and molecular analyses.

### Grip strength and rotarod performance tests

Grip strength was evaluated one day before euthanasia using a YLS-13A grip strength meter (Yiran Biotechnology, China). Each mouse completed eight trials for both forelimb and total limb force, and the average of the top six values was calculated. Hindlimb strength was determined by subtracting forelimb force from total limb force. All measurements were normalized to body weight and expressed as gram-force per gram (g/g).

Motor coordination and endurance were assessed using an accelerating rotarod apparatus (ZH-YLS-4C, Zhenghua, China). Mice were pre-trained for three consecutive days with three sessions per day (1.5 min per session at a constant speed of 15 rpm) to acclimate them to the apparatus. During the testing phase, the rotation speed increased linearly from 4 to 40 rpm over 5 min. The latency to fall was recorded as an indicator of motor performance. Each mouse underwent three trials per day, with inter-trial intervals of at least 30 minutes to minimize fatigue. The average latency across valid trials was calculated for statistical analysis. Data collection was performed in a blinded manner.

### Shotgun proteomics and molecular docking

PEBP4-interacting proteins were identified in C2C12 cells transfected with either a Flag-tagged PEBP4 construct or an empty Flag vector as the control. Total cellular proteins were extracted, digested with trypsin, and analyzed using liquid chromatography–tandem mass spectrometry. All experimental procedures were performed by GeneChem Technology Co., Ltd. (Shanghai, China) in accordance with standard protocols. Protein identification and quantification were conducted through database searches and validated using established bioinformatics pipelines. To ensure interaction specificity, only proteins uniquely detected in the Flag–PEBP4 group and absent in the control group were retained for further analysis.

To predict the structural interactions between PEBP4 and its potential binding partners—KEAP1, PSMA3, and AKT2—molecular docking analyses were conducted. The crystal structures of these proteins were obtained from the Protein Data Bank and preprocessed using PyMOL by removing heteroatoms and water molecules. The receptor and ligand proteins were then uploaded to the ZDOCK server for rigid-body docking. The top-ranked docking models were subsequently analyzed using the EMBL–EBI protein–protein interaction tool. A Gibbs free energy (ΔG) value below 0 indicated a thermodynamically favorable interaction, with lower ΔG values corresponding to stronger binding affinities.

### Bioinformatics analysis

RNA sequencing (RNA-seq) datasets from murine cancer cachexia models were retrieved from the GEO database (GSE132120 [37], GSE114820 [38], GSE173250 [39], and GSE142455 [40]). Differentially expressed genes (DEGs) were identified using the R packages DESeq2, edgeR, and limma (R version 4.2.2 (R Core Team, 2022)). DEGs were defined according to the following criteria: adjusted *p*-value < 0.05 and |log_2_ fold change | > 1.5. For downstream enrichment analyses, DEGs from the GSE114820 [38] dataset, which includes 31 samples, was selected as a representative cachexia-associated gene because of its larger sample size. Functional enrichment analyses were performed using Gene Set Enrichment Analysis (GSEA), Gene Ontology (GO), and Kyoto Encyclopedia of Genes and Genomes (KEGG) pathway analysis.

### Clinical data analysis

Serum lactate dehydrogenase (LDH) activity was measured in 62 patients with pulmonary nodules (non-cachectic) and 62 patients with lung adenocarcinoma (LUAD) and cachexia (body mass index < 20 kg/m^2^), according to clinical biochemistry reports. Because routine biochemical testing did not include lactate measurements, LDH activity was used as an indirect indicator of circulating lactate levels. Ethical approval for this study was granted by the Medical Ethics Committee of Tongji Medical College, Huazhong University of Science and Technology (approval number: IEC No. A302). Written informed consent was obtained from all study participants. Patient characteristics are summarized in Supplementary Tables S3 and S4.

### Lactate assessment in serum, muscle, and culture supernatants

Lactate concentrations were determined using a commercial L-lactate assay kit (S0208S, Beyotime) following the manufacturer’s instructions. The analyzed samples included serum from Lewis lung carcinoma tumor-bearing (LLC-TB) mice, homogenized GA and TA muscles, culture supernatants from LLC cells, and lysates of differentiated myotubes.

### Statistical analysis

Unless otherwise specified, all experiments were independently performed in biological triplicates. Data are presented as the mean ± standard error of the mean (SEM). Statistical analyses were conducted using GraphPad Prism, RStudio, and ImageJ software. For comparisons between two groups, unpaired two-tailed Student’s t-tests were used. For comparisons among more than two groups, one-way analysis of variance followed by appropriate post hoc tests was applied. Quantitative image analyses—such as band densitometry, ROS signal quantification, myotube diameter measurement, and CSA of muscle fibers—were conducted using ImageJ. A *p*-value < 0.05 was considered statistically significant, following the annotation scheme: *p < 0.05; **p < 0.01; ***p < 0.001; ****p < 0.0001; ns indicates no significance.

## Results

### Tumor-derived lactate suppresses *Pebp4* expression via H3K18 lactylation in lung CAC

**Fig. 1:**
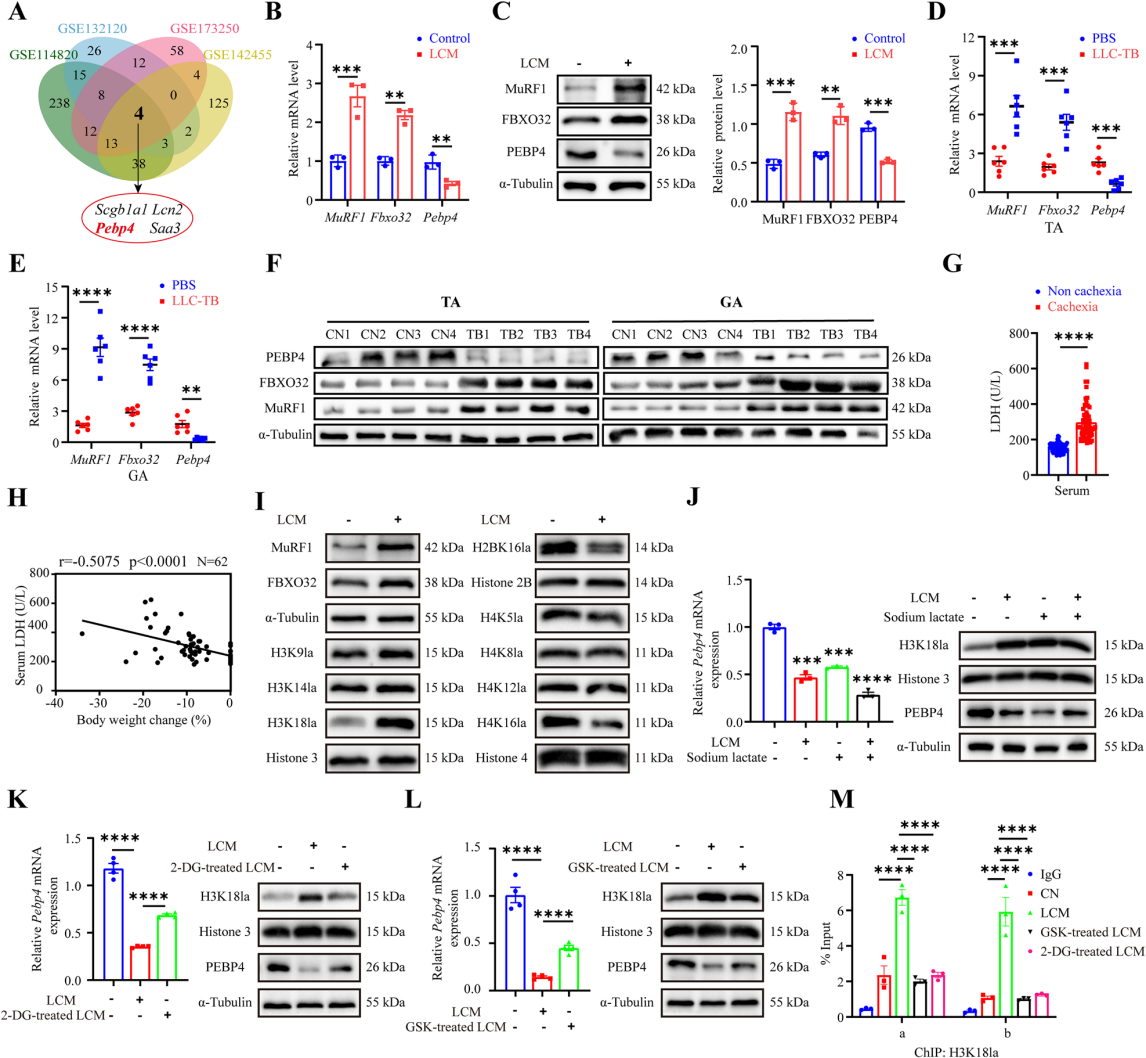
Tumor-derived lactate suppresses skeletal muscle *Pebp4* transcription via H3K18 lactylation in lung CAC. **A** Venn diagram showing overlap of four GEO RNA-seq datasets identifying candidate genes linked to CAC-induced muscle atrophy. qPCR (**B**) and western blot analyses (**C**) of MuRF1, FBXO32, and PEBP4 expression in C2C12 myotubes treated with LCM for 48 h. Relative *Pebp4* mRNA (**D**, **E**; *n* = 6 mice per group) and PEBP4 protein (**F**; *n* = 4 mice per group) levels in TA and GA muscles from LLC-TB mice. **G** Serum LDH activity in LUAD patients with cachexia (*n* = 62) versus non-cachectic patients with benign lung nodules (*n* = 62). **H** Linear regression showing a negative correlation between serum LDH levels and body weight change in LUAD cachexia patients (*n* = 62). **I** Western blot analysis of histone lactylation at multiple lysine residues in C2C12 myotubes treated with LCM, identifying H3K18 lactylation (H3K18la) as the most prominently altered modification. **J** *Pebp4* mRNA and PEBP4 protein levels in C2C12 myotubes treated with LCM or exogenous sodium lactate (50 mM) for 72 h. **K** *Pebp4* mRNA, PEBP4 protein, and H3K18la levels in C2C12 myotubes cultured with LCM from LLC cells treated with or without 2-DG. **L** *Pebp4* mRNA expression and representative Western blot analysis of PEBP4 and H3K18la in C2C12 myotubes cultured in LCM derived from LLC cells treated with or without GSK-2837808A. **M** ChIP–qPCR analysis of H3K18la occupancy at the *Pebp4* promoter in C2C12 myotubes exposed to conditioned medium from LLC cells treated with or without 2-DG or GSK-2837808A. The data are expressed as the mean ± SEM from at least three independent experiments (*p < 0.05, **p < 0.01, ***p < 0.001; ****p < 0.0001, ns = not significant).

To identify key regulators involved in CAC-induced muscle atrophy, we conducted an integrative analysis of four publicly available RNA-seq datasets (GSE132120 [37], GSE114820 [38], GSE173250 [39], and GSE142455 [40]). This analysis highlighted *Scgb1a1*, *Saa3*, *Lcn2*, and *Pebp4* as candidate genes potentially associated with CAC-related muscle wasting (Fig. 1A). To validate these bioinformatic findings, we established two complementary experimental models, including an in vitro myotube wasting model induced by treating differentiated C2C12 myotubes with LCM and an in vivo CAC mouse model established by subcutaneous injection of LLC cells into C57BL/6 mice. The LLC-TB mouse model of cancer cachexia was successfully established. Compared with PBS-injected control mice, LLC-TB mice exhibited significant body weight loss **(**Fig. S1A, B**)**, accompanied by marked reductions in the weights of eWAT, iWAT, GA, and TA muscles **(**Fig. S1C–F**)**. Histological analysis further revealed atrophy of both adipose and skeletal muscle tissues, as indicated by a significant decrease in cross-sectional area **(**Fig. S1G, H**)**.

Among the identified candidates, *Scgb1a1* and *Saa3* displayed inconsistent expression between LCM-treated myotubes and skeletal muscles of LLC-TB mice (Fig. S1I–K). Furthermore, small interfering RNA–mediated knockdown of *Saa3* or *Scgb1a1* in LCM-treated C2C12 myotubes did not significantly alter the expression of the cachexia-associated markers MuRF1 and FBXO32 (Fig. S1L–Q), suggesting a limited functional contribution of these genes to myotube wasting under cachectic conditions. In contrast, *Lcn2* was consistently upregulated in both in vitro and in vivo models (Fig. S1I, S1K), consistent with previous reports describing its involvement in cancer cachexia [41]. Notably, *Pebp4* exhibited the most consistent and pronounced downregulation at both mRNA and protein levels in LCM-treated myotubes (Fig. 1B, C) as well as in TA and GA muscles of LLC-TB mice (Fig. 1D–F), suggesting a potential role for *Pebp4* in lung CAC–related muscle wasting.

LCM contains proteins, nucleic acids, and metabolites, among other components. Previous studies have demonstrated that secreted proteins (e.g., TWEAK [42]) and tumor-derived miR-203a-3p [43] promote cachexia in muscle cells, while methionine metabolites have also been associated with cachexia [44]. However, the specific constituents of LCM that regulate *Pebp4* expression in muscle cells remain undefined. To identify the factors responsible for *Pebp4* downregulation, we employed the approach developed by Song’s laboratory [45], which involves boiling to denature and remove most proteins and nucleic acids. Accordingly, the LLC supernatant was boiled and subsequently reconstituted with fresh DMEM to generate the boiled LCM. This preparation was then used to treat C2C12 myotubes for analysis of *Pebp4* expression. The results indicated that, compared with the control group, *Pebp4* expression was significantly downregulated in both boiled and non-boiled LCM samples (Fig. S2A, B). These findings suggest that heat-stable metabolites may contribute to the regulation of *Pebp4* expression.

Untargeted metabolomic analysis of serum from LLC-TB mice and LUAD patients with cachexia revealed significantly elevated lactate levels compared with non-cachectic controls. These levels correlated with body weight loss, highlighting lactate as a key metabolite in cancer cachexia progression [46]. Consistent with these results, we observed increased serum LDH levels in patients with cachectic LUAD (Fig. 1G), which were inversely correlated with patients’ body weight loss (Fig. 1H). These data further support the role of lactate in lung cancer–associated cachexia and indicate that systemic lactate production may contribute to disease progression. Recent research has identified lactate as an epigenetic regulator that mediates histone and non-histone lactylation to modulate gene expression in both cancerous and non-cancerous cells [47]. Therefore, we propose that lactate secreted by LLC cells induces histone lactylation in skeletal muscle, thereby reducing *Pebp4* transcription. This hypothesis is supported by the elevated lactate concentrations detected in the serum of LLC-TB mice, in LCM, and in the GA and TA muscles of LLC-TB mice, as well as in LCM-treated myotubes (Fig. S2C–G).

To assess whether lactate directly mediates the repression of *Pebp4*, C2C12 myotubes were treated with exogenous sodium lactate (Fig. S3A). Sodium lactate at concentrations of 50–90 mM reduced *Pebp4* expression at both the mRNA and protein levels, whereas concentrations of 5–40 mM had no detectable effect **(**Fig. S3B, C**)**. We subsequently investigated whether lactate-induced *Pebp4* repression was associated with histone lactylation. LCM treatment increased global lysine lactylation (pan-Klac) levels in C2C12 myotubes (Fig. S3D). Among the previously reported histone lactylation sites examined, H3K18 lactylation (H3K18la) exhibited the most pronounced increase following LCM treatment (Fig. 1I). Treatment with sodium lactate, LCM, or their combination increased H3K18la levels and reduced *Pebp4* expression (Fig. 1J). Inhibition of lactate production in LLC cells using either 2-DG or the LDHA inhibitor GSK-2837808A decreased extracellular lactate concentrations, attenuated H3K18la levels in recipient myotubes, and restored *Pebp4* expression (Fig. 1K, L and S3E, F). Analysis of publicly available CUT&Tag data (GSE195856 [32]) revealed two prominent H3K18la enrichment peaks within the *Pebp4* promoter region (Fig. S3G). ChIP–qPCR analysis demonstrated increased H3K18la occupancy at the *Pebp4* promoter following LCM or sodium lactate treatment (Fig. S3H), whereas conditioned medium from LLC cells treated with 2-DG or GSK-2837808A resulted in reduced H3K18la enrichment (Fig. 1M). These findings indicate that tumor-derived lactate promotes H3K18la enrichment at the *Pebp4* promoter, thereby repressing *Pebp4* transcription in skeletal muscle cells.

### PEBP4 modulates atrophy-associated gene expression and muscle architecture both in vitro and in vivo

**Fig. 2:**
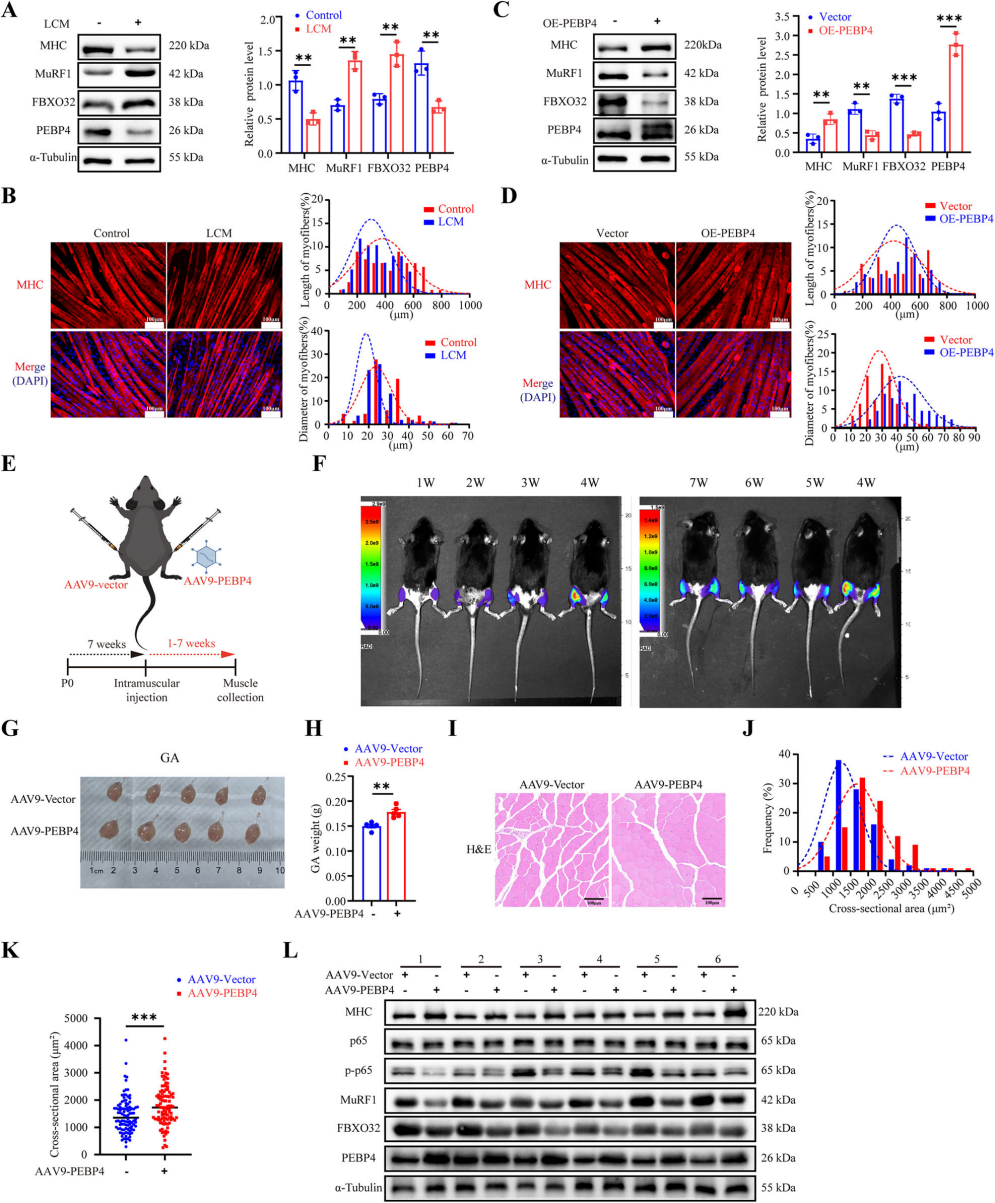
PEBP4 suppresses atrophy-related gene expression and preserves fiber morphology in vitro and in vivo. **A** Western blot analysis of MuRF1, FBXO32, MHC, and PEBP4 in C2C12 myotubes following LCM treatment. **B** Representative immunofluorescence images showing MHC (red) and DAPI-stained nuclei (blue), along with the quantification of myotube length and diameter after LCM treatment (scale bar = 100 μm). Western blot (**C**) and morphological analysis (**D**) of PEBP4-overexpressing myotubes (scale bar = 100 μm). **E** Schematic of intramuscular injection of AAV9-Vector or AAV9-PEBP4 into GA muscles of C57BL/6 mice. **F** Representative in vivo fluorescence imaging confirming stable expression of PEBP4 in GA muscles from week 1 to week 7 post-injection. Representative GA muscle images (**G**) and weights in AAV9-PEBP4–treated or AAV9-vector–treated mice (**H**; *n* = 5 mice per group). **I** Representative H&E staining of GA muscle cross-sections from AAV9-vector– or AAV9-PEBP4–injected mice (*n* = 3 mice per group, scale bar = 100 μm). **J**, **K** Quantitative assessment of myofiber cross-sectional area in GA muscles. **L** Western blot of MHC, total and phosphorylated p65, MuRF1, and FBXO32 in GA muscle lysates (*n* = 6 mice per group). The data are expressed as the mean ± SEM from at least three independent experiments (*p < 0.05, **p < 0.01, ***p < 0.001; ****p < 0.0001, ns = not significant).

To determine whether PEBP4 plays a functional role in maintaining skeletal muscle homeostasis, we established complementary cellular and animal models. In vitro, differentiated C2C12 myotubes exposed to LCM exhibited a pronounced decrease in PEBP4 expression, accompanied by significant upregulation of MuRF1 and FBXO32—canonical muscle-specific E3 ubiquitin ligases and key regulators of muscle atrophy—at the protein level (Fig. 2A). These molecular alterations corresponded with marked reductions in MHC expression and visible atrophic changes in myotube morphology, including decreased myotube diameter and length (Fig. 2B, S4A). Lentivirus-mediated overexpression of PEBP4, confirmed by elevated transgene expression in transduced myotubes (Fig. S4B), effectively reversed these pathological changes by restoring MHC expression, suppressing MuRF1 and FBXO32 expression (Fig. 2C), and improving myotube morphology (Fig. 2D, S4C).

In the mouse model, AAV9 vectors encoding PEBP4 were intramuscularly injected into the GA muscles of C57BL/6 mice (Fig. 2E). Sustained *Pebp4* expression was validated by in vivo fluorescence imaging over a 7-week period (Fig. 2F, S4D). Histological analysis revealed increased GA muscle mass (Fig. 2G, H) and enlarged myofiber cross-sectional area (Fig. 2I–K), indicating structural preservation and attenuation of muscle atrophy. Compared with GA muscles injected with control AAV9 vectors, PEBP4-overexpressing muscles exhibited significantly lower MuRF1 and FBXO32 expression, reduced phosphorylation of p65 (p-p65), and partial restoration of MHC protein levels (Fig. 2L).

Taken together, these findings suggest that PEBP4 functionally regulates atrophy-related gene expression and preserves muscle structural integrity under physiological conditions. These results provide a mechanistic basis for future studies investigating its potential involvement in lung CAC-induced muscle wasting.

### PEBP4 alleviates oxidative stress in skeletal muscle both in vitro and in vivo

**Fig. 3:**
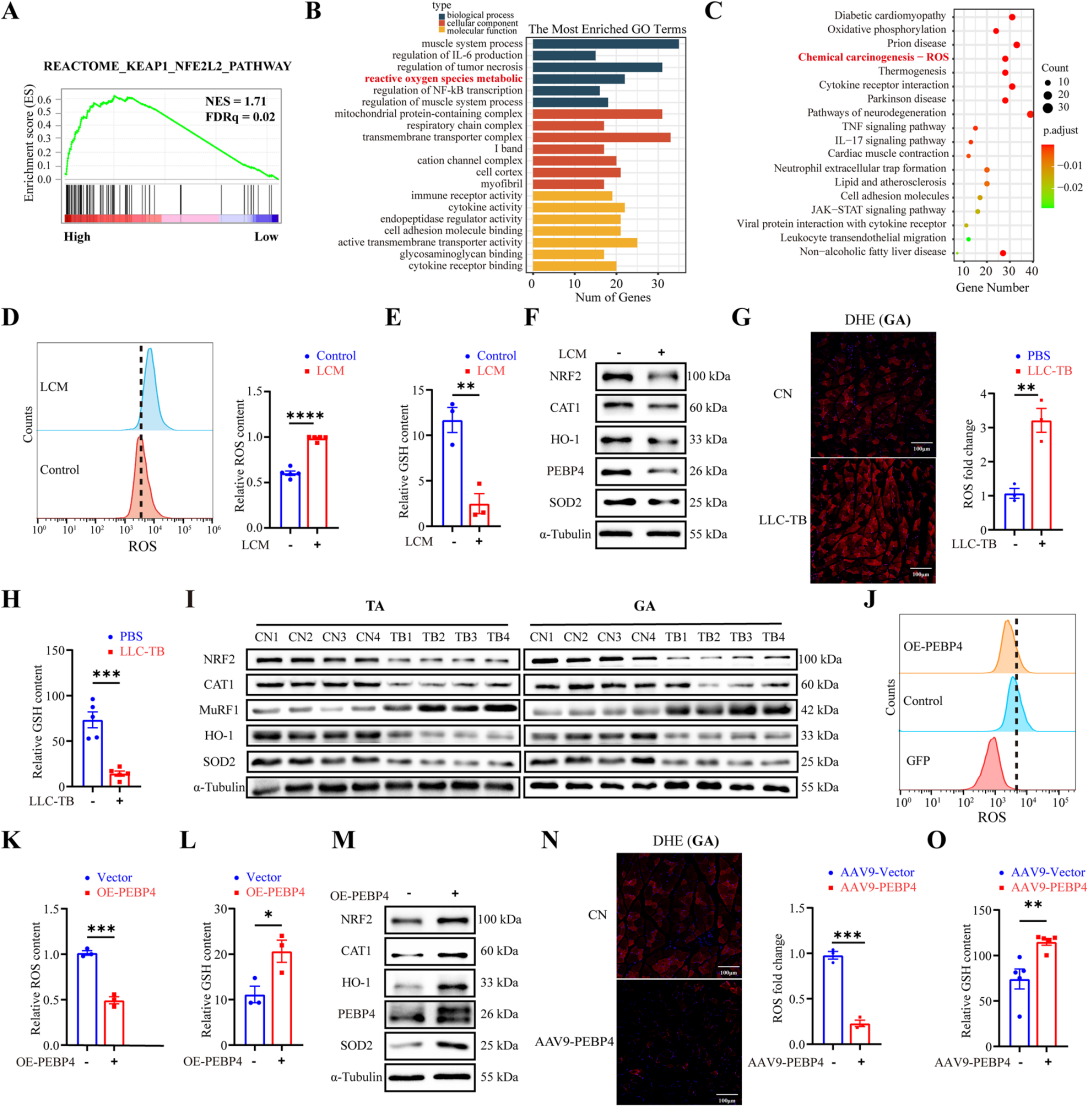
PEBP4 reduces oxidative injury in skeletal muscle cells and tissues. Bioinformatic analysis using GEO dataset revealed that PEBP4 expression was positively associated with ROS-related pathways, particularly the KEAP1-NRF2 axis, as shown by GSEA (**A**), GO (**B**), and KEGG (**C**) analyses. **D** Flow cytometry was used to quantify intracellular ROS levels in C2C12 myotubes treated with LCM for 48 h. **E** GSH levels in C2C12 myotubes treated with LCM for 48 h were measured using a commercial assay. **F** Western blot analysis of antioxidant defense components in LCM-treated myotubes, including NRF2, HO-1, SOD2, and CAT1. **G** ROS levels in the GA muscle of LLC-TB mice were assessed by dihydroethidium (DHE) fluorescence intensity (*n* = 3 mice per group, scale bar = 100 μm). **H** GSH levels in the GA muscle of LLC-TB mice were quantified using a commercial assay (*n* = 5 mice per group). **I** Western blot analysis showing the downregulation of NRF2 pathway proteins in the TA and GA muscles of PBS- and LLC-TB-injected mice (*n* = 4 mice per group). PEBP4 overexpression in C2C12 myotubes reduced ROS accumulation (**J**, flow cytometry; **K**, bar graph) and restored intracellular GSH levels (**L**), while activating NRF2 signaling (**M**). In vivo AAV9-PEBP4 delivery into GA muscles significantly reduced ROS accumulation (**N**; *n* = 3 mice per group, scale bar = 100 μm) and restored GSH levels (**O**; *n* = 5 mice per group). The data are expressed as the mean ± SEM from at least three independent experiments (*p < 0.05, **p < 0.01, ***p < 0.001; ****p < 0.0001, ns = not significant).

To elucidate the molecular mechanisms underlying PEBP4-mediated muscle preservation, integrative enrichment analyses were performed using the GSE114820 dataset [38]. GSEA, GO, and KEGG pathway analyses revealed a convergent association between PEBP4 expression and oxidative stress–related pathways, including the KEAP1–NRF2 antioxidant axis, ROS metabolic processes, and chemical carcinogenesis pathways involving ROS (Fig. 3A–C).

In vitro, differentiated C2C12 myotubes treated with LCM exhibited a significant increase in intracellular ROS levels (Fig. 3D) and depletion of GSH (Fig. 3E), accompanied by downregulation of PEBP4, NRF2, and key antioxidant enzymes, including catalase (CAT1), heme oxygenase-1 (HO-1), and superoxide dismutase 2 (SOD2) (Fig. 3F, S5A). A comparable pattern of redox imbalance was observed in the skeletal muscles of LLC-TB mice, characterized by elevated ROS accumulation (Fig. 3G), decreased GSH content (Fig. 3H), and reduced expression of NRF2 and its downstream antioxidant targets (Fig. 3I and S5B, C).

PEBP4 overexpression in C2C12 myotubes markedly mitigated ROS accumulation (Fig. 3J, K), restored GSH levels (Fig. 3L), and reactivated the NRF2-driven antioxidant defense program (Fig. 3M and S5D, E). However, it did not significantly alter the mRNA levels of NADPH oxidase family members *Nox4*, *Duox1*, and *Duox2* (Fig. S5F). Consistently, AAV9-mediated PEBP4 delivery in vivo alleviated oxidative damage in GA muscles, as indicated by decreased ROS levels (Fig. 3N), restored GSH content (Fig. 3O), and enhanced expression of NRF2 and SOD2 (Fig. S5G, H). Collectively, these findings demonstrate that PEBP4 protects skeletal muscle from oxidative injury by reestablishing redox homeostasis through NRF2-mediated antioxidant pathways under lung CAC conditions.

### PEBP4 alleviates LCM-induced oxidative stress and myotube atrophy with partial dependence on NRF2 signaling

Given the observed correlation between PEBP4 and antioxidant pathways, GSEA identified the KEAP1–NRF2 signaling axis among PEBP4-associated networks (Fig. 3A–C). Consistently, NRF2 and its downstream antioxidant targets—including CAT1, HO-1, and SOD2—were markedly upregulated in PEBP4-overexpressing myotubes and in skeletal muscles of mice administered AAV9-PEBP4, respectively (Fig. 3M and S5D–H). These findings prompted further investigation into the role of NRF2 signaling in mediating the antioxidative and anti-atrophic effects of PEBP4.

To determine the contribution of NRF2 signaling to PEBP4-mediated protection, LCM-treated myotubes were used along with complementary oxidative stress models involving hydrogen peroxide (H_2_O_2_) exposure and pharmacological inhibition of NRF2 by ML385. These experimental approaches enabled assessment of the extent to which NRF2 signaling contributes to the protective effects associated with PEBP4 expression.

**Fig. 4:**
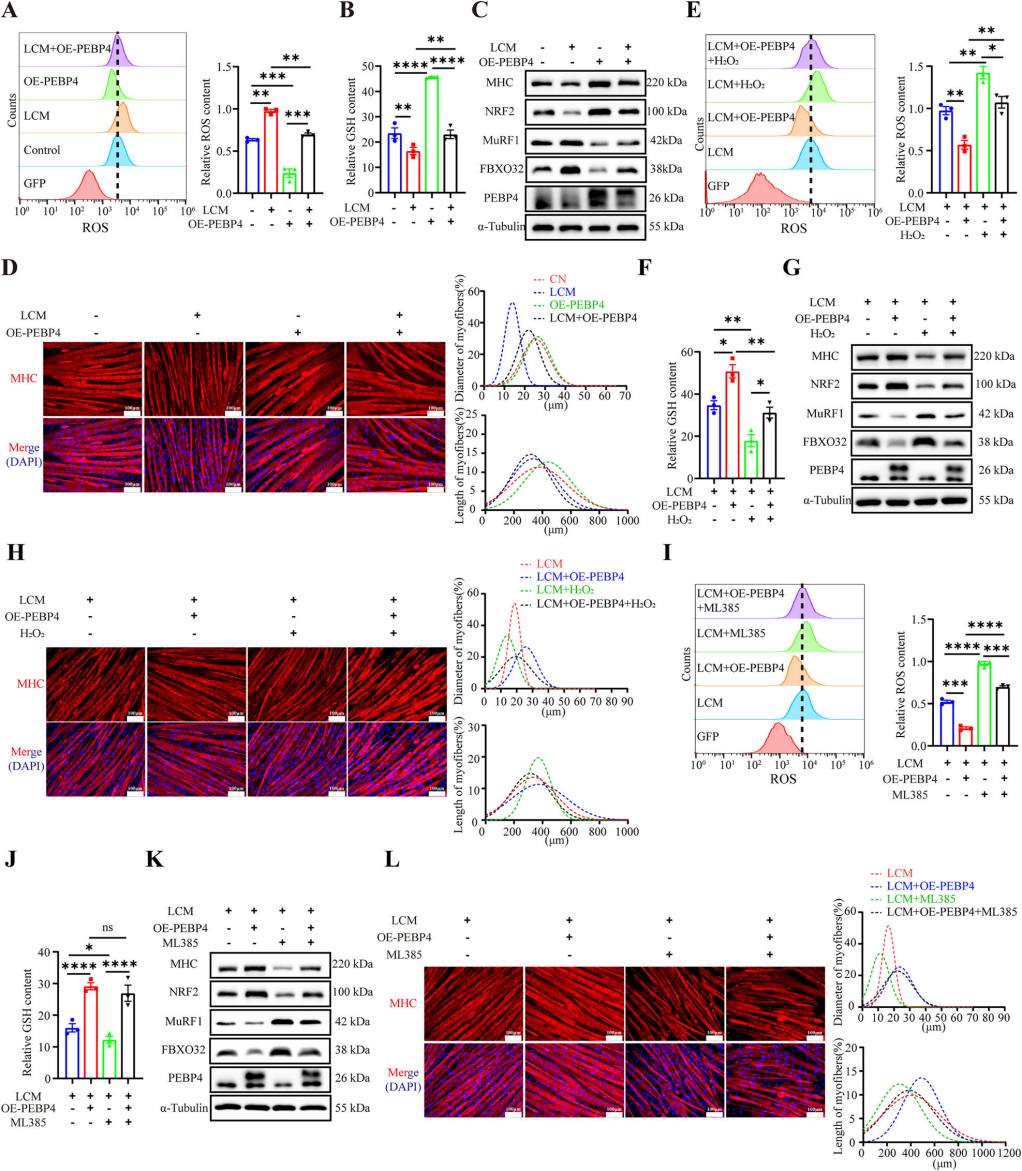
PEBP4 restores redox homeostasis and attenuates LCM-induced atrophy via NRF2 pathway. **A**–**D** C2C12 myotubes were treated with LCM for 48 h, with or without PEBP4 overexpression, to assess its antioxidative effects. ROS levels were quantified by flow cytometry (**A**), and GSH content was measured using a commercial assay (**B**). Western blot analysis showed increased expression of NRF2 and MHC, along with decreased levels of MuRF1 and FBXO32 (**C**). MHC (red) immunofluorescence staining demonstrated preserved myotube morphology and an increase in diameter (**D**; scale bar = 100 μm). To investigate the antioxidative role of PEBP4 under exogenous oxidative stress, myotubes were exposed to H₂O₂ (100 μM, 48 h) in the LCM-induced atrophy model. ROS accumulation was measured by flow cytometry (**E**), while GSH levels were quantified using a commercial assay (**F**). Western blot analysis revealed expression levels of NRF2, MHC, MuRF1, and FBXO32 (**G**). Myotube morphology was visualized by MHC (red) immunofluorescence (**H**; scale bar = 100 μm). To explore NRF2 involvement, ML385 (20 μM) was applied to PEBP4-overexpressing myotubes in LCM-treated C2C12 myotubes. ROS levels were measured by flow cytometry (**I**), GSH levels were measured using a commercial assay (**J**), and changes in NRF2, MHC, MuRF1 and FBXO32 expression were assessed by Western blot (**K**). MHC (red)/DAPI (blue) staining was used to visualize myotube morphology (**L**; scale bar = 100 μm). The data are expressed as the mean ± SEM from at least three independent experiments (*p < 0.05, **p < 0.01, ***p < 0.001; ****p < 0.0001, ns = not significant).

In LCM-treated myotubes, PEBP4 overexpression significantly reduced ROS accumulation (Fig. 4A) and elevated intracellular GSH levels (Fig. 4B). These biochemical changes were accompanied by increased expression of NRF2 and MHC, as well as decreased expression of the atrophy-associated genes *MuRF1* and *Fbxo32* (Fig. 4C, S6A). Morphological evaluation further demonstrated preservation of myotube integrity, indicated by increased fiber diameter (Fig. 4D, S6B). Collectively, these results support the conclusion that PEBP4 protects myotubes from LCM-induced oxidative stress and atrophy under lung CAC conditions.

Under H_2_O_2_-induced oxidative stress (Fig. S6C), PEBP4 overexpression decreased ROS levels (Fig. 4E) and restored intracellular GSH concentrations (Fig. 4F). Correspondingly, NRF2 and MHC expression remained elevated, whereas MuRF1 and FBXO32 expression decreased (Fig. 4G, S6D). Immunofluorescence analysis illustrated preservation of myotube morphology, as evidenced by fiber elongation and hypertrophy (Fig. 4H, S6E).

To evaluate the contribution of NRF2 signaling, ML385 (Fig. S6F) was used to inhibit NRF2 activity in PEBP4-overexpressing myotubes under LCM-induced atrophy conditions. Under NRF2 inhibition, PEBP4 overexpression reduced ROS levels (Fig. 4I), increased GSH concentrations (Fig. 4J), and mitigated atrophy-related gene expression (Fig. 4K, S6G), although the magnitude of these effects was attenuated compared with conditions without ML385 treatment. Immunofluorescence analysis further demonstrated preservation of myotube morphology in the presence of ML385 (Fig. 4L, S6H). Collectively, these results suggest that PEBP4-driven protection is at least partially mediated through NRF2 signaling.

### PEBP4 stabilizes NRF2 by interfering with KEAP1-CUL3-mediated ubiquitination

**Fig. 5:**
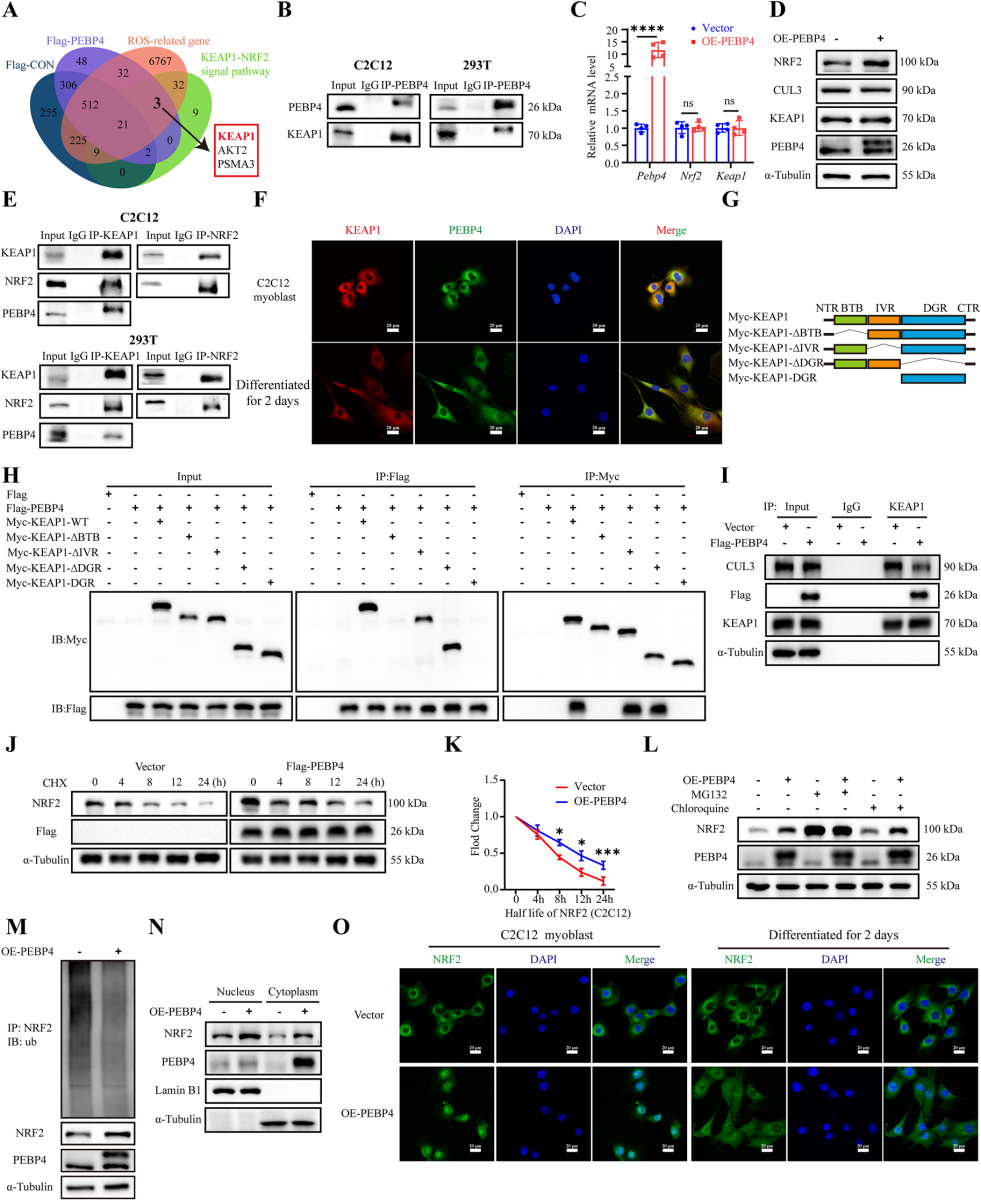
PEBP4 promotes NRF2 nuclear translocation and antioxidant signaling by disrupting KEAP1-mediated ubiquitination. **A** Venn diagram illustrated the overlap between ROS-related genes and KEAP1-NRF2 pathway genes in cells with high PEBP4 expression. **B** Co-IP analysis showing physical interactions between PEBP4 and KEAP1 in C2C12 and HEK293T cells. qPCR (**C**) and Western blot (**D**) analyses of KEAP1 pathway components in PEBP4-overexpressing myotubes. **E** Co-IP analysis demonstrated interactions among PEBP4, KEAP1, and NRF2 in C2C12 and HEK293T cells. **F** Confocal microscopy showed cytoplasmic co-localization of KEAP1 (red) and PEBP4 (green) in both undifferentiated and 2-day differentiated C2C12 cells. Nuclei were counterstained with DAPI (blue) (scale bar = 20 μm). Schematic representation of KEAP1 truncation constructs (**G**) and Co-IP analysis (**H**) demonstrating that the BTB domain serves as the specific binding interface for PEBP4. **I** Co-IP analysis showing reduced binding affinity between KEAP1 and CUL3 upon PEBP4 overexpression, indicating disruption of the KEAP1-CUL3 complex by PEBP4. CHX chase assay (**J**) and half-life quantification (**K**) confirmed prolonged NRF2 protein stability upon PEBP4 overexpression myotubes. **L** Western blot analysis of NRF2 levels in PEBP4-overexpressing myotubes treated with MG132 (proteasome inhibitor) or CQ (lysosomal inhibitor). **M** NRF2 ubiquitination assay showing reduced polyubiquitination of NRF2 following PEBP4 overexpression myotubes, as detected by NRF2 immunoprecipitation. Nuclear–cytoplasmic fractionation assays (**N**) and confocal immunofluorescence analysis (**O**; scale bar = 20 μm) were performed to evaluate NRF2 subcellular localization following PEBP4 overexpression. In (**O**), NRF2 is shown in green and nuclei are counterstained with DAPI (blue). The data are expressed as the mean ± SEM from at least three independent experiments (*p < 0.05, **p < 0.01, ***p < 0.001; ****p < 0.0001, ns = not significant).

Given the central role of NRF2 signaling in PEBP4-associated protection against lung CAC–induced muscle wasting, we next investigated how PEBP4 modulated NRF2 stability at the molecular level. GSEA indicated the REACTOME_KEAP1_NFE2L2_PATHWAY (https://www.reactome.org/content/detail/R-MMU-9755511) as a significantly enriched pathway among PEBP4-associated signatures. Supporting this finding, proteomic profiling of PEBP4-overexpressing C2C12 myotubes identified KEAP1 as the top-ranking redox-associated binding partner, reinforcing its potential role in NRF2 regulation (Fig. 5A, S7A). Molecular docking analysis predicted a strong binding affinity between PEBP4 and KEAP1 (ΔG = –20.0 kcal/mol), exceeding those predicted for PEBP4–PSMA3 (–14.9 kcal/mol) and PEBP4–AKT2 (–4.7 kcal/mol) interactions (Fig. S7A). Co-immunoprecipitation (Co-IP) confirmed a specific interaction between PEBP4 and KEAP1 in both C2C12 and HEK293T cells (Fig. 5B). Notably, PEBP4 overexpression did not alter *Keap1* or *Nrf2* transcript levels (Fig. 5C) or significantly affect KEAP1 or CUL3 protein abundance (Fig. 5D, S7B), indicating that PEBP4 may regulate NRF2 at the post-translational level.

To exclude the involvement of the AKT2–GSK-3β–β-TrCP axis, we examined the phosphorylation of AKT2 (S474), GSK-3β (S9), and NRF2 (S40). PEBP4 overexpression did not significantly affect these phosphorylation events (Fig. S7C), and pharmacological inhibition of AKT2 did not modify NRF2-related responses in PEBP4-overexpressing myotubes (Fig. S7D), suggesting that this pathway is unlikely to mediate the observed effects. Further analyses demonstrated that PEBP4 associated with KEAP1 but not with NRF2 (Fig. 5E, S7E), and this interaction was substantiated by exogenous Co-IP (Fig. S7F). To further characterize the PEBP4–KEAP1 association, we performed confocal imaging on undifferentiated C2C12 myoblasts and 2-day differentiated C2C12 cells, following the methods described by Zhao et al. [48] and Li et al. [49]. The results revealed cytoplasmic colocalization of PEBP4 and KEAP1 (Fig. 5F). To identify the interaction domain, a series of KEAP1 truncation constructs were generated (full-length, ∆BTB, ∆IVR, ∆DGR, and DGR). PEBP4 was found to bind the BTB domain of KEAP1, which is necessary for CUL3 recruitment (Fig. 5G, H, S7G). Consistent with this finding, PEBP4 overexpression decreased the interaction between KEAP1 and CUL3 (Fig. 5I), suggesting interference with KEAP1–CUL3 complex formation. CHX chase assays demonstrated an extended half-life of NRF2 in PEBP4-overexpressing myotubes (Fig. 5J, K). Moreover, treatment with MG132—but not with chloroquine—resulted in NRF2 accumulation (Fig. 5L), indicating proteasome-dependent regulation. Ubiquitination assays further confirmed that PEBP4 overexpression reduced NRF2 ubiquitination in C2C12 myotubes (Fig. 5M). To assess the functional consequence of NRF2 stabilization, we examined NRF2 subcellular localization. Nuclear–cytoplasmic fractionation (Fig. 5N) and confocal immunofluorescence analysis (Fig. 5O) demonstrated increased nuclear accumulation of NRF2 in PEBP4-overexpressing myotubes.

To determine whether specific residues within the KEAP1 BTB domain are required for PEBP4-mediated NRF2 stabilization, we generated a panel of KEAP1 point mutants targeting conserved BTB residues (C77S, C151S, Y85F, S104A, and S172A) (Fig. S8A). These residues were selected based on sequence conservation and previously reported functional relevance [50–53]. Using KEAP1-deficient C2C12 cells reconstituted with either wild-type or mutant KEAP1 constructs, we evaluated NRF2 protein levels upon PEBP4 overexpression. Reconstitution with wild-type KEAP1 restored PEBP4-mediated NRF2 stabilization. In contrast, mutation of cysteine 151 markedly reduced this effect, whereas mutations at other BTB domain residues did not significantly alter NRF2 stabilization (Fig. S8B). Ubiquitination assays showed that the inhibitory effect of PEBP4 on NRF2 ubiquitination was largely diminished in cells expressing the C151S mutant (Fig. S8C). Co-immunoprecipitation assays further indicated that the C151S mutation reduced the interaction between PEBP4 and KEAP1 (Fig. S8D).

Collectively, these findings suggest that PEBP4 stabilizes NRF2 by disrupting KEAP1–CUL3–mediated ubiquitination, thereby promoting its nuclear accumulation and supporting NRF2-driven antioxidant signaling under lung cancer cachexia–associated stress.

### AAV9-mediated PEBP4 delivery attenuates muscle wasting and oxidative stress in a murine model of lung CAC

**Fig. 6:**
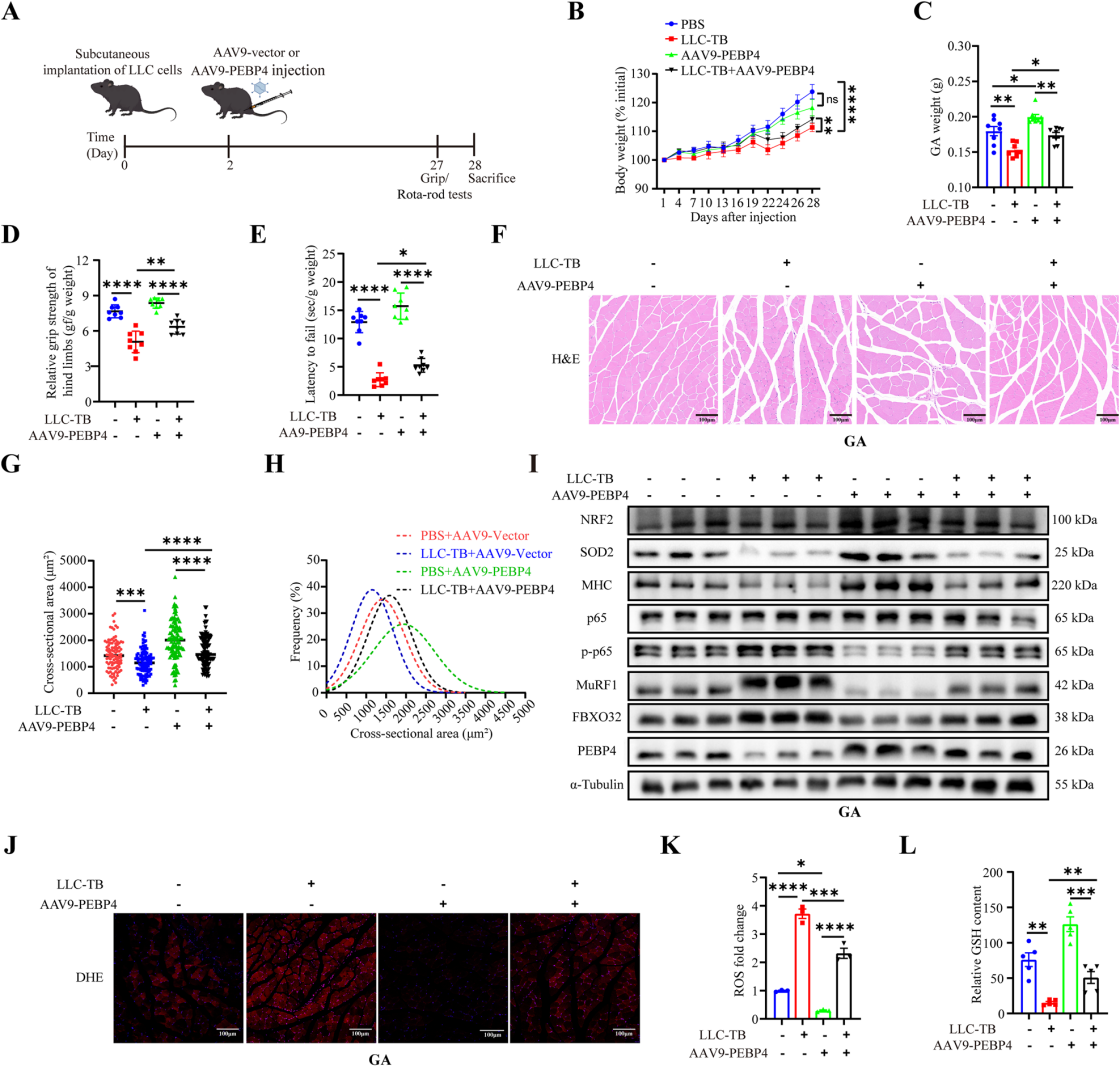
PEBP4 overexpression ameliorates muscle atrophy and ROS accumulation in lung CAC. **A** Experimental design illustrating intramuscular delivery of AAV9-PEBP4 into the GA muscle following LLC tumor implantation. Assessment of body weight change (**B**), GA muscle weight (**C**), hindlimb grip strength (**D**), and rotarod performance (**E**) in each group (*n* = 8 mice per group). H&E staining (**F**) and quantification of myofiber cross-sectional area (**G**) and frequency (**H**) in the GA muscles of each group (*n* = 3 mice per group, scale bar = 100 μm). **I** Western blot analysis of GA muscle lysates detecting PEBP4, NRF2, SOD2, phosphorylated p65 (p-p65), MuRF1, FBXO32, and MHC (*n* = 6 mice per group). **J** ROS levels in GA tissue were assessed by DHE staining (*n* = 3 mice per group, scale bar = 100 μm). **K** Quantification of ROS levels in GA muscles based on DHE fluorescence staining. **L** Quantification of GSH content in GA tissues (*n* = 5 mice per group). The data are expressed as the mean ± SEM from at least three independent experiments (*p < 0.05, **p < 0.01, ***p < 0.001; ****p < 0.0001, ns = not significant).

To assess the therapeutic efficacy of PEBP4 in vivo, AAV9-PEBP4 was intramuscularly administered into the GA muscle of LLC-TB mice (Fig. 6A). PEBP4 gene delivery significantly mitigated lung CAC-induced body weight loss (Fig. 6B, S9A), preserved skeletal muscle mass (Fig. 6C, S9B), and enhanced functional performance. This functional improvement was supported by increased grip strength and extended rotarod endurance (Fig. 6D, E, S9C). Histological examination demonstrated an enlargement of myofiber cross-sectional area and a shift toward larger fiber sizes (Fig. 6F–H), findings consistent with structural preservation and reduced muscle wasting in PEBP4-treated LLC-TB mice.

In addition, Western blot analysis revealed enhanced activation of the NRF2–SOD2 antioxidant axis, suppression of NF-κB signaling (p-p65), decreased expression of the muscle atrophy markers MuRF1 and FBXO32, and maintenance of MHC protein levels (Fig. 6I). AAV9-PEBP4 administration also markedly mitigated oxidative stress in the GA muscle, as evidenced by reduced DHE fluorescence (Fig. 6J, K) and restored GSH levels (Fig. 6L). Collectively, these results demonstrate that AAV9-mediated PEBP4 overexpression protects against lung CAC–induced muscle degeneration by strengthening antioxidant defenses and suppressing catabolic remodeling processes.

### NRF2 inhibition compromises PEBP4-mediated protection against muscle wasting and oxidative stress in lung CAC

**Fig. 7:**
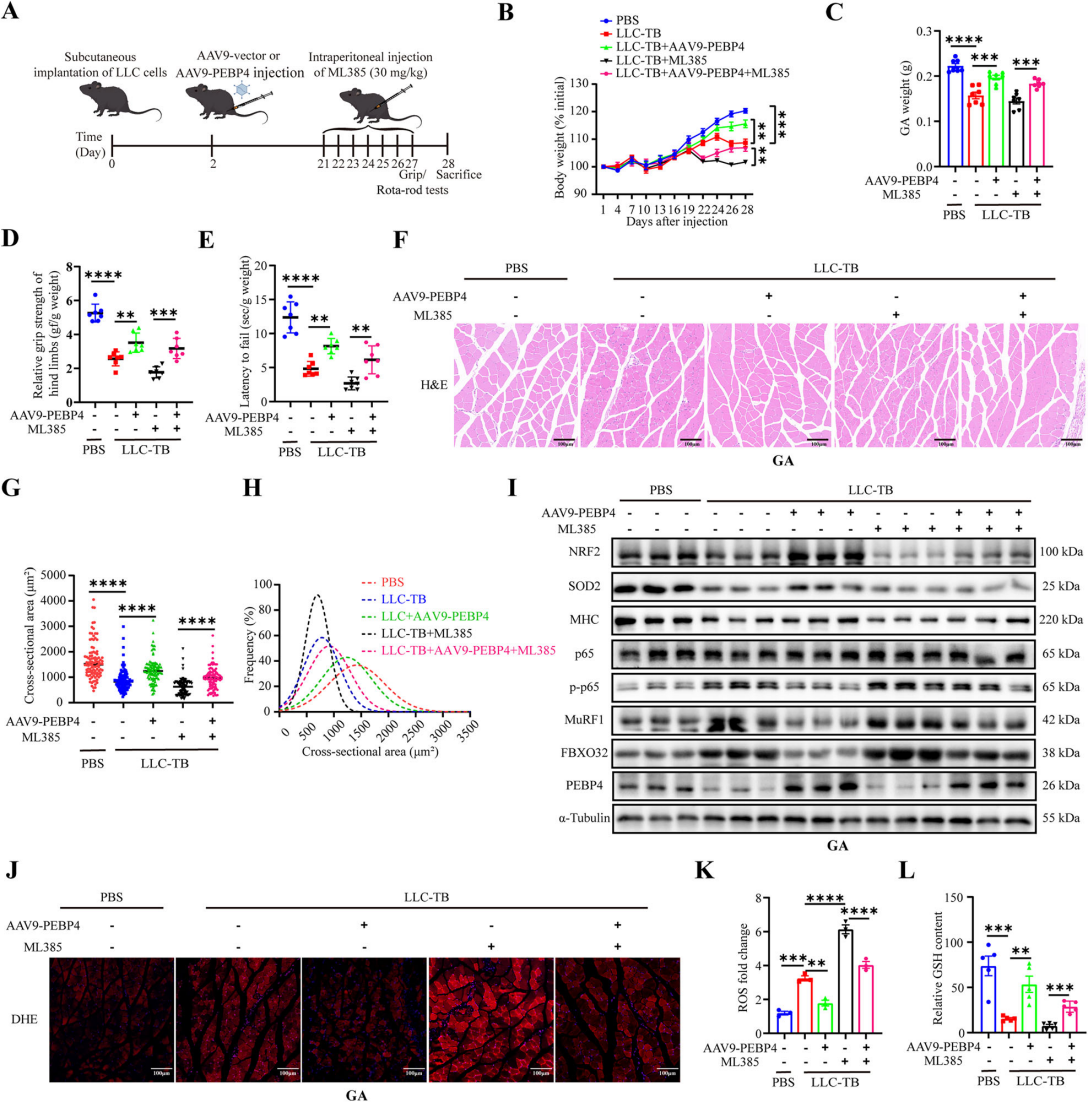
NRF2 inhibition exacerbates muscle atrophy and oxidative stress in PEBP4-overexpressing lung CAC models. **A** Schematic of the in vivo experimental design. LLC-TB mice were treated with AAV9-PEBP4, the NRF2 inhibitor ML385, or vehicle control. Assessment of cachexia-related phenotypes, including body weight change (**B**), GA muscle weight (**C**), hindlimb grip strength (**D**), and rotarod performance (**E**) (*n* = 7 mice per group). Representative H&E staining (**F**; *n* = 3 mice per group, scale bar = 100 μm) and quantification of GA muscle fiber cross-sectional area (**G**) and frequency (**H**) in each group. **I** Western blot analysis of GA muscle lysates showed modulation of NRF2-SOD2 signaling, NF-κB activity (p-p65), and expression of the E3 ligases MuRF1 and FBXO32 (*n* = 3 mice per group). DHE fluorescence imaging (**J**; *n* = 3 mice per group, scale bar = 100 μm) of GA muscle to assess ROS levels and quantification of fluorescence intensity (**K**). **L** Quantification of GSH levels in GA tissues (*n* = 5 mice per group). The data are expressed as the mean ± SEM from at least three independent experiments (*p < 0.05, **p < 0.01, ***p < 0.001; ****p < 0.0001, ns not significant).

To further elucidate the functional role of NRF2 signaling in PEBP4-mediated protection in vivo, we established a combined intervention model by co-administering AAV9-PEBP4 with the NRF2 inhibitor ML385 in LLC-TB mice (Fig. 7A). Despite NRF2 inhibition, PEBP4 overexpression partially mitigated ML385-exacerbated body weight loss (Fig. 7B, S9D) and muscle mass reduction (Fig. 7C, S9E). However, the overall magnitude of protection was reduced compared with conditions without ML385 treatment. Consistent with these findings, PEBP4-treated LLC-TB mice exhibited improved muscle performance, as indicated by enhanced grip strength (Fig. 7D, S9F) and increased rotarod endurance (Fig. 7E), although these improvements were attenuated under NRF2 inhibition. Histological analyses showed preservation of myofiber cross-sectional area and a rightward shift in fiber size distribution despite NRF2 inhibition (Fig. 7F–H). At the molecular level, PEBP4 partially restored NRF2–SOD2 signaling activity and reduced NF-κB activation, as evidenced by decreased p-p65 levels and downregulation of the catabolic effectors MuRF1 and FBXO32 (Fig. 7I). Even under pharmacological NRF2 inhibition by ML385, PEBP4 maintained antioxidant capacity, demonstrated by lower ROS levels (Fig. 7J, K**)** and higher GSH concentrations (Fig. 7L). Collectively, these results suggest that PEBP4-mediated protection is at least partially dependent on NRF2 signaling.

## Discussion

**Fig. 8:**
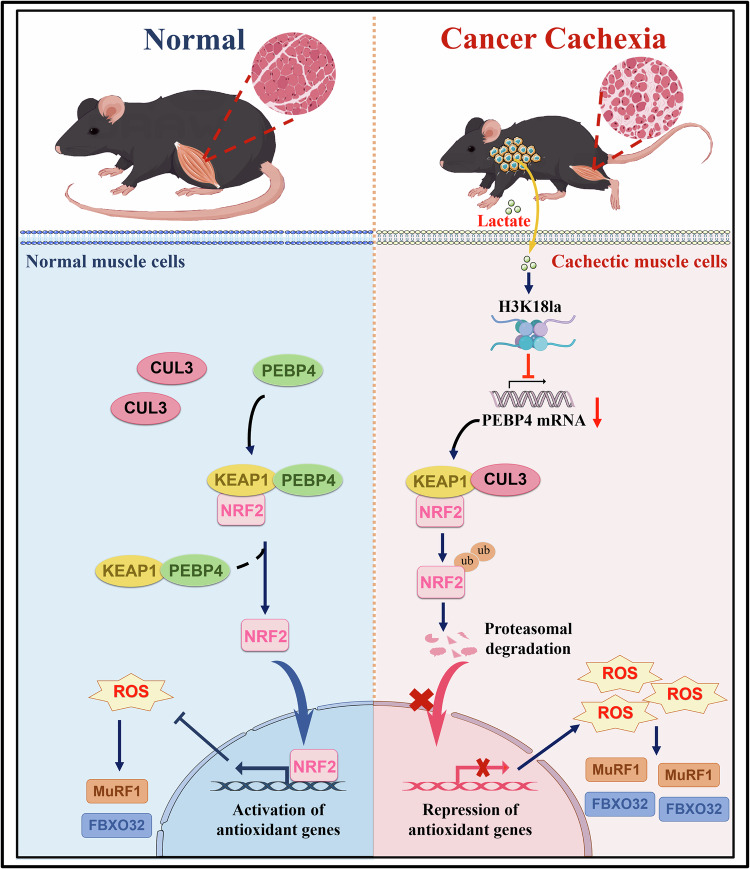
Proposed model of the tumor lactate–H3K18la–PEBP4–KEAP1–NRF2 signaling axis in lung CAC. Tumor-derived lactate enhances H3K18la occupancy at the *Pebp4* promoter, resulting in repression of *Pebp4* transcription. Reduced PEBP4 expression enhances KEAP1–CUL3-mediated ubiquitination and degradation of NRF2, limiting its nuclear accumulation and downstream antioxidant gene activation. The resulting redox imbalance promotes ROS accumulation and upregulation of muscle atrophy–associated E3 ubiquitin ligases, including MuRF1 and FBXO32, ultimately contributing to skeletal muscle wasting.

Our study identifies PEBP4 as a muscle-intrinsic regulator that protects against lung CAC by preserving redox homeostasis. We demonstrate that tumor-derived lactate suppresses PEBP4 expression through enrichment of histone H3K18 lactylation at the *Pebp4* promoter, thereby linking tumor metabolic reprogramming to skeletal muscle redox imbalance. Reduced expression of PEBP4 compromises NRF2-mediated antioxidant defense, leading to oxidative stress, which subsequently activates E3 ubiquitin ligases MuRF1 and FBXO32, promoting muscle wasting. Together, these findings establish a tumor lactate–PEBP4–KEAP1–NRF2 signaling axis that connects tumor metabolism to redox imbalance and muscle degeneration in lung CAC (Fig. 8).

Lactate accumulation is a hallmark of tumor aerobic glycolysis (the Warburg effect) [54] and has been implicated in the pathogenesis of cancer cachexia [46, 55]. Previous studies have linked lactate to adipose tissue wasting via GPR81-mediated signaling in CAC [46]. Consistent with these observations, in cohort of 62 patients with LUAD, we observed a significant association between LDH levels and body weight change, providing preliminary clinical support for a role of lactate in cachexia-related tissue wasting. Further investigation in large, well-designed prospective clinical cohorts will provide more robust and definitive clinical evidence. At the mechanistic level, our findings further extend this framework by demonstrating that tumor-derived lactate exerts direct epigenetic effects in skeletal muscle, suggesting that lactate not only reflects metabolic imbalance but also functions as an epigenetic regulator of skeletal muscle gene expression in lung CAC.

Oxidative stress has been increasingly recognized as a key contributor to muscle degeneration across diverse pathological conditions [14–17, 56]. Excessive ROS accumulation activates FOXO and NF-κB signaling while suppressing anabolic pathways, thereby promoting proteolysis. Our findings reveal that PEBP4 enhances NRF2 stability, restores antioxidant gene expression, and attenuates muscle atrophy–associated transcriptional programs. Inhibition of NRF2 attenuates, but does not abolish, PEBP4-mediated protection, indicating that NRF2 plays an important role in its protective effects against oxidative and atrophic stress. Additional signaling pathways, potentially including ERK or p38, may also participate in PEBP4-mediated protection, although their precise contributions warrant further investigation.

Our findings uncover a non-classical mechanism of NRF2 stabilization in which PEBP4 engages the BTB domain of KEAP1 and disrupts KEAP1–CUL3 complex assembly without altering KEAP1–NRF2 binding. Whereas the DGR domain of KEAP1 mediates NRF2 recognition and binding, the BTB domain facilitates CUL3 recruitment and E3 ubiquitin ligase assembly [21]. By acting at the level of E3 ligase complex assembly rather than NRF2 binding, PEBP4 targets a distinct regulatory step within the NRF2 ubiquitination pathway. In contrast, the most clinically advanced NRF2 activators—including sulforaphane, dimethyl fumarate, and omaveloxolone—are electrophilic compounds that covalently modify KEAP1 cysteine residues, particularly Cys151, thereby impairing KEAP1-mediated NRF2 degradation [57, 58]. Despite demonstrated clinical efficacy, this covalent mechanism lacks target specificity [59] and may induce off-target redox perturbation [60]. Small molecules that disrupt the KEAP1–NRF2 interaction at the DGR domain are under investigation, although their clinical translation remains limited [57]. By disrupting KEAP1–CUL3 assembly rather than covalently modifying KEAP1 cysteine residues or directly blocking KEAP1–NRF2 binding, PEBP4 stabilizes NRF2 through a mechanism that may reduce off-target redox perturbation, potentially representing a therapeutic approach with improved specificity in cancer-associated muscle wasting.

In addition to KEAP1-dependent regulation, NRF2 can also be regulated via GSK-3β/β-TrCP–mediated degradation [22, 23]. Our data do not support a significant role for the PI3K–AKT pathway in this context. Neither PEBP4 overexpression nor AKT2 inhibition alters AKT2 or GSK-3β phosphorylation in C2C12 cells. Consistent with prior studies showing that PEBP4 modulates myogenic processes through ERK rather than PI3K–AKT signaling [61], these findings further support a context-specific signaling profile and reinforce PEBP4 as a selective modulator of NRF2 stability in skeletal muscle.

For in vivo delivery, AAV9 was selected based on its well-established muscle tropism and capacity for sustained transgene expression in skeletal muscle [62, 63]. Consistent with previous studies demonstrating the therapeutic potential of AAV9 in muscle disorders [34, 62, 64–66], AAV9-mediated PEBP4 delivery attenuated muscle wasting in the lung CAC model. Advances in AAV vector engineering, including the development of muscle-targeted capsid variants, have improved muscle specificity and transduction efficiency, allowing lower systemic vector doses [67]. These developments may further enhance the translational potential of PEBP4-based gene therapy for cancer-associated cachexia.

Collectively, our study establishes PEBP4 as a redox-modulating effector linking tumor metabolic stress to skeletal muscle degeneration. We identify a non-classical mechanism in which PEBP4 disrupts KEAP1–CUL3 assembly at the BTB domain, resulting in NRF2 stabilization without interfering with KEAP1–NRF2 binding. This mechanistic distinction highlights the therapeutic potential of targeting the tumor-derived lactate–PEBP4–KEAP1–NRF2 axis in cancer-associated muscle wasting and supports further development of gene- and pharmacology-based strategies for clinical translation.

## Supplementary information

Figure S1

Figure S2

Figure S3

Figure S4

Figure S5

Figure S6

Figure S7

Figure S8

Figure S9

Supplementary materials

Original western blots

## Data Availability

Transcriptional and CUT&Tag data used in this study were obtained from the Gene Expression Omnibus database. Additional data generated during the study are available from the corresponding author upon reasonable request. Full Western blot images and additional supporting data are available in the Supplementary Material.
