# Supplementary material for: PEBP4 alleviates muscle wasting in lung cancer cachexia via KEAP1–NRF2-mediated redox homeostasis
Source: Cell Death Dis. 2026 Jun 3;17(1):692. doi: 10.1038/s41419-026-08925-5 (PMC13443934; doi:10.1038/s41419-026-08925-5)

Figure 1

Figure 1C

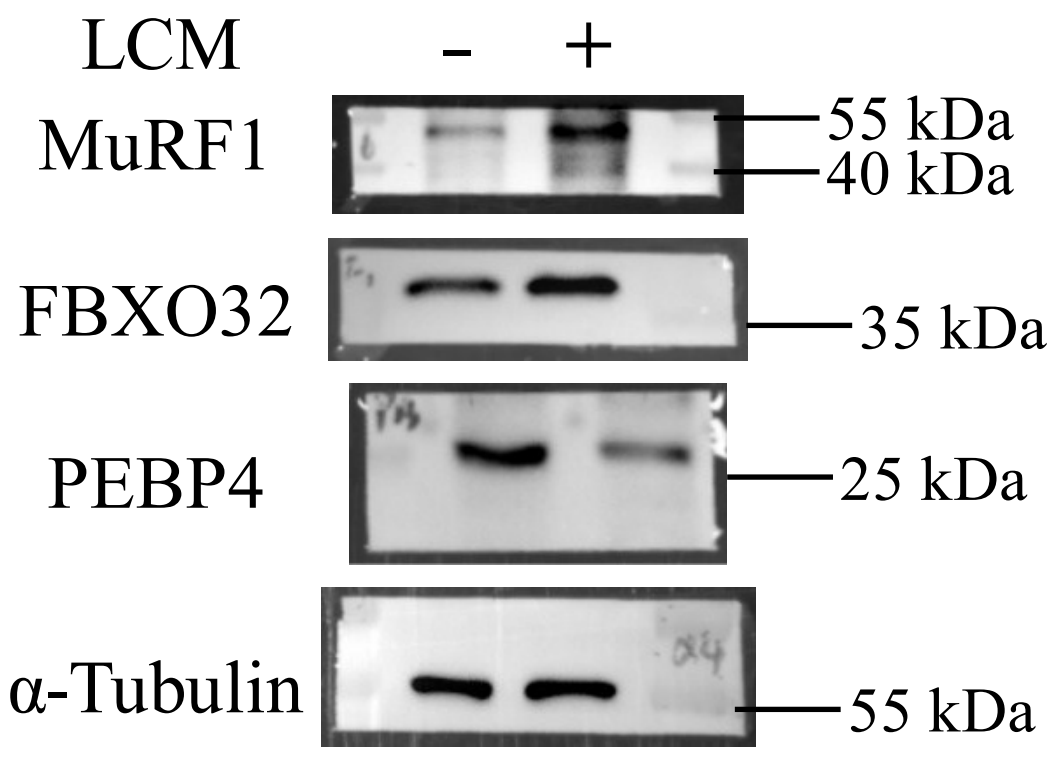

Figure 1

Figure 1F

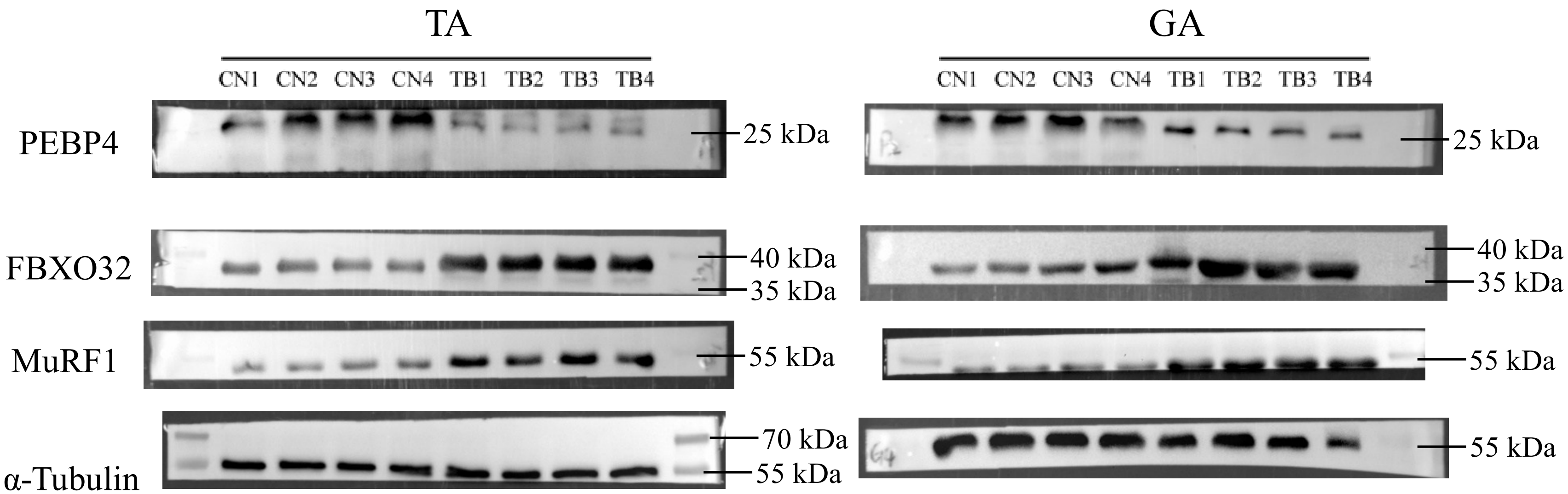

Figure 1

Figure 1I

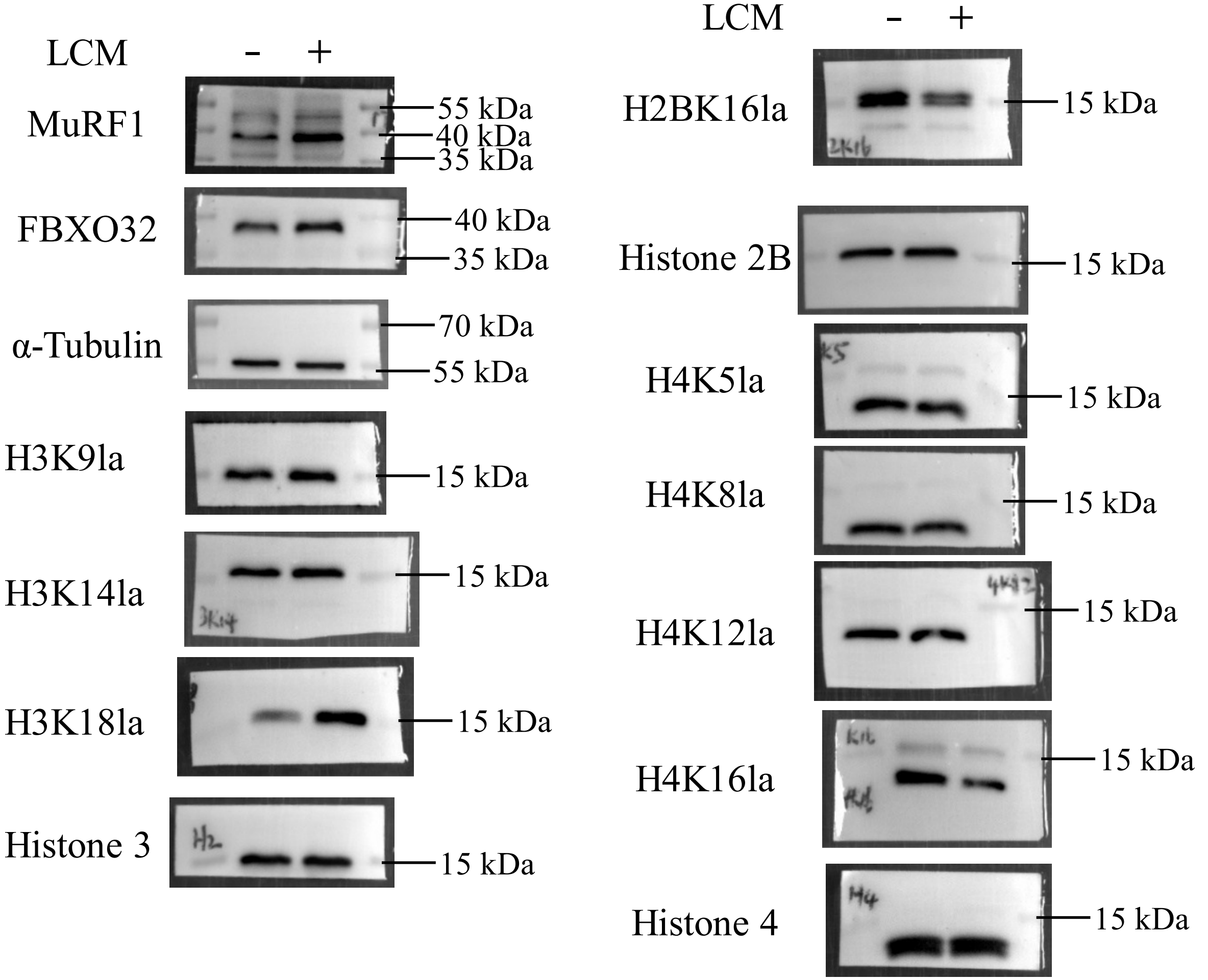

Figure 1

Figure 1J

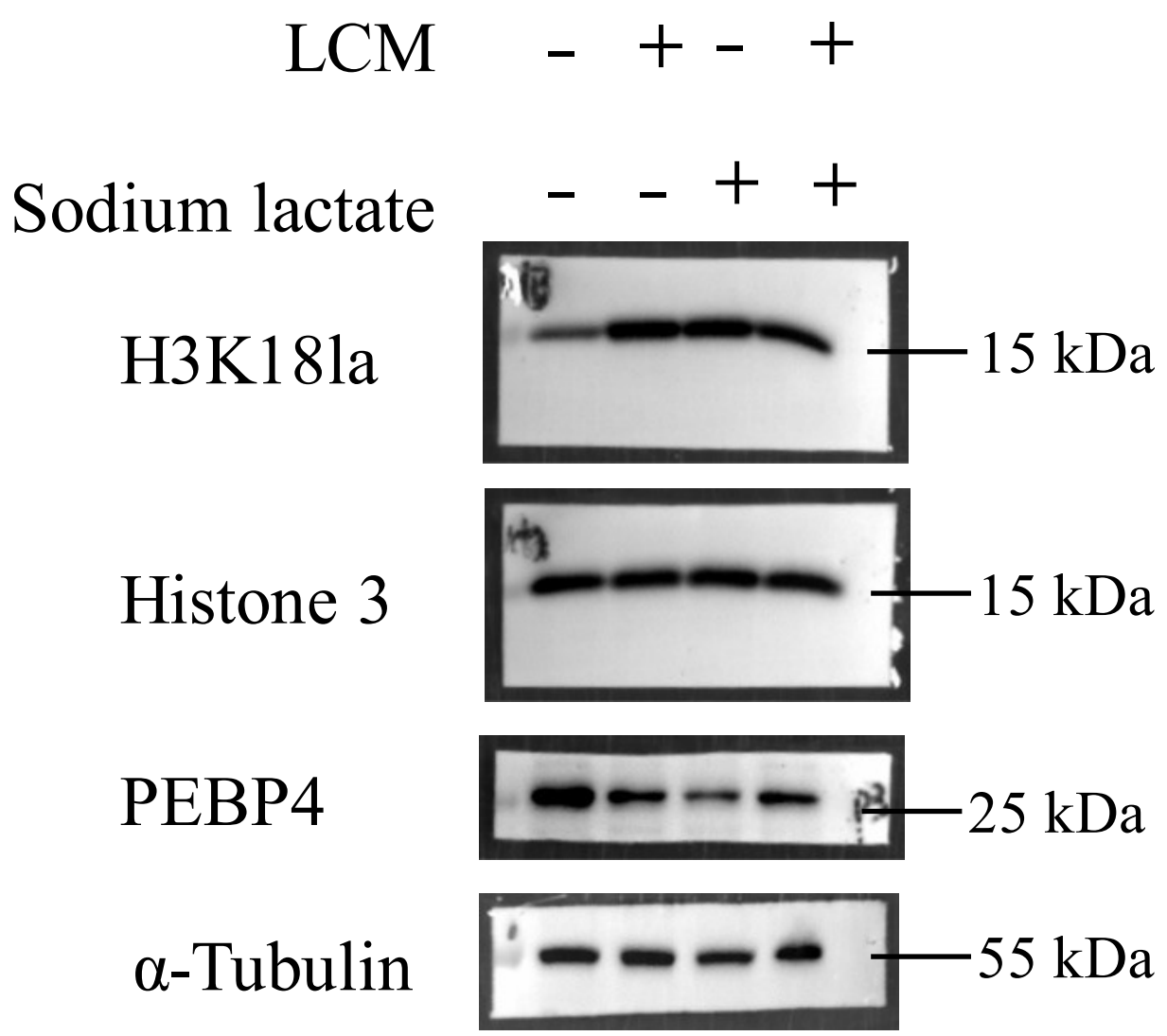

Figure 1

Figure 1K

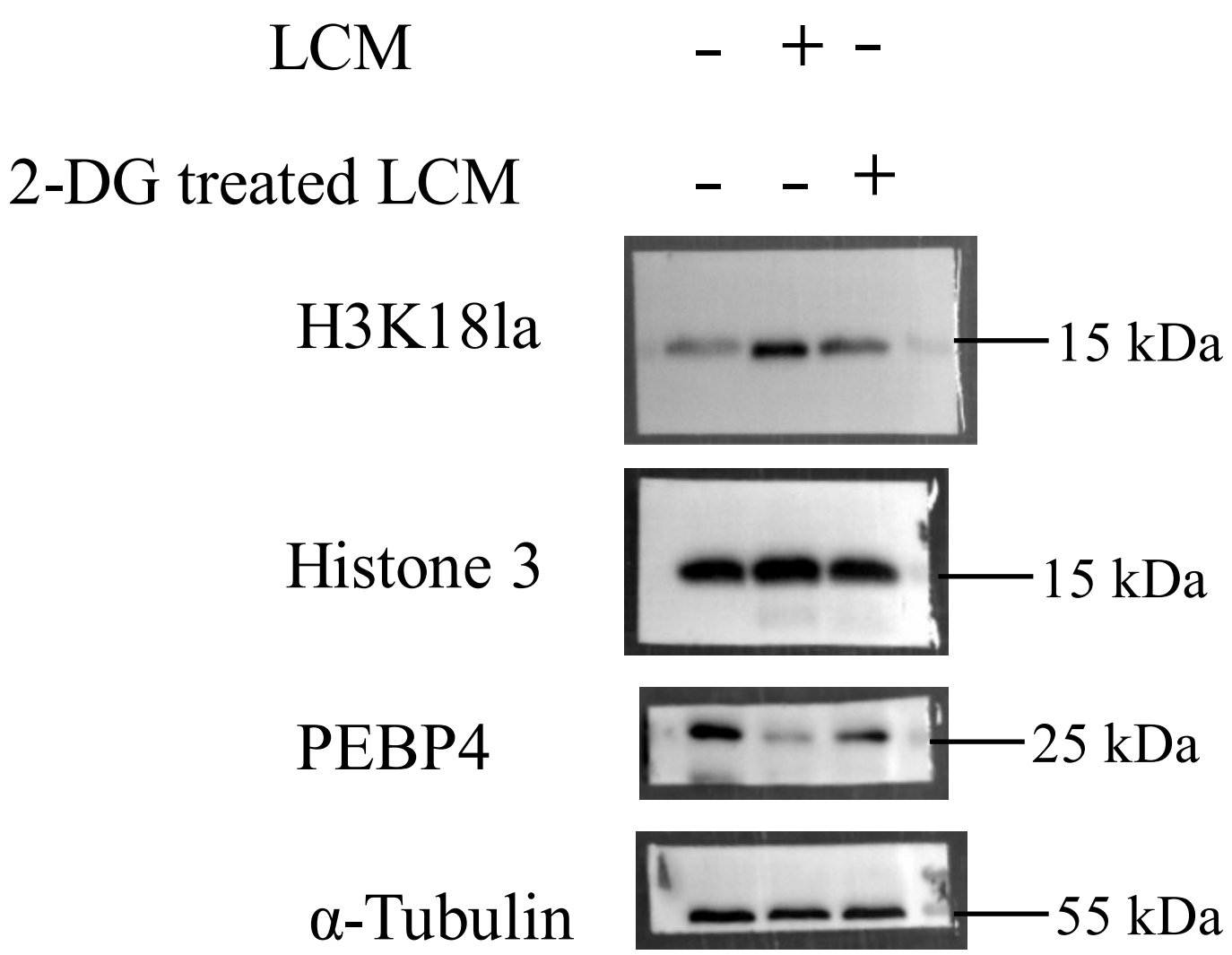

Figure 1

Figure 1L

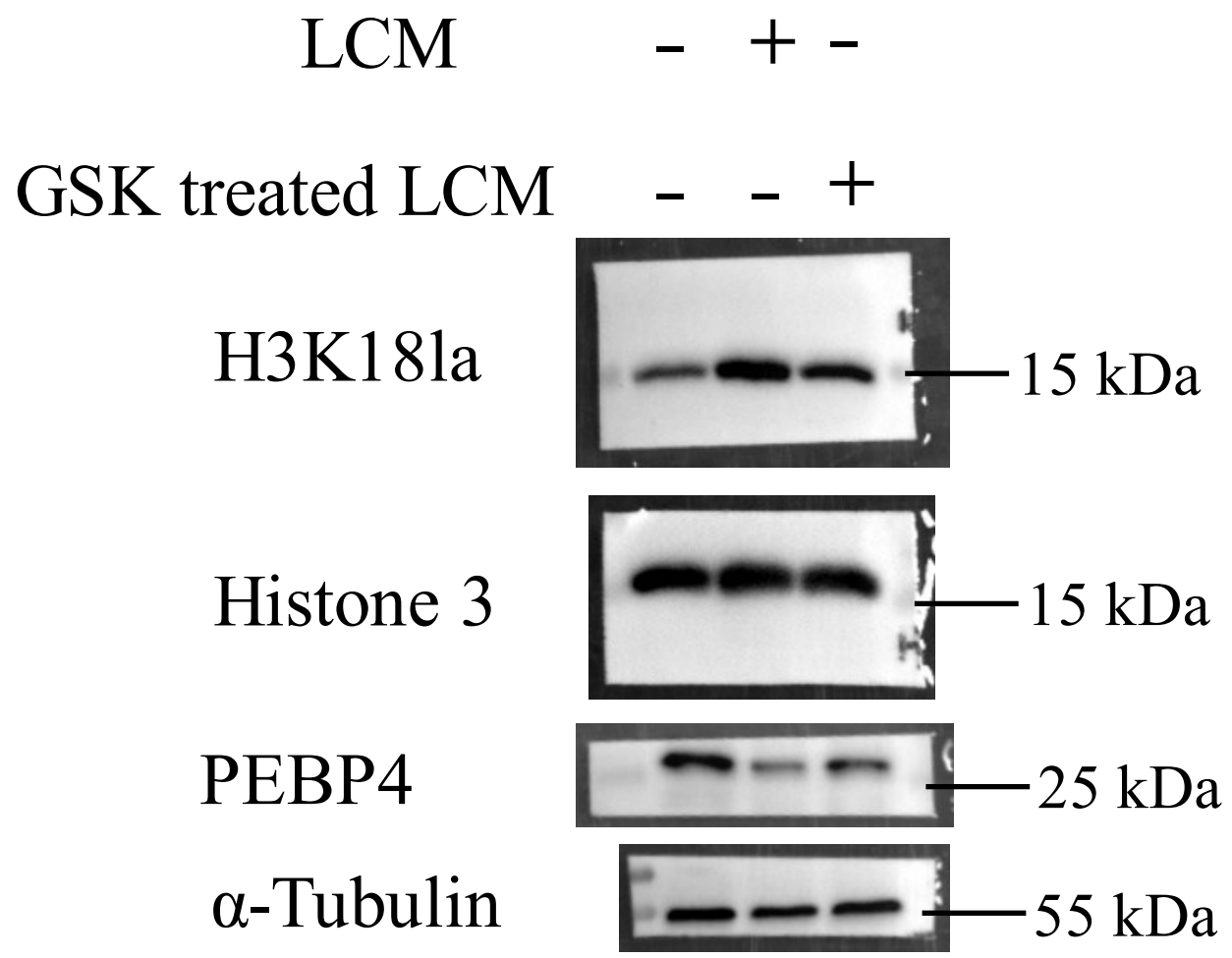

Figure 2

Figure 2A

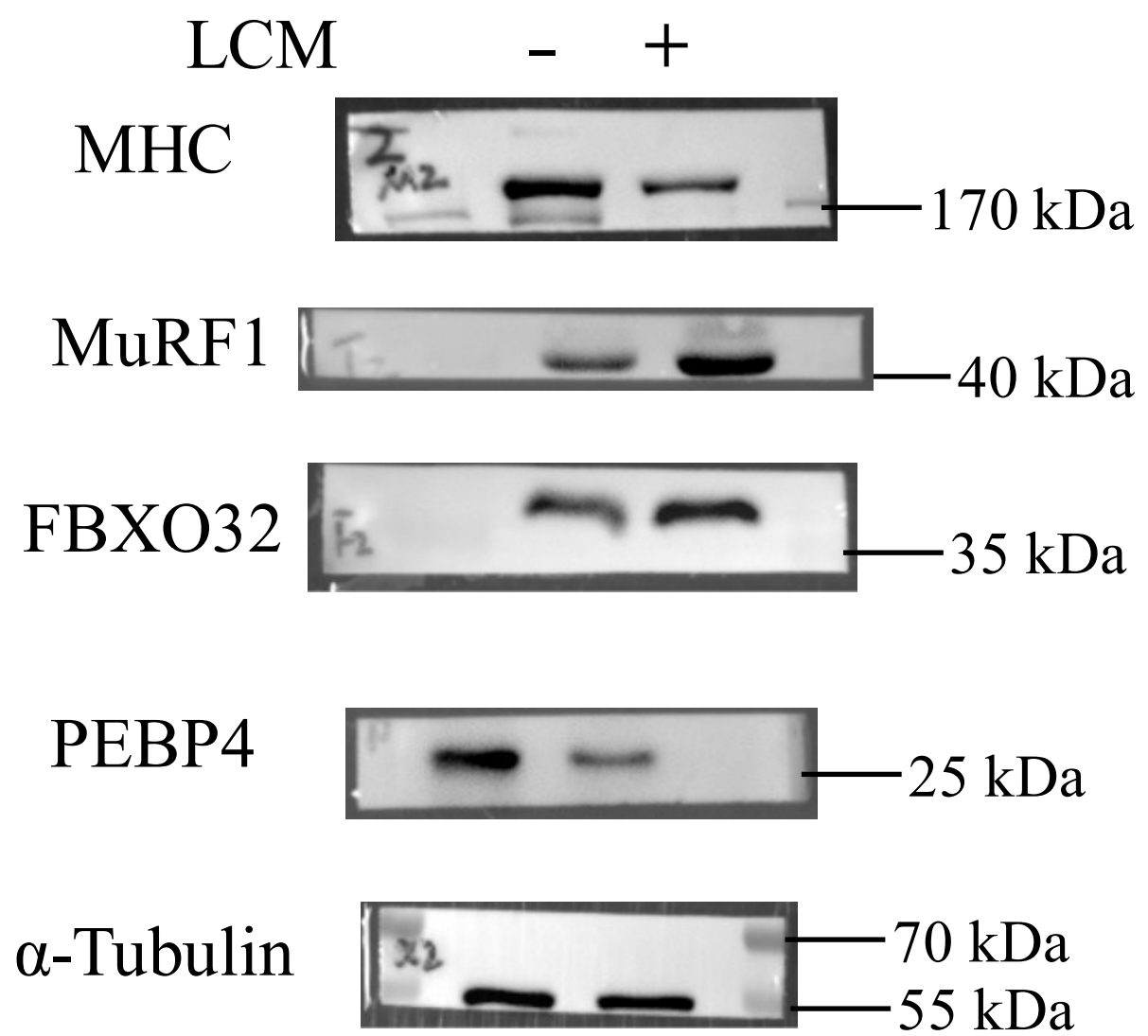

Figure 2

Figure 2C

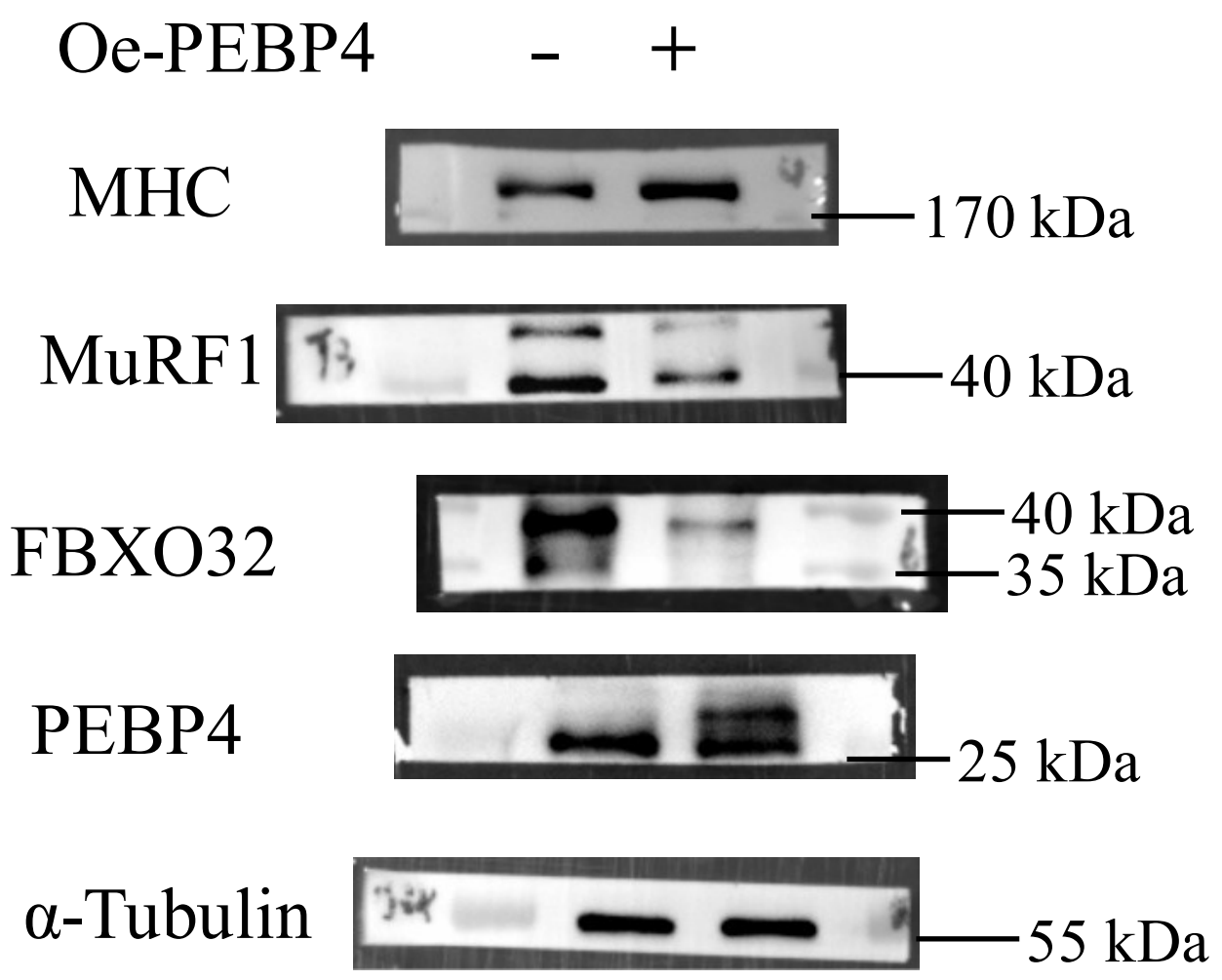

Figure 2

Figure 2L

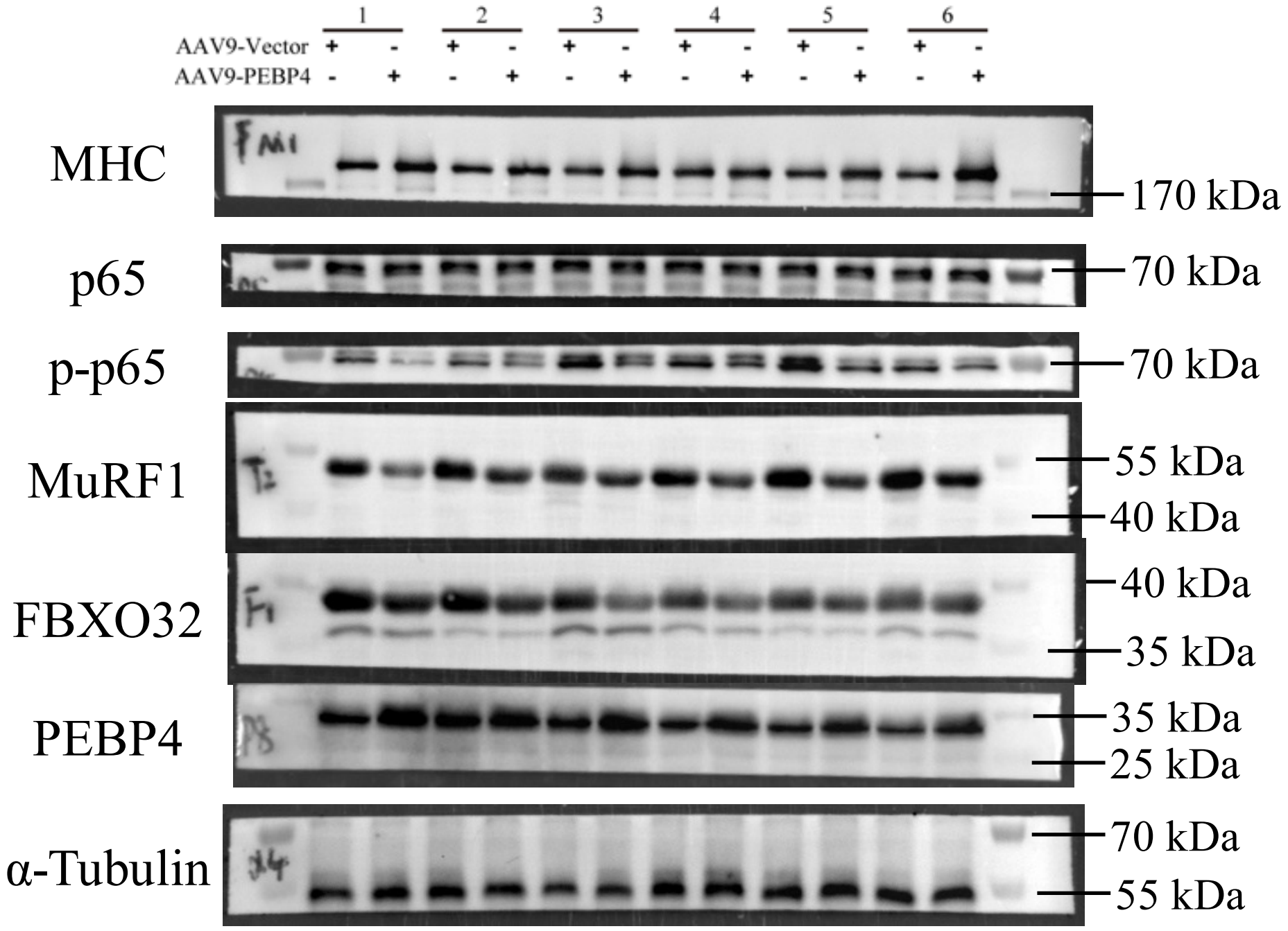

Figure 3

Figure 3F

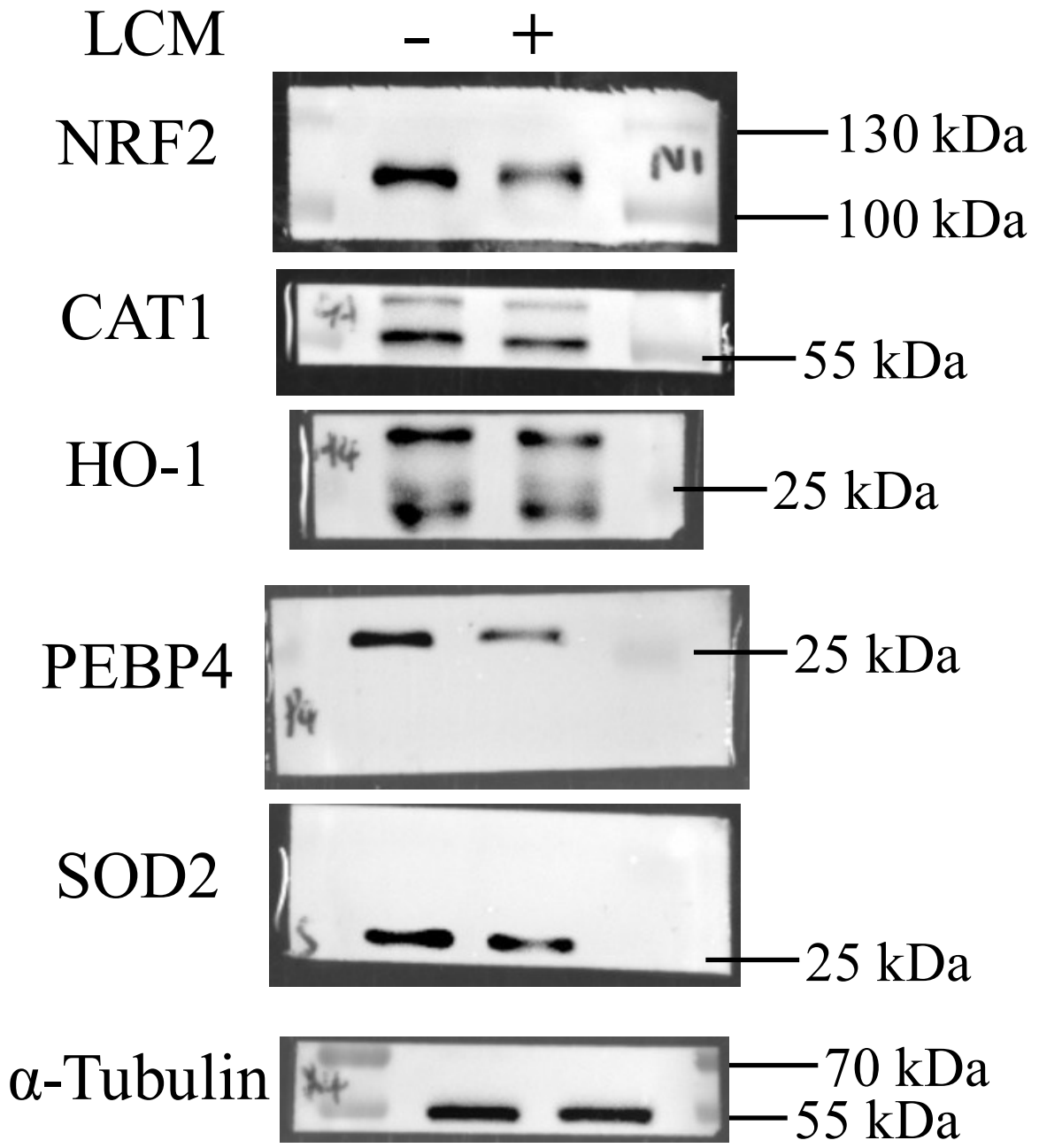

Figure 3

Figure 3I

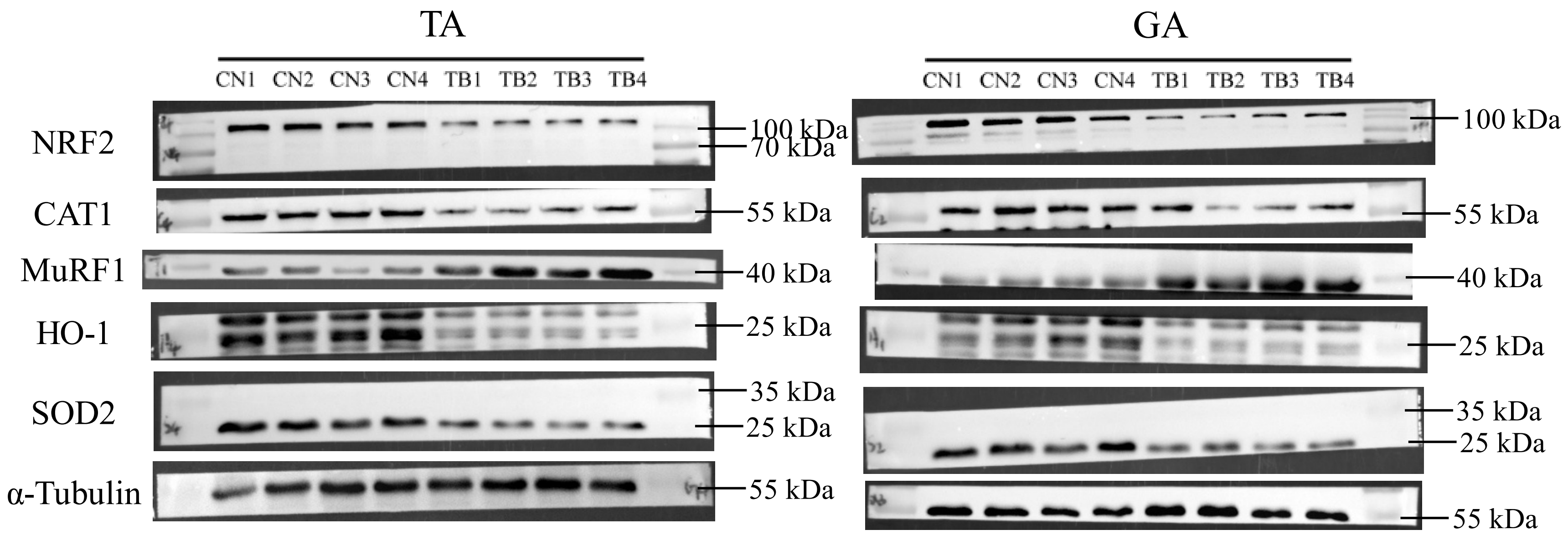

Figure 3

Figure 3M

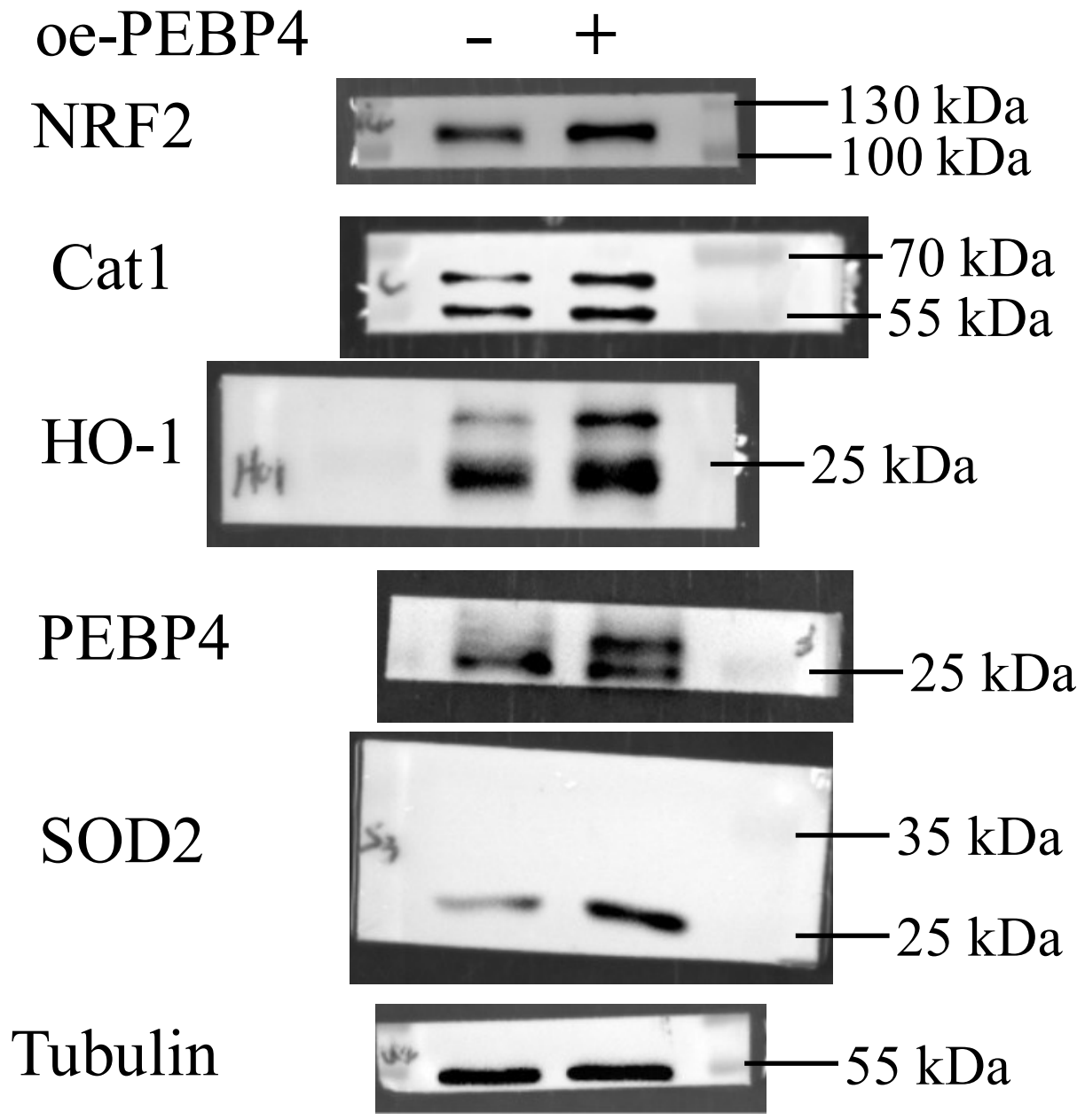

Figure 4

Figure 4C

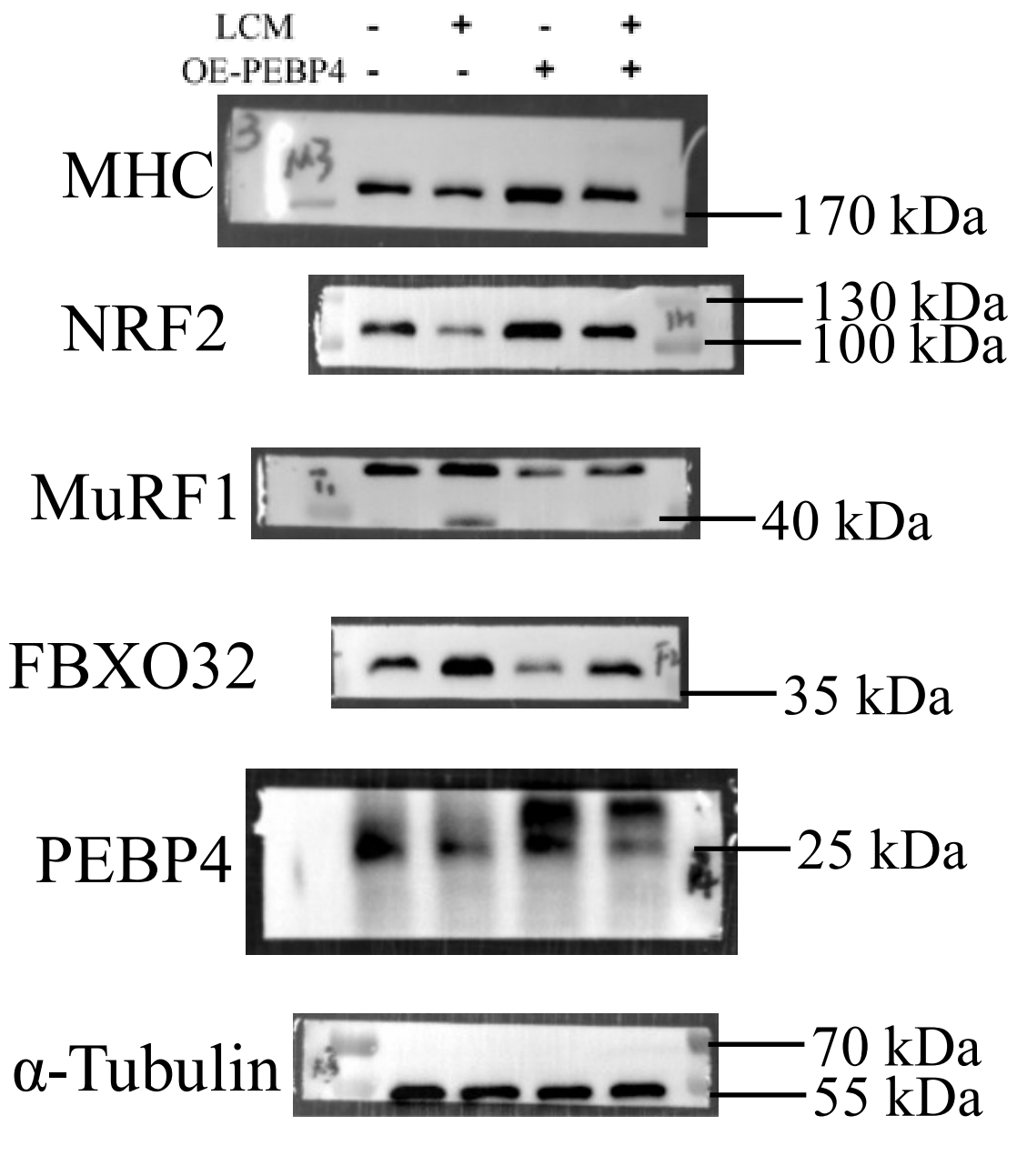

Figure 4

Figure 4G

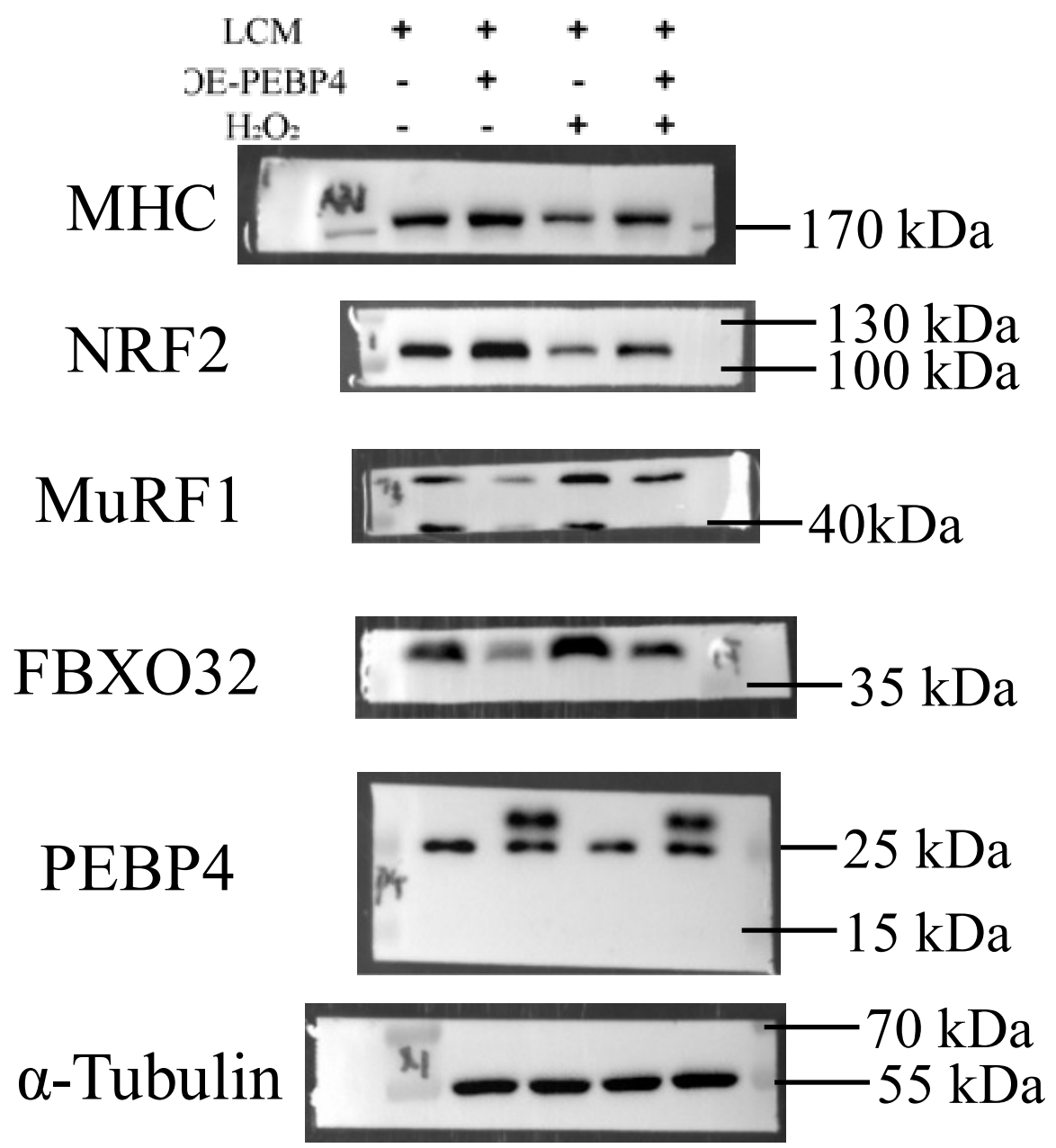

Figure 4

Figure 4K

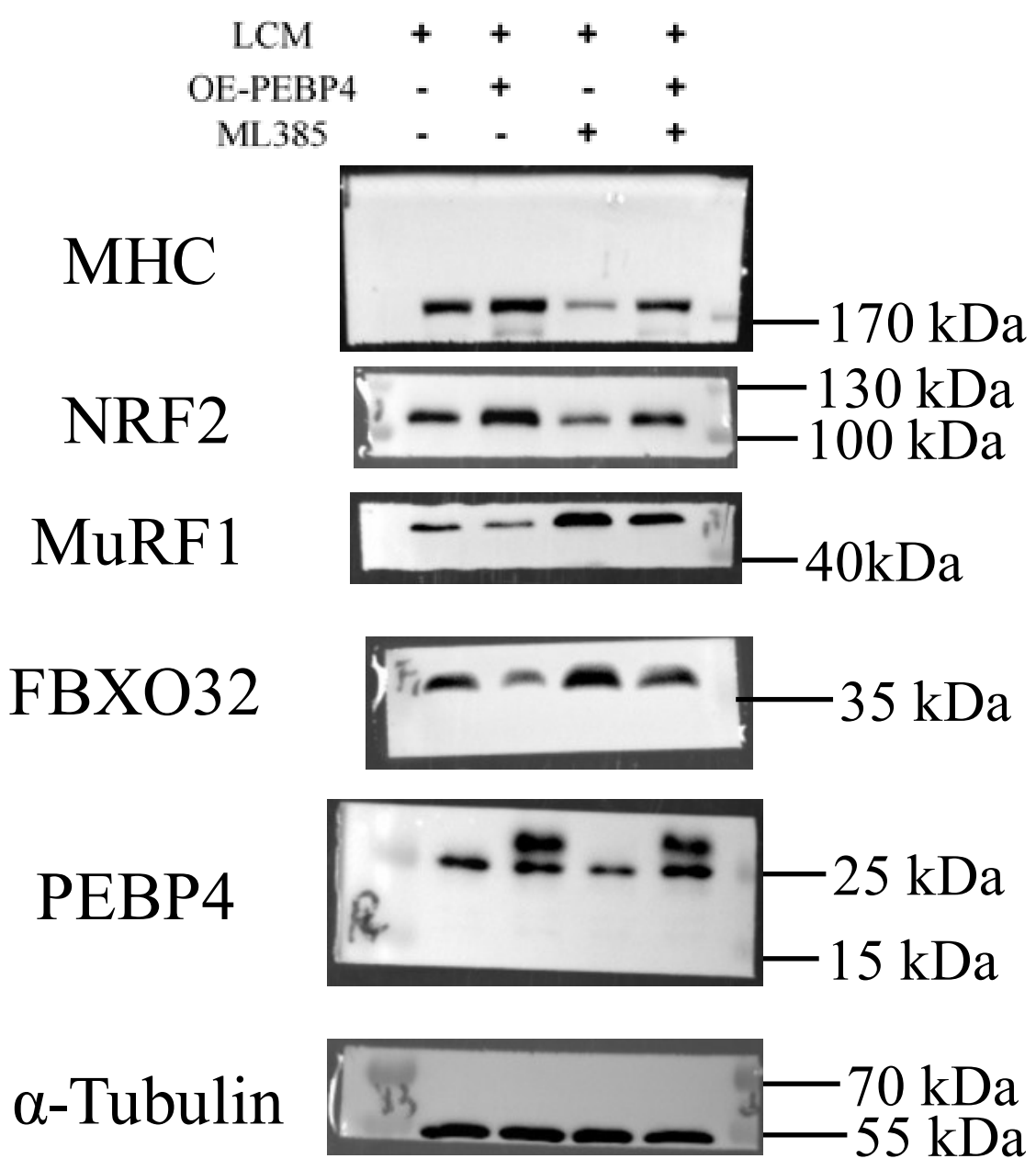

Figure 5

Figure 5B

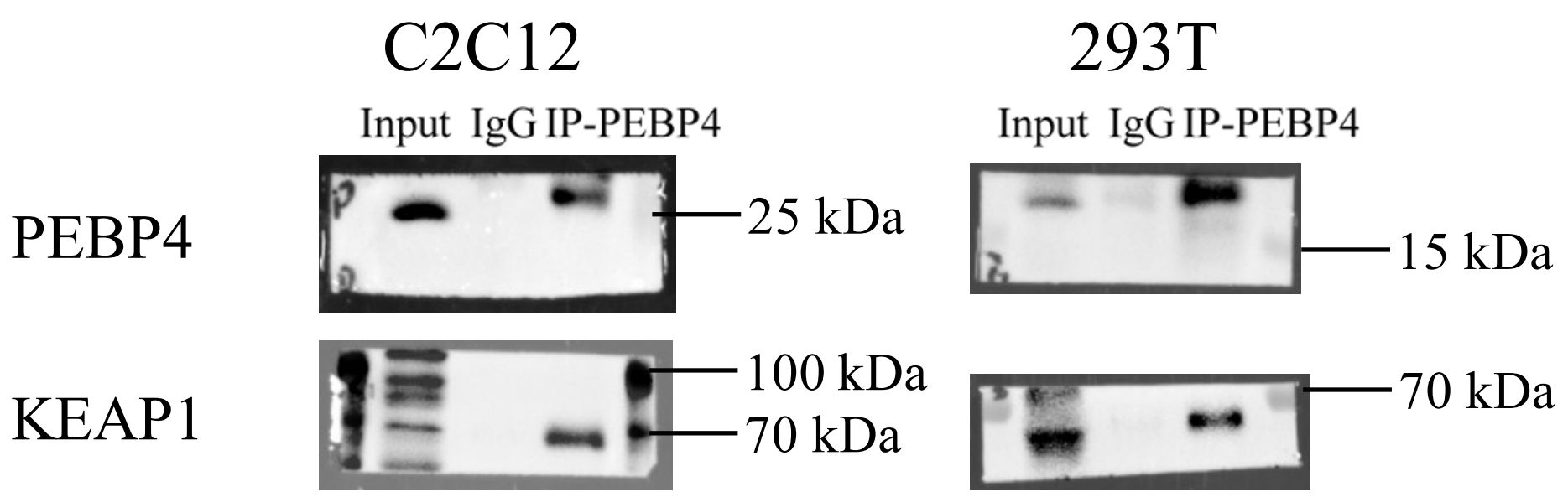

Figure 5

Figure 5D

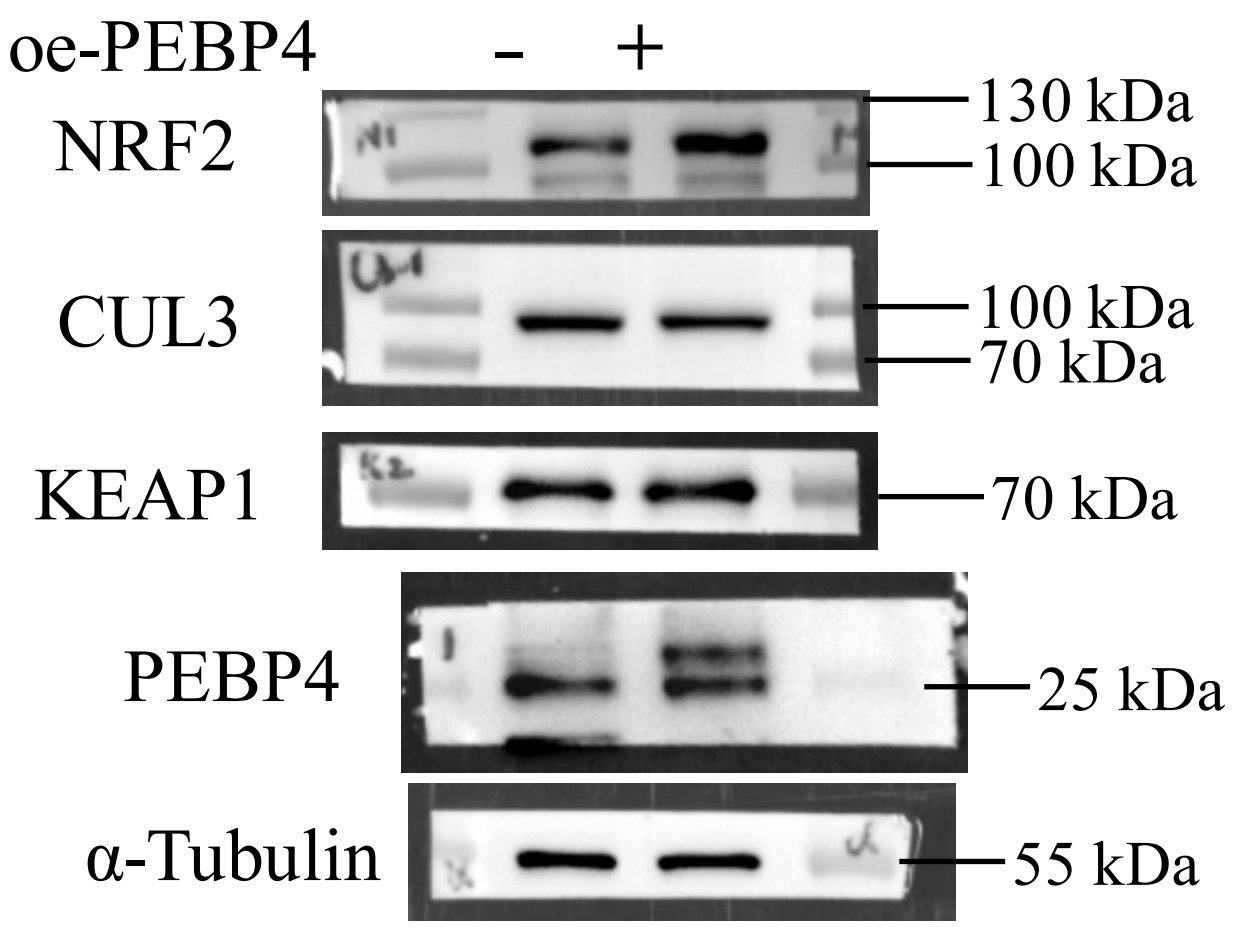

Figure 5

Figure 5E

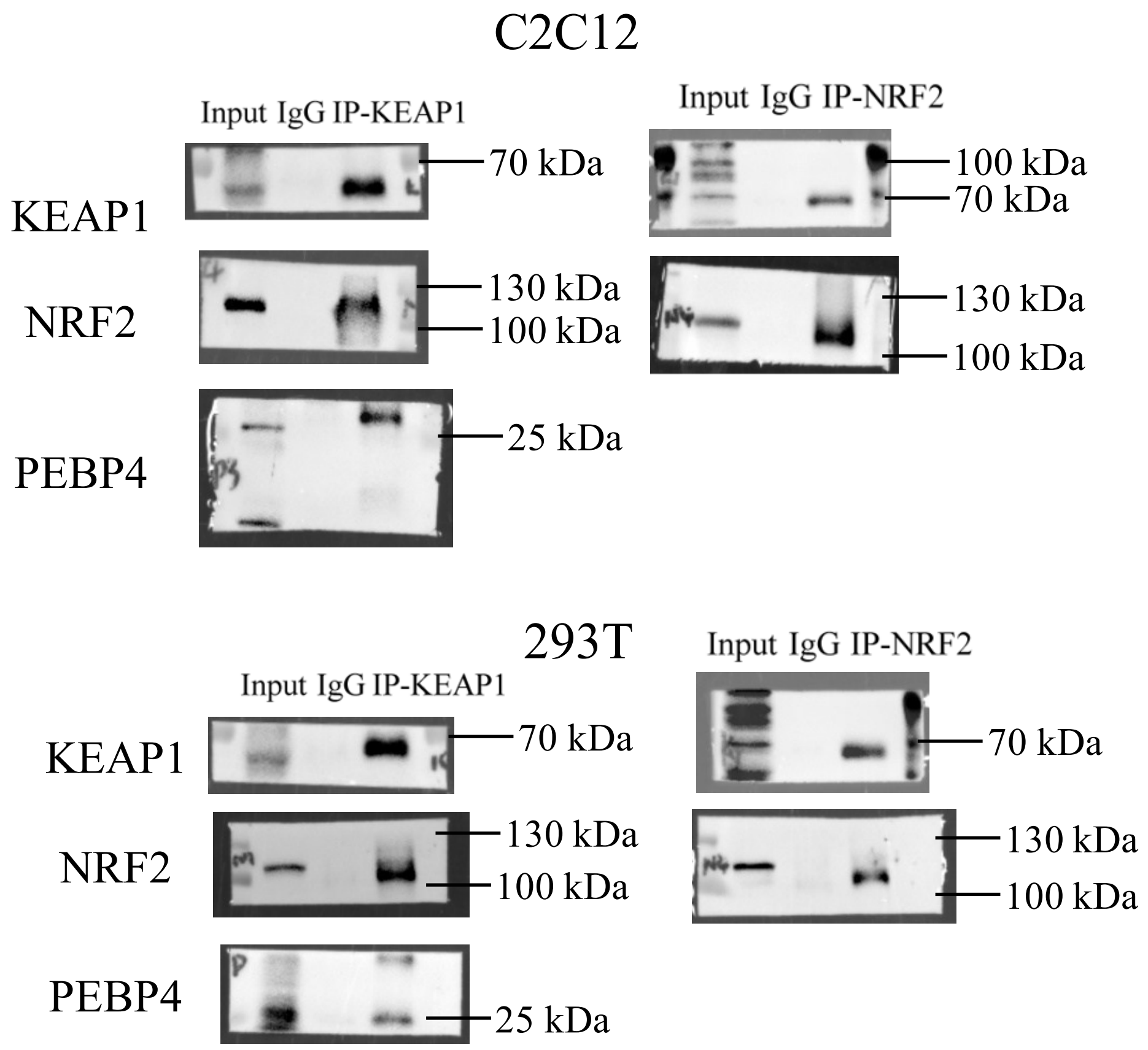

Figure 5

Figure 5H

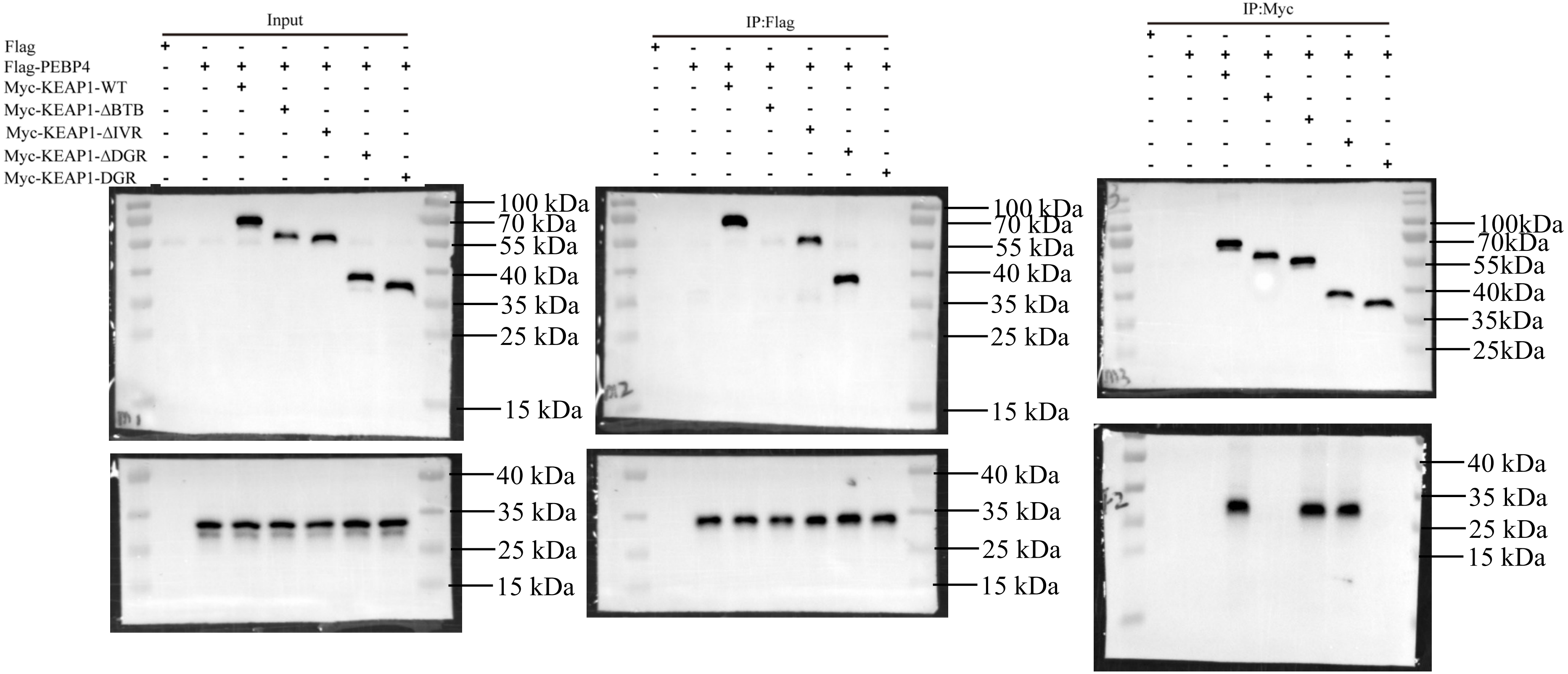

Figure 5

Figure 5I

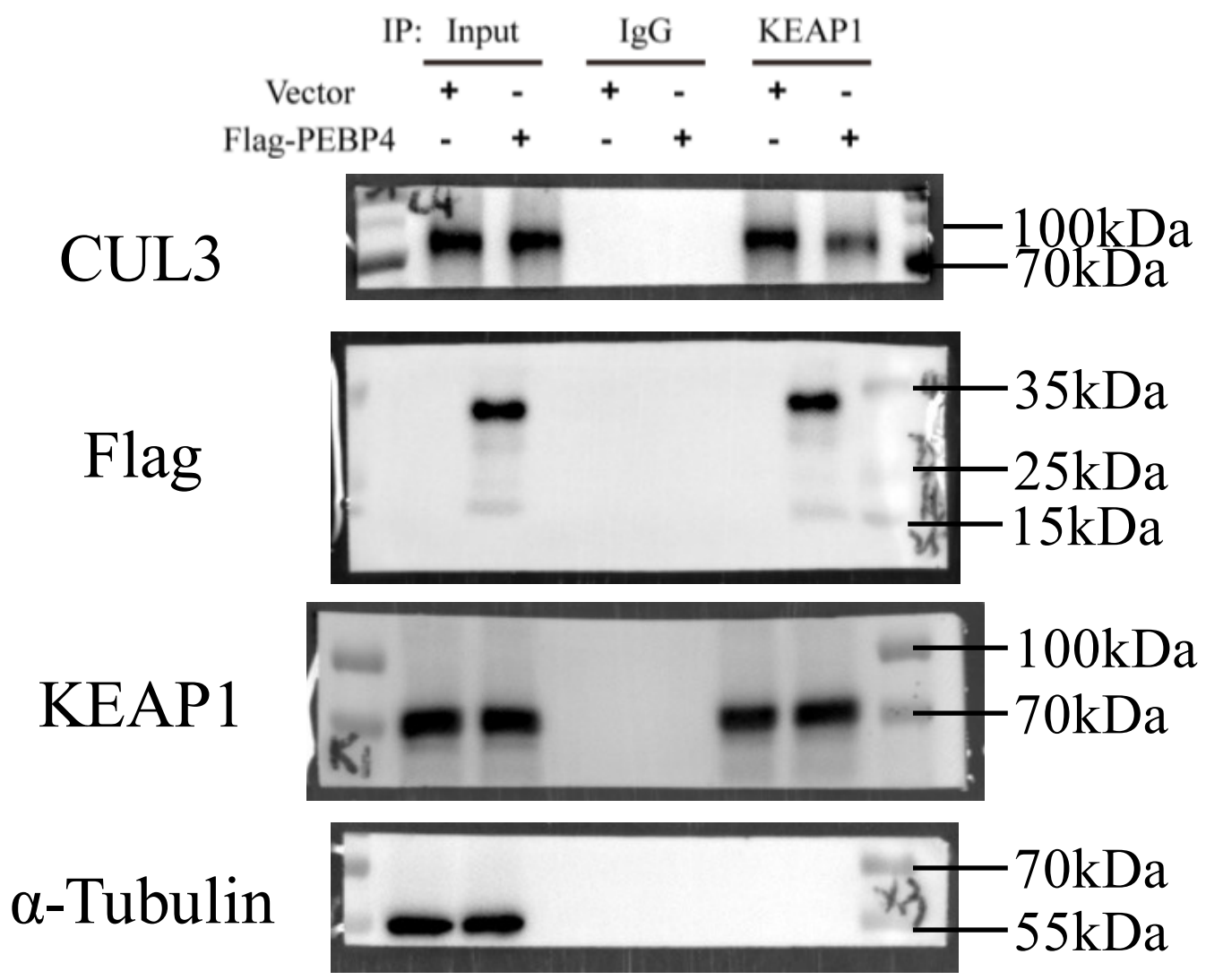

Figure 5

Figure 5J

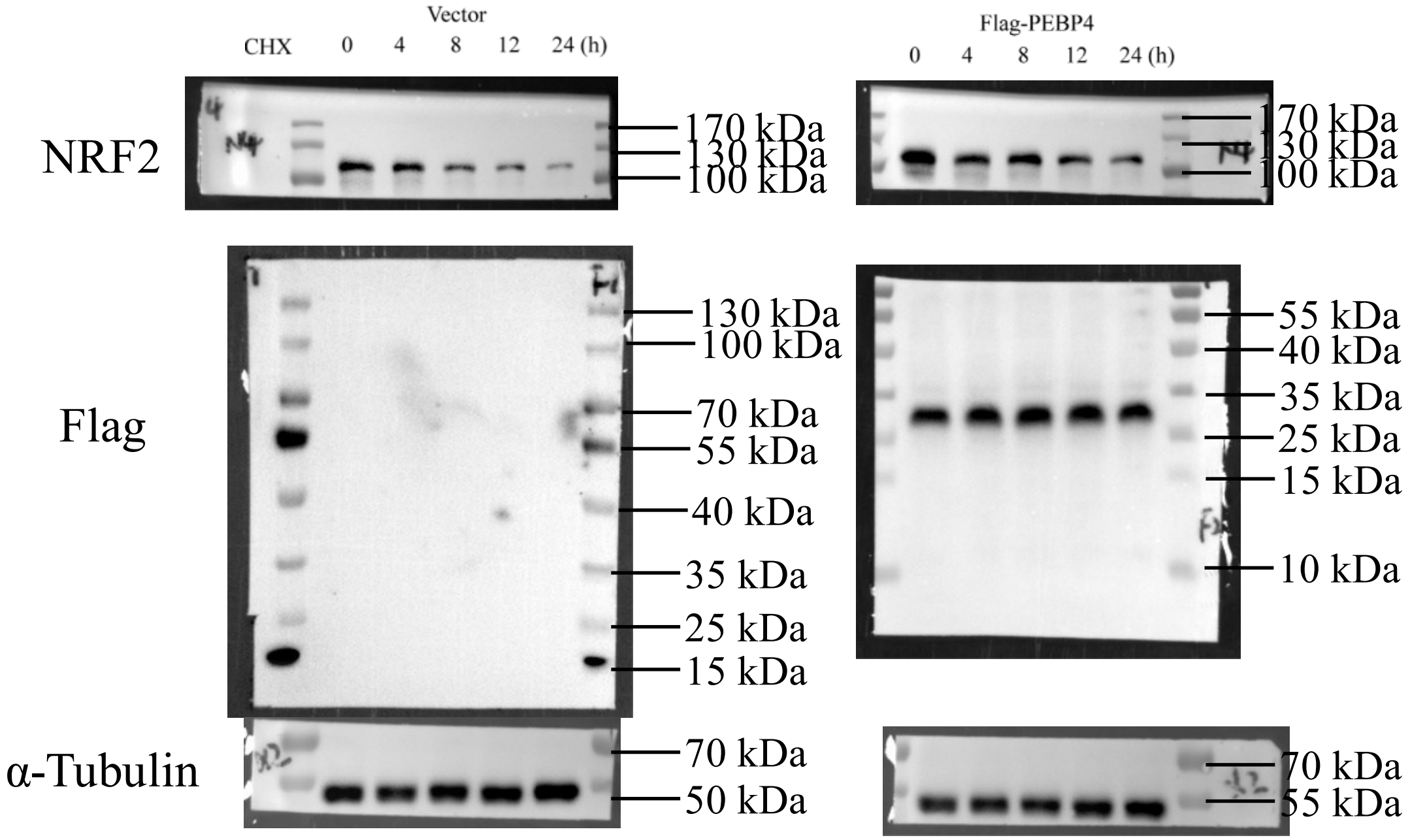

Figure 5

Figure 5L

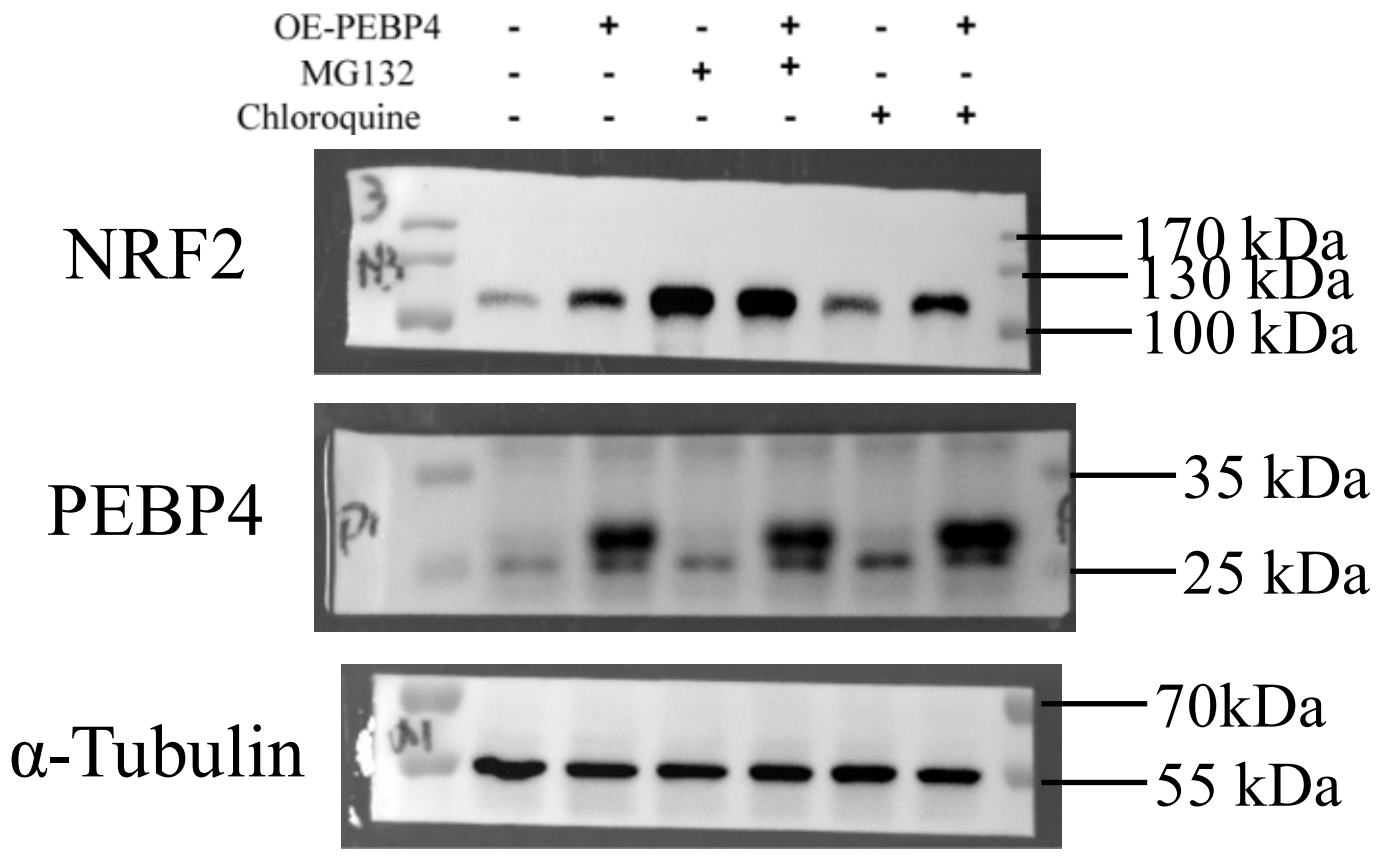

Figure 5

Figure 5M

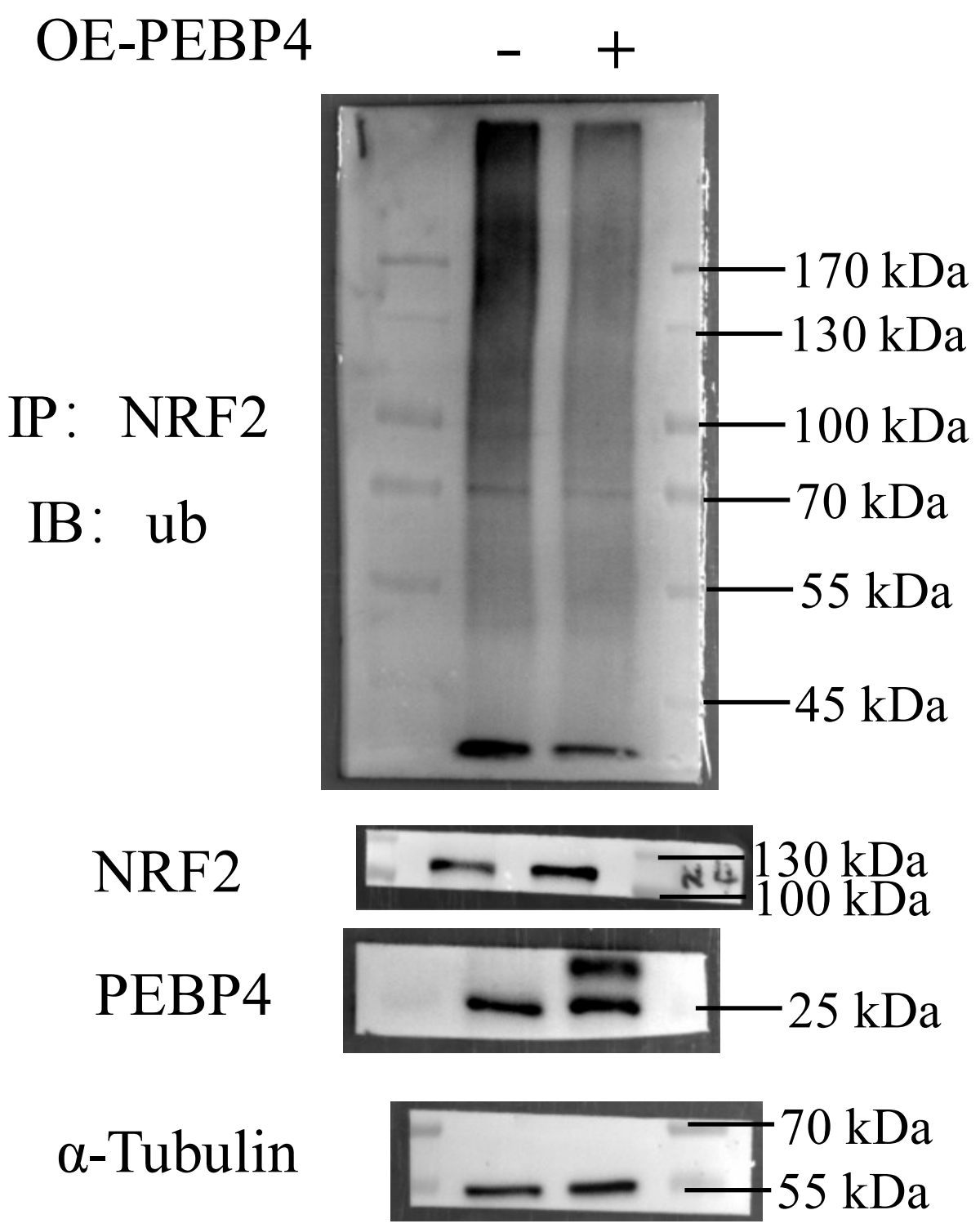

Figure 5

Figure 5N

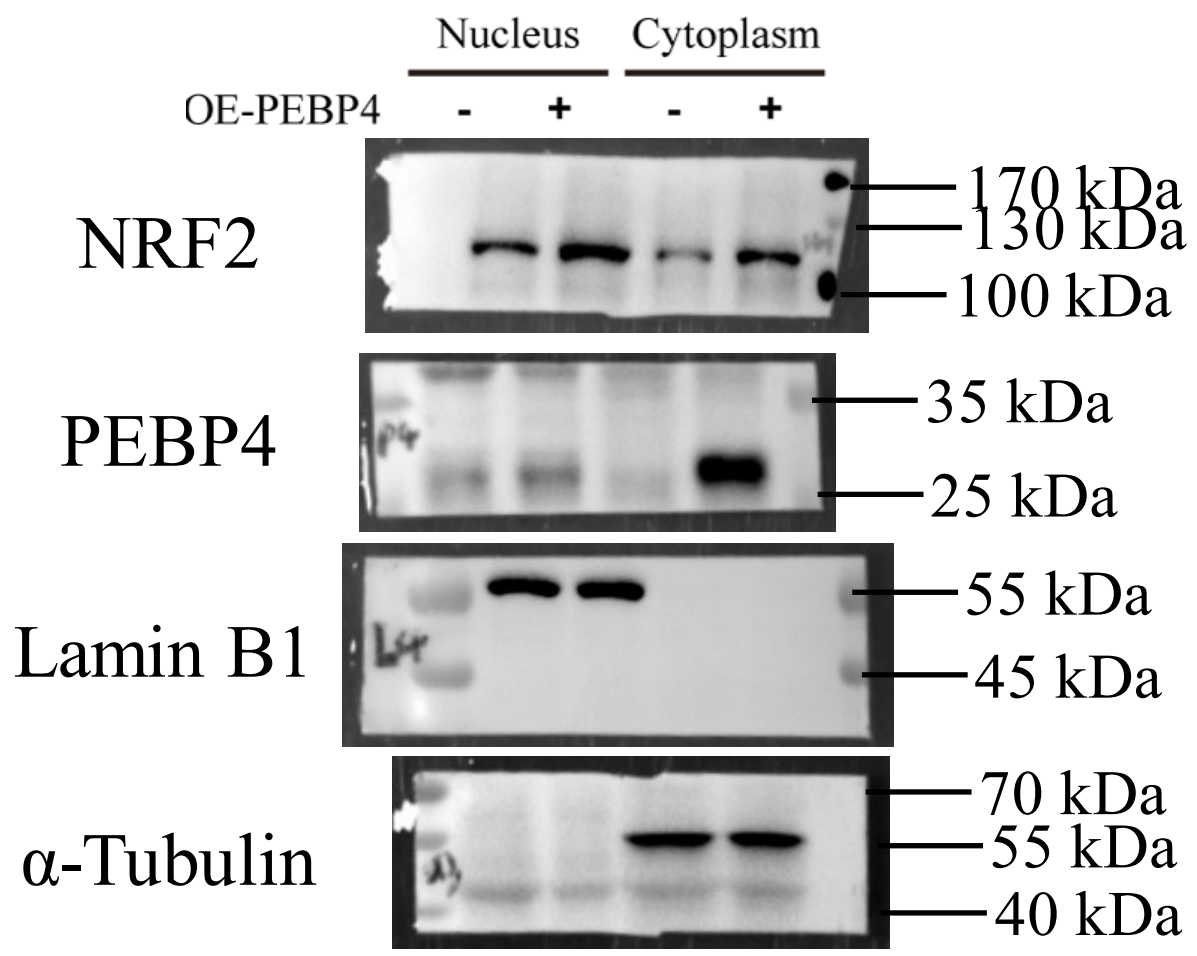

Figure 6

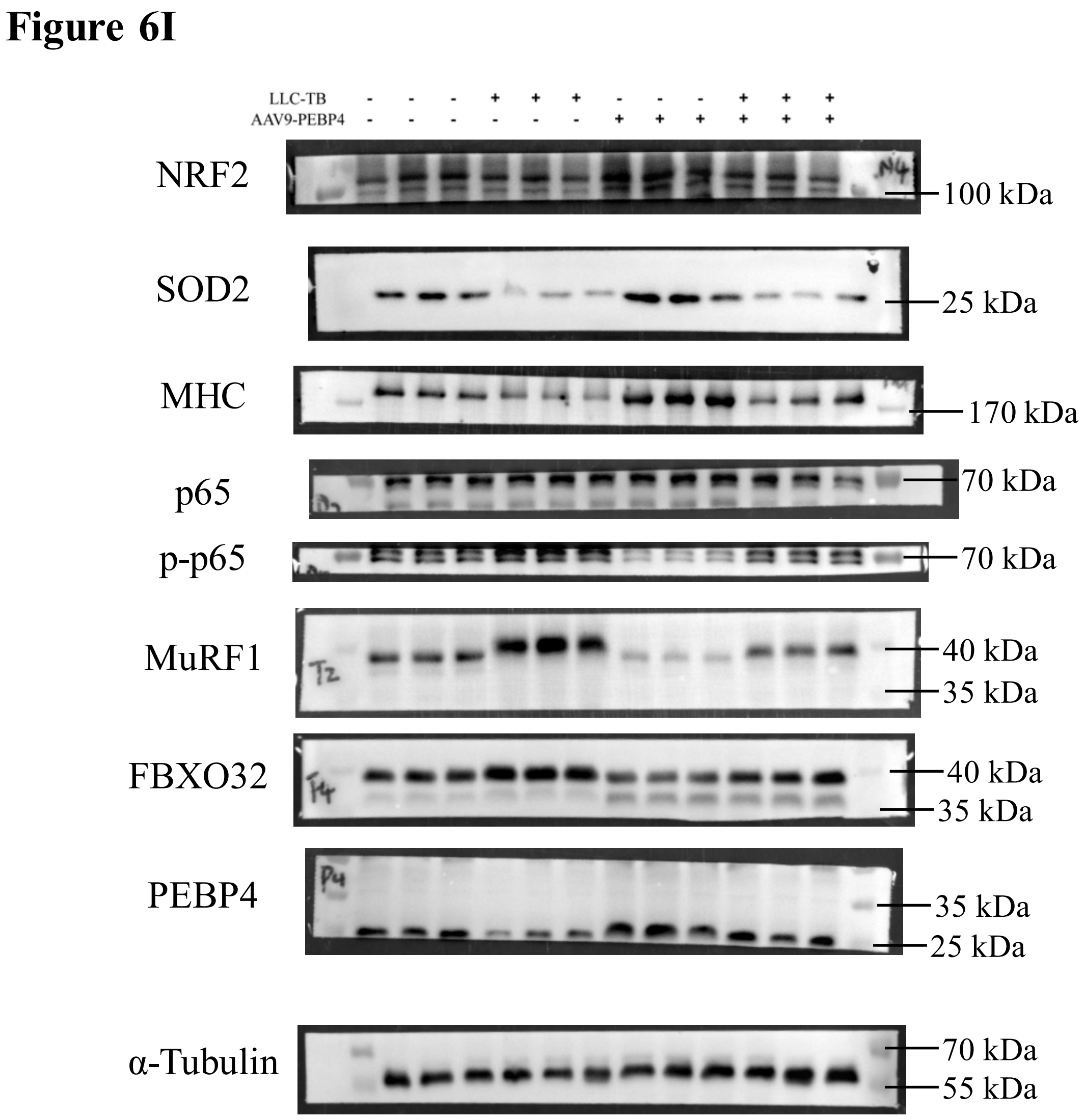
Figure 6I
LLC-TB - - - + + + - - - + + +
AAV9-PEBP4 - - - - - - + + + + + +
NRF2
100 kDa
SOD2
25 kDa
MHC
170 kDa
p65
70 kDa
p-p65
70 kDa
MuRF1
40 kDa
35 kDa
FBXO32
40 kDa
35 kDa
PEBP4
35 kDa
25 kDa
α-Tubulin
70 kDa
55 kDa

Figure 7

Figure 7I

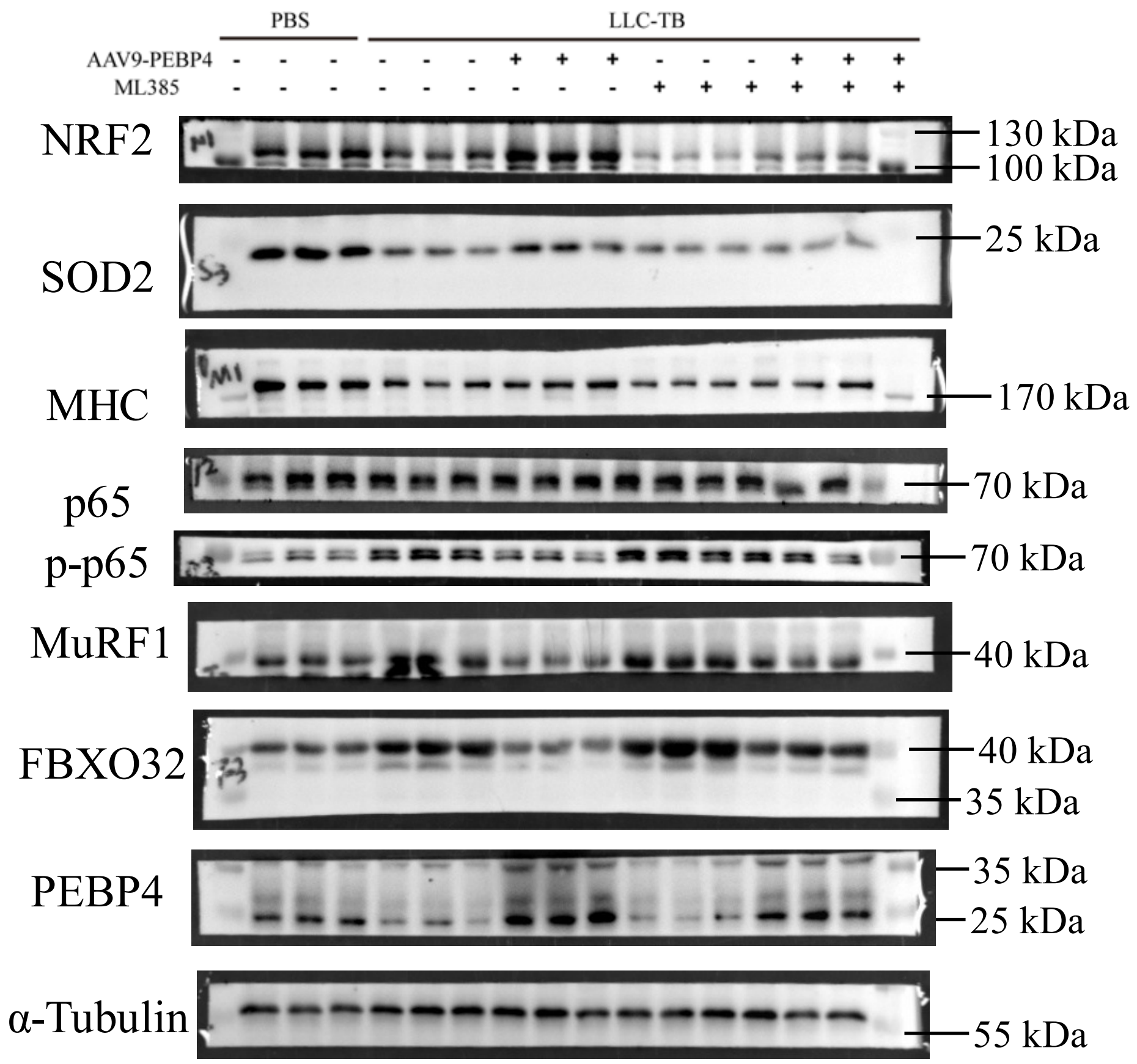

Figure S1

Figure S1J

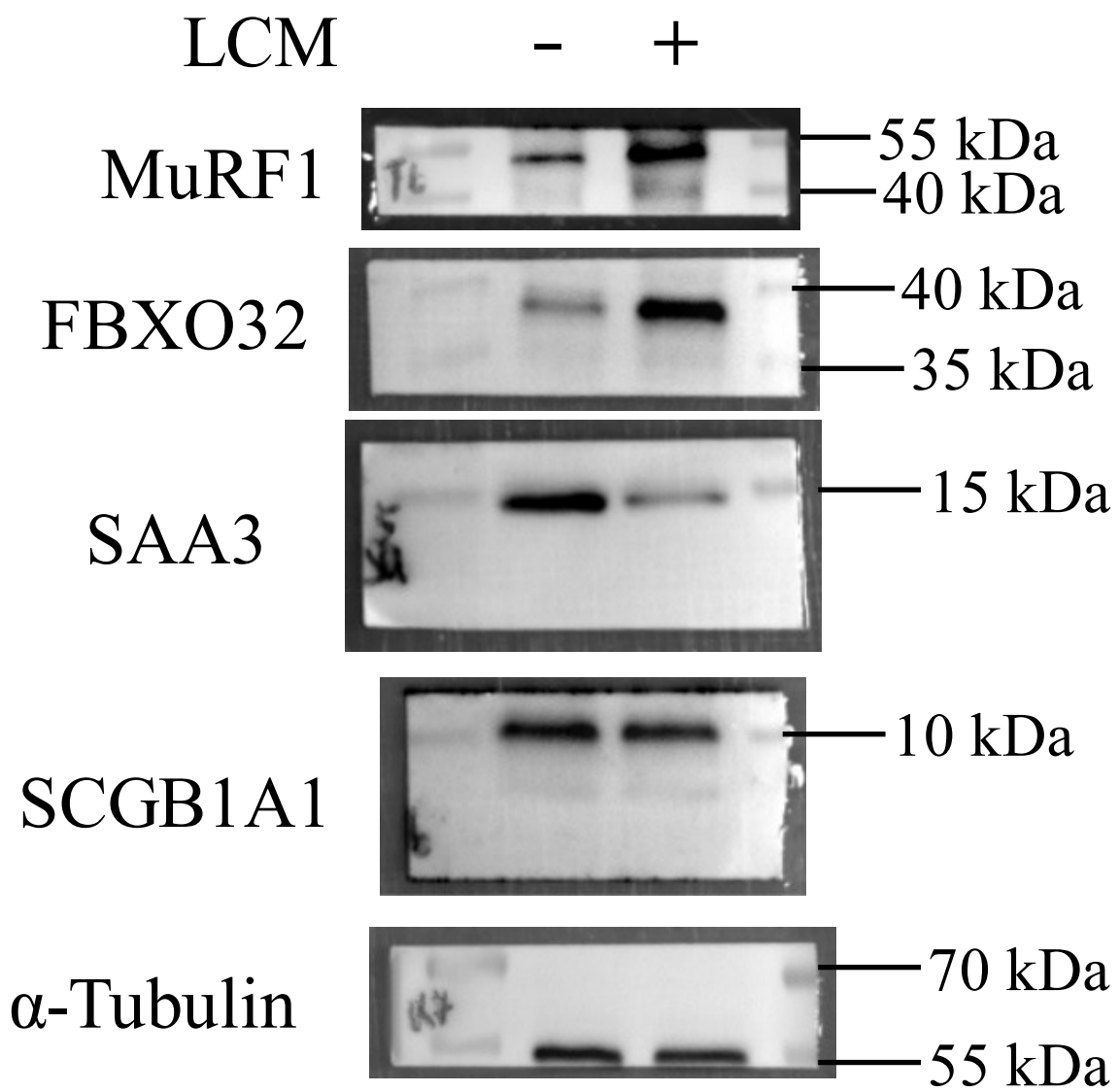

Figure S1

Figure S1O

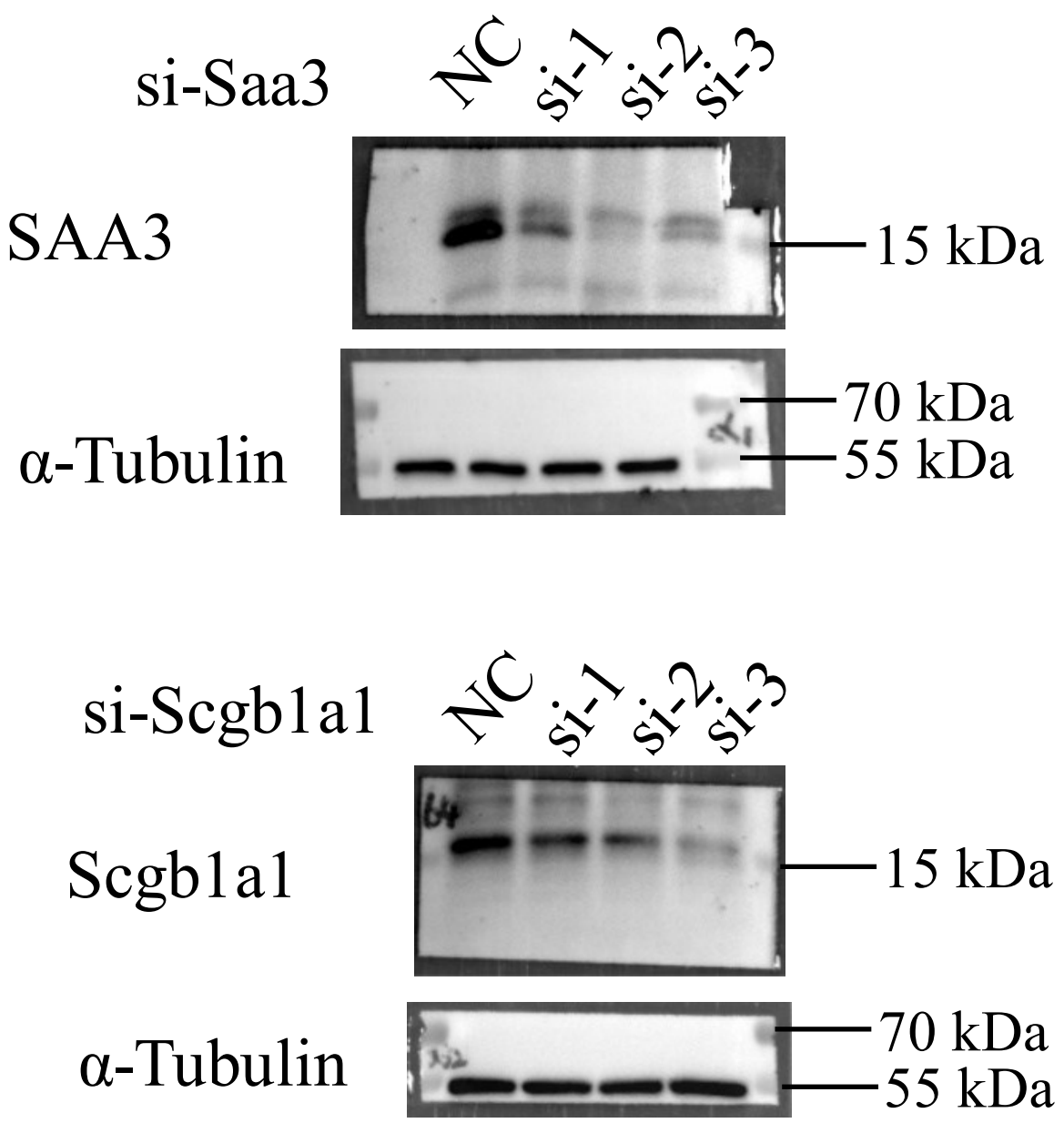

Figure S1

Figure S1P

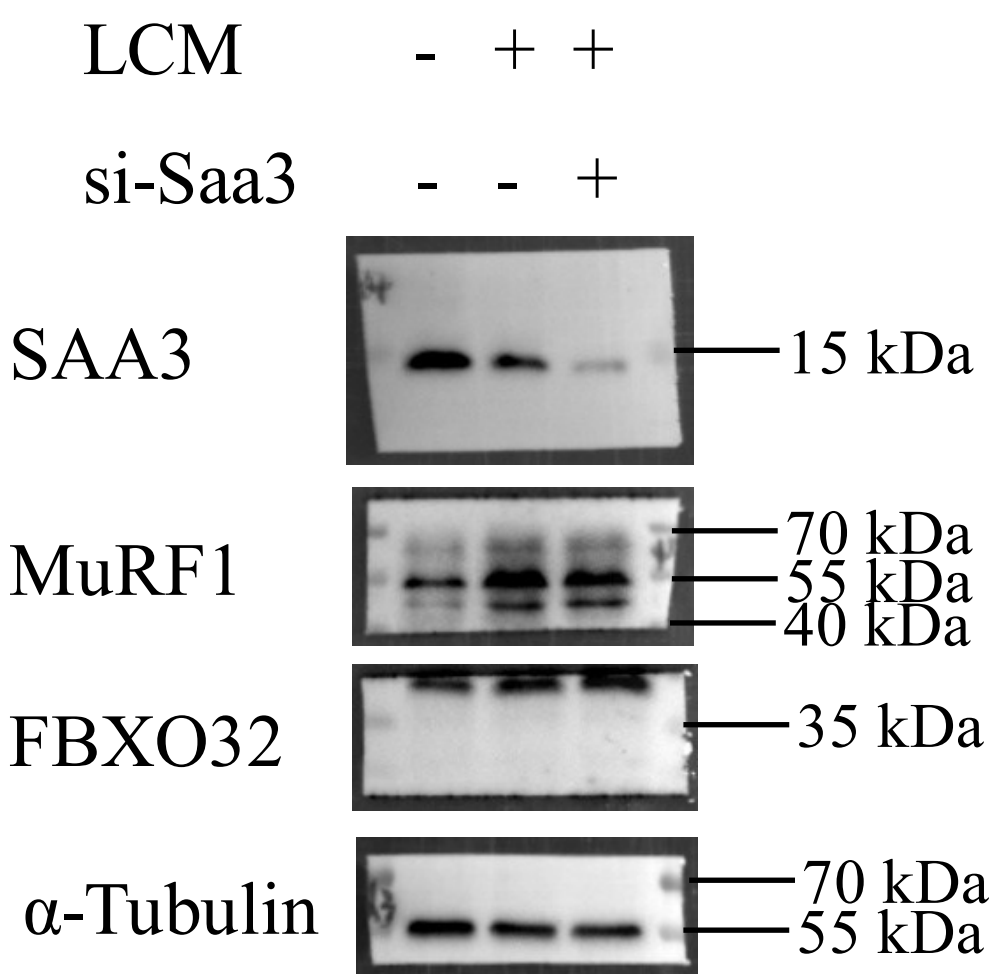

Figure S1

Figure S1Q

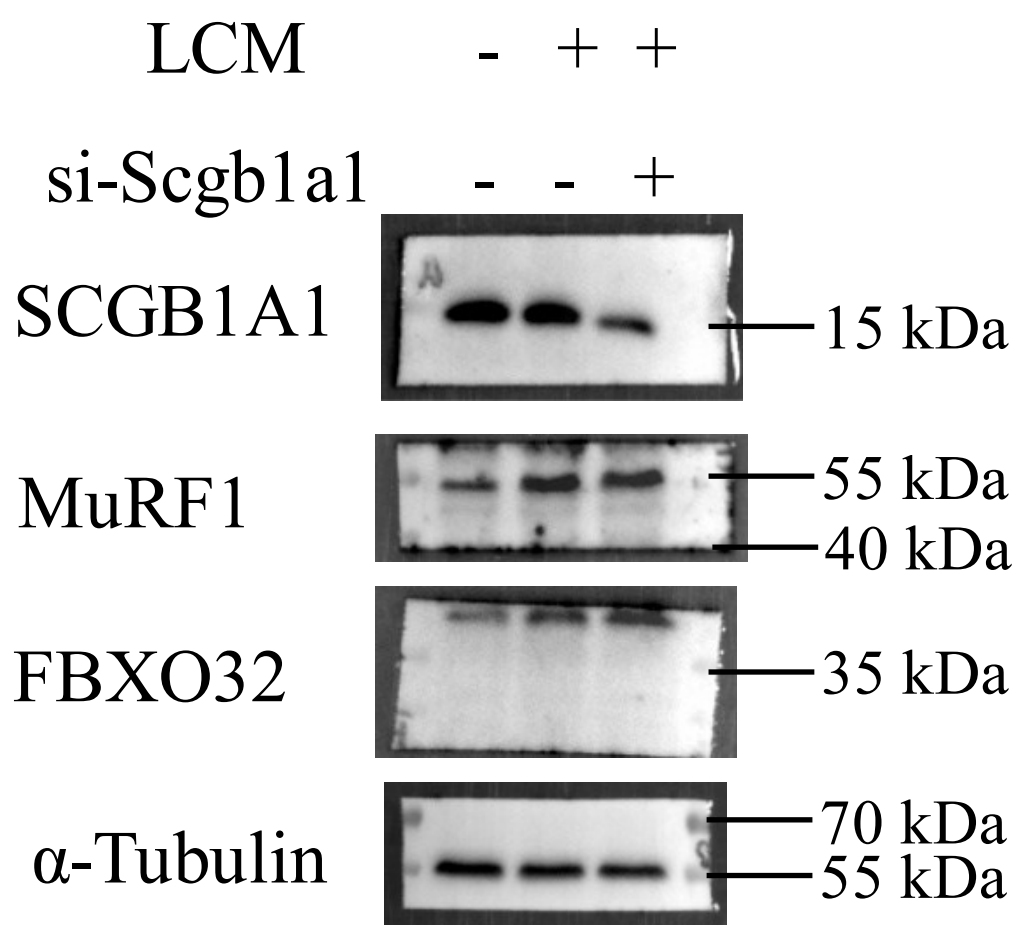

Figure S2

Figure S2B

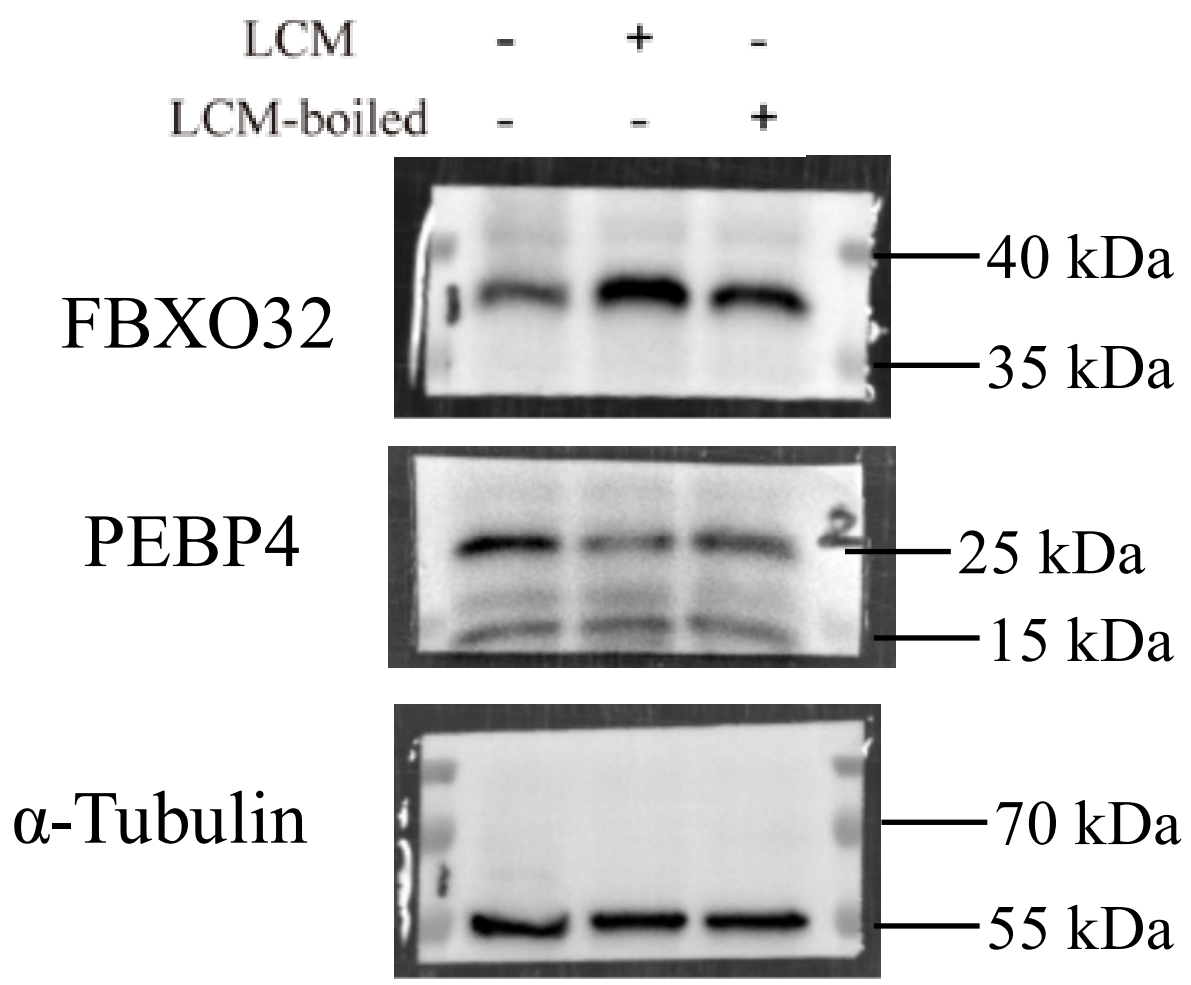

Figure S3

Figure S3C

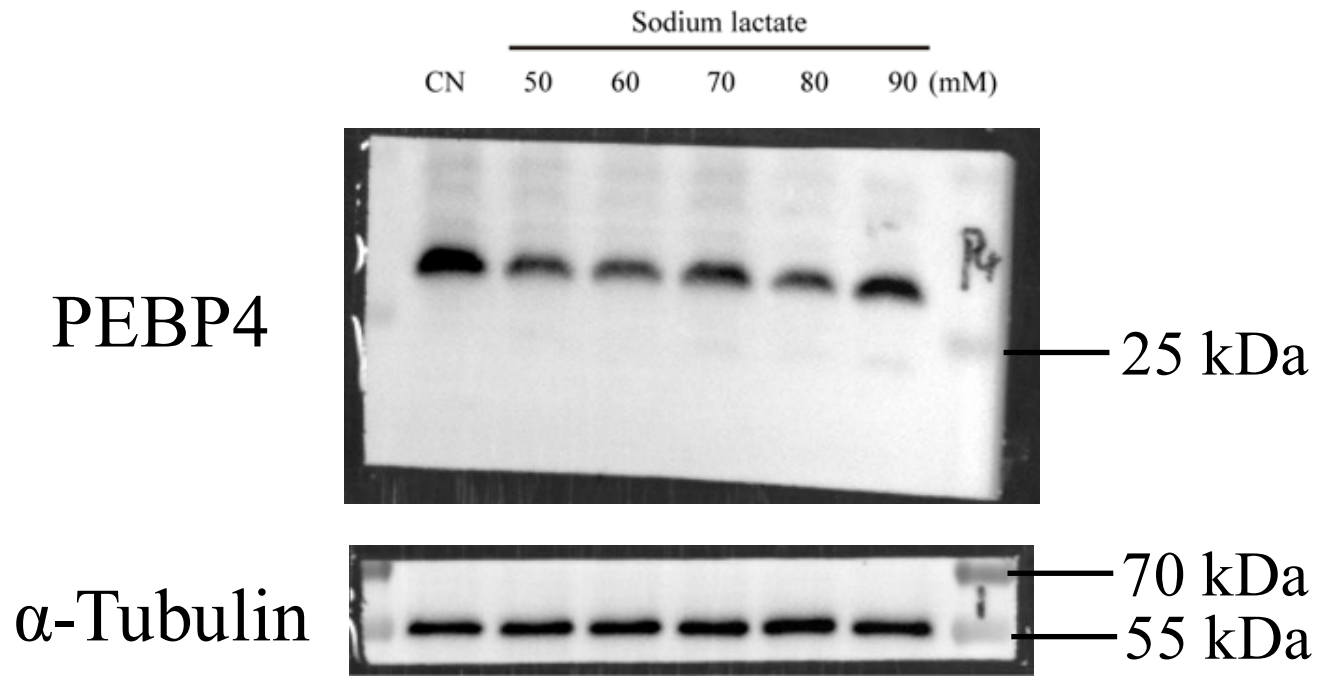

Figure S3

Figure S3D

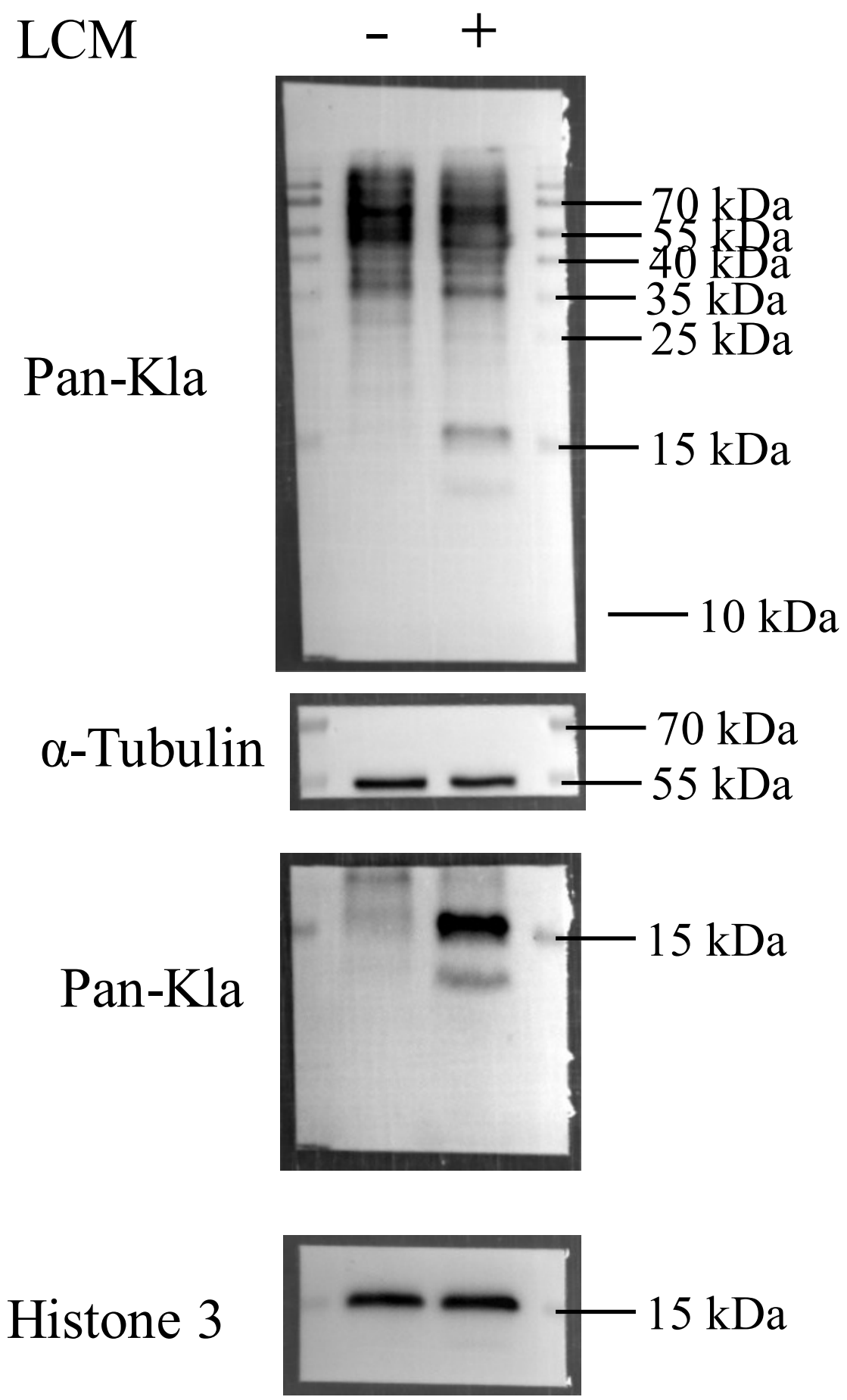

Figure S5

Figure S5H

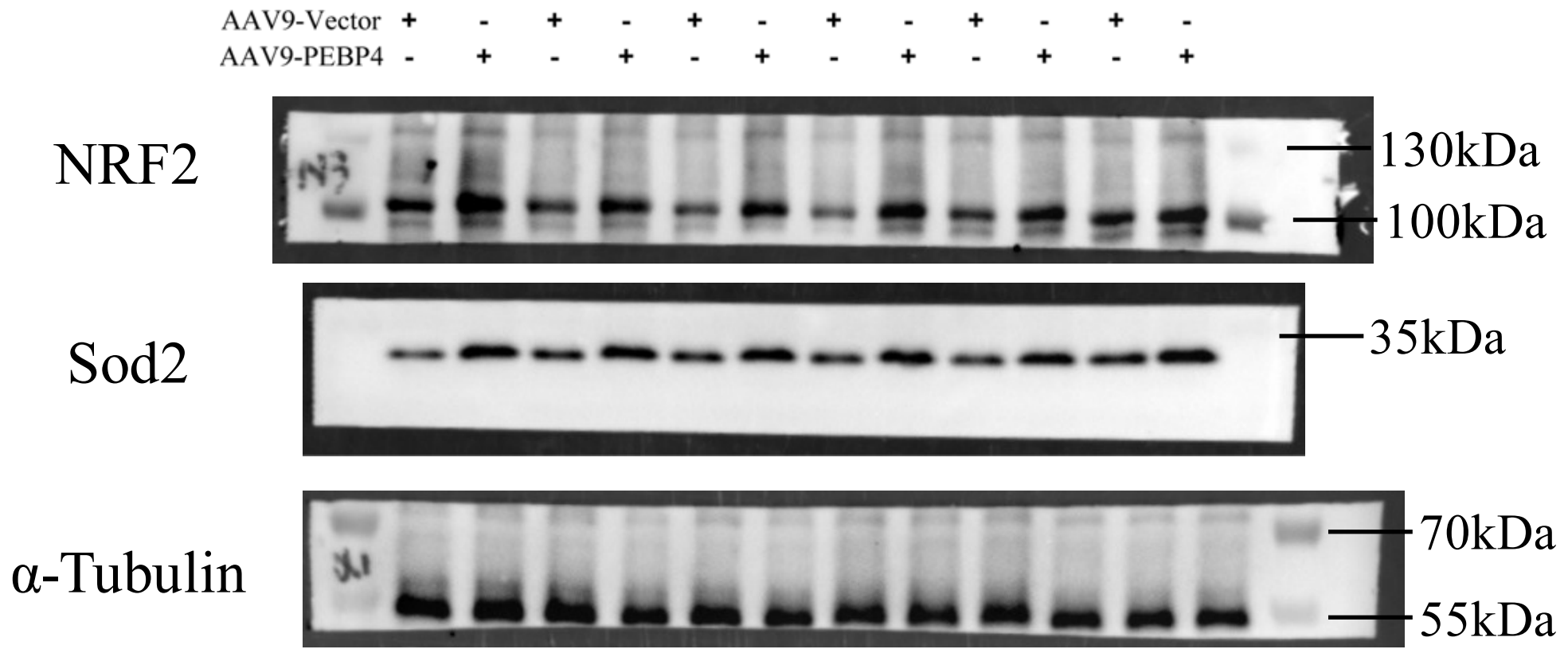

Figure S7

Figure S7C

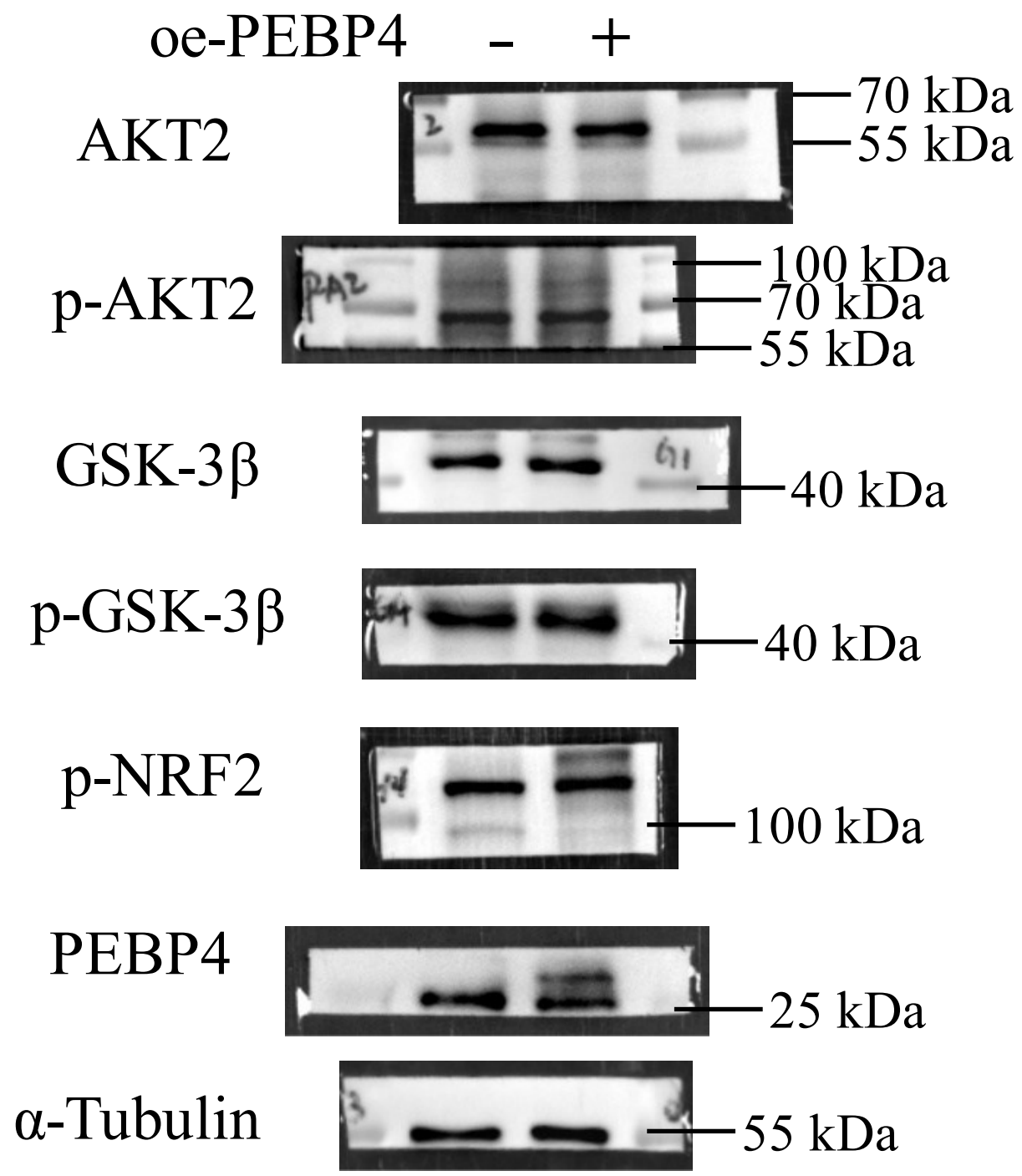

Figure S7

Figure S7D

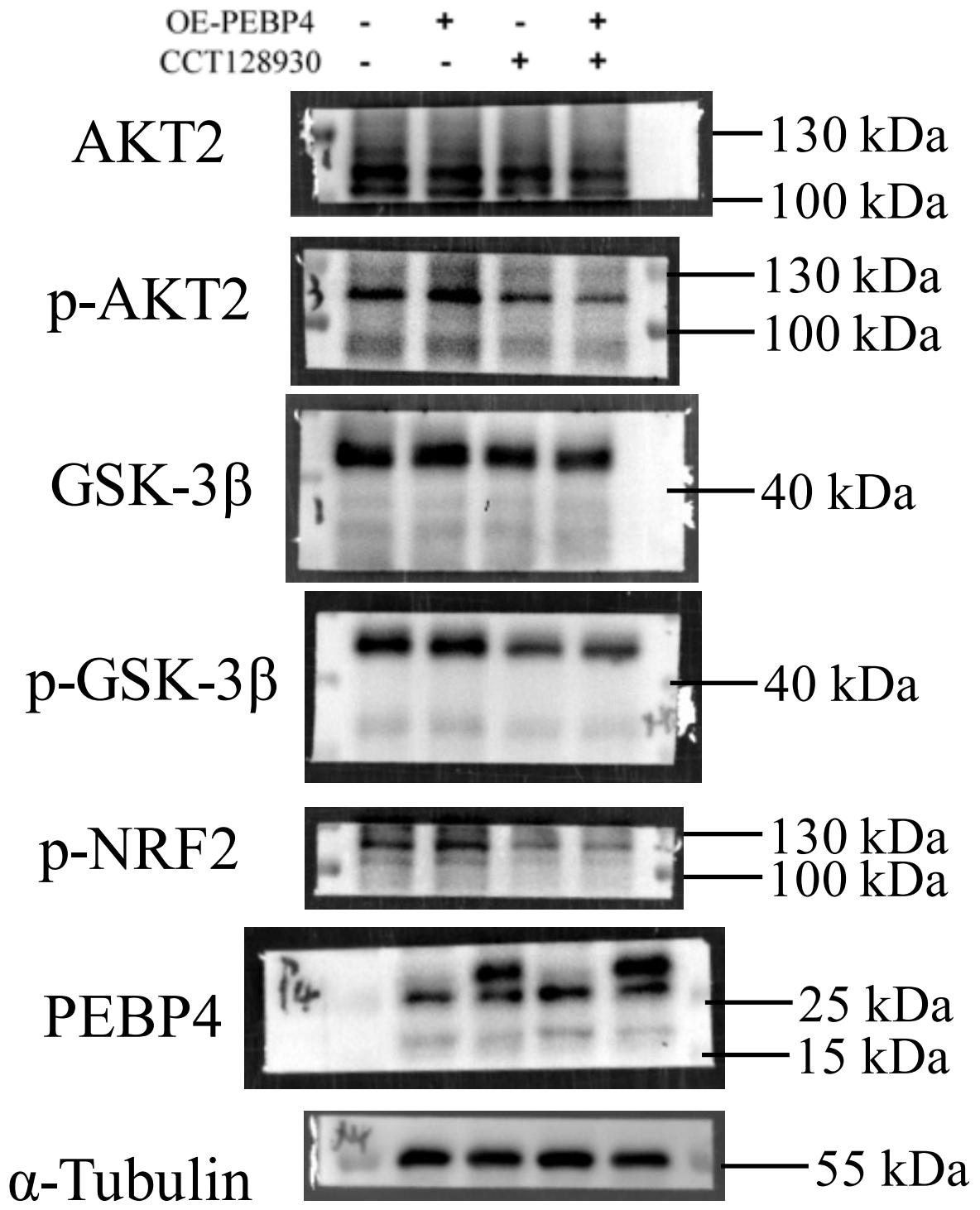

Figure S7

Figure S7E

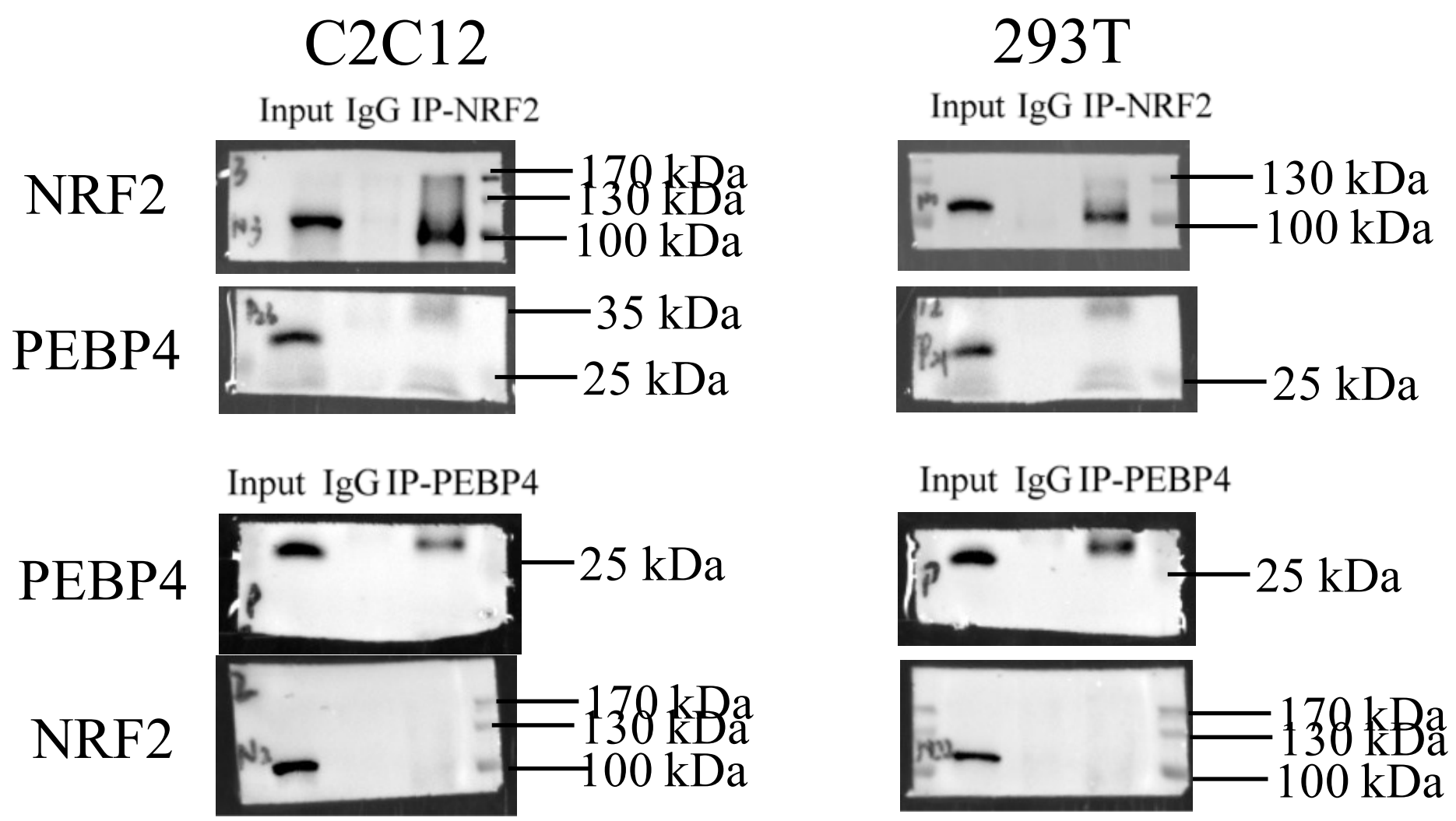

Figure S7

Figure S7F

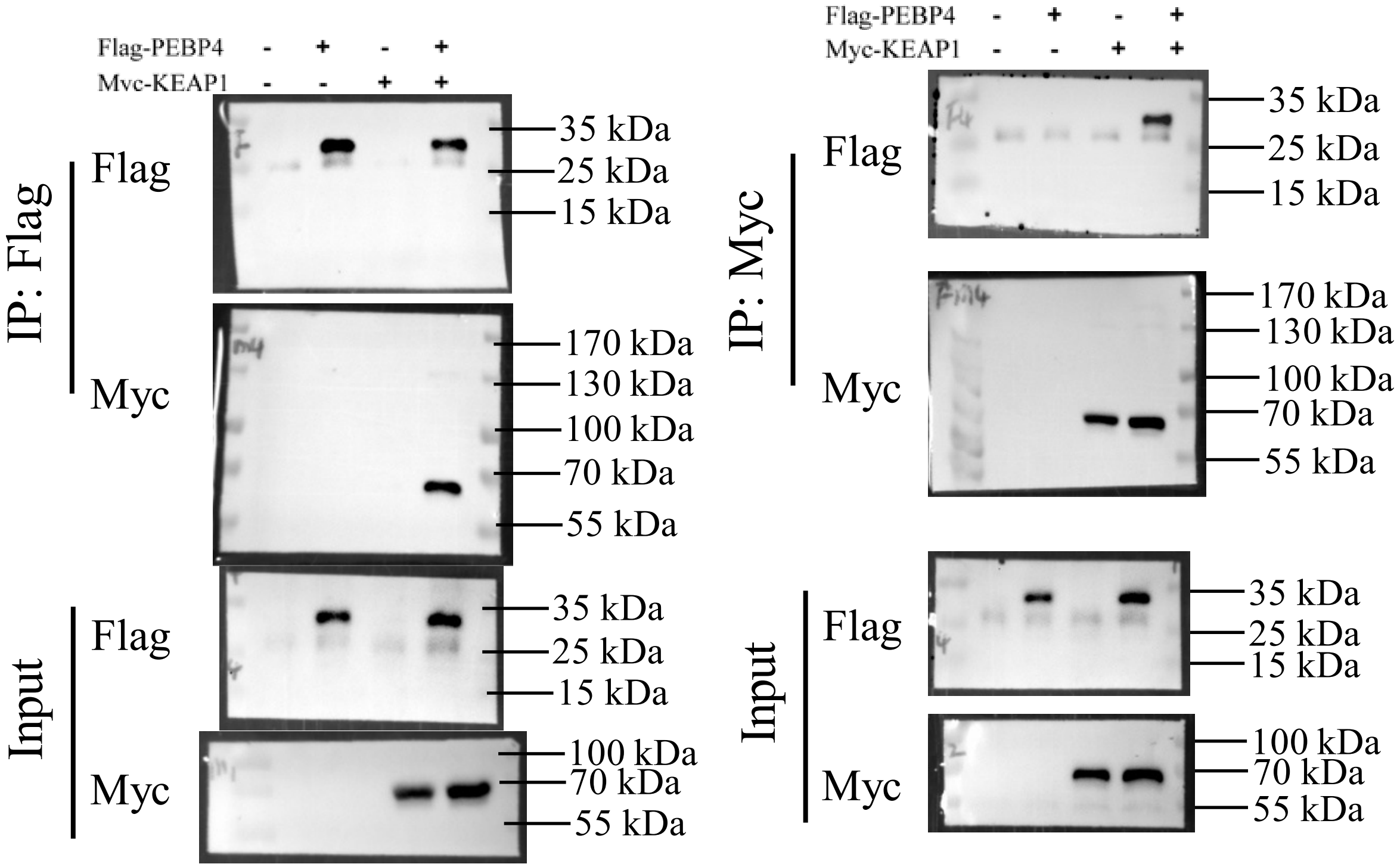

Figure S7

Figure S7G

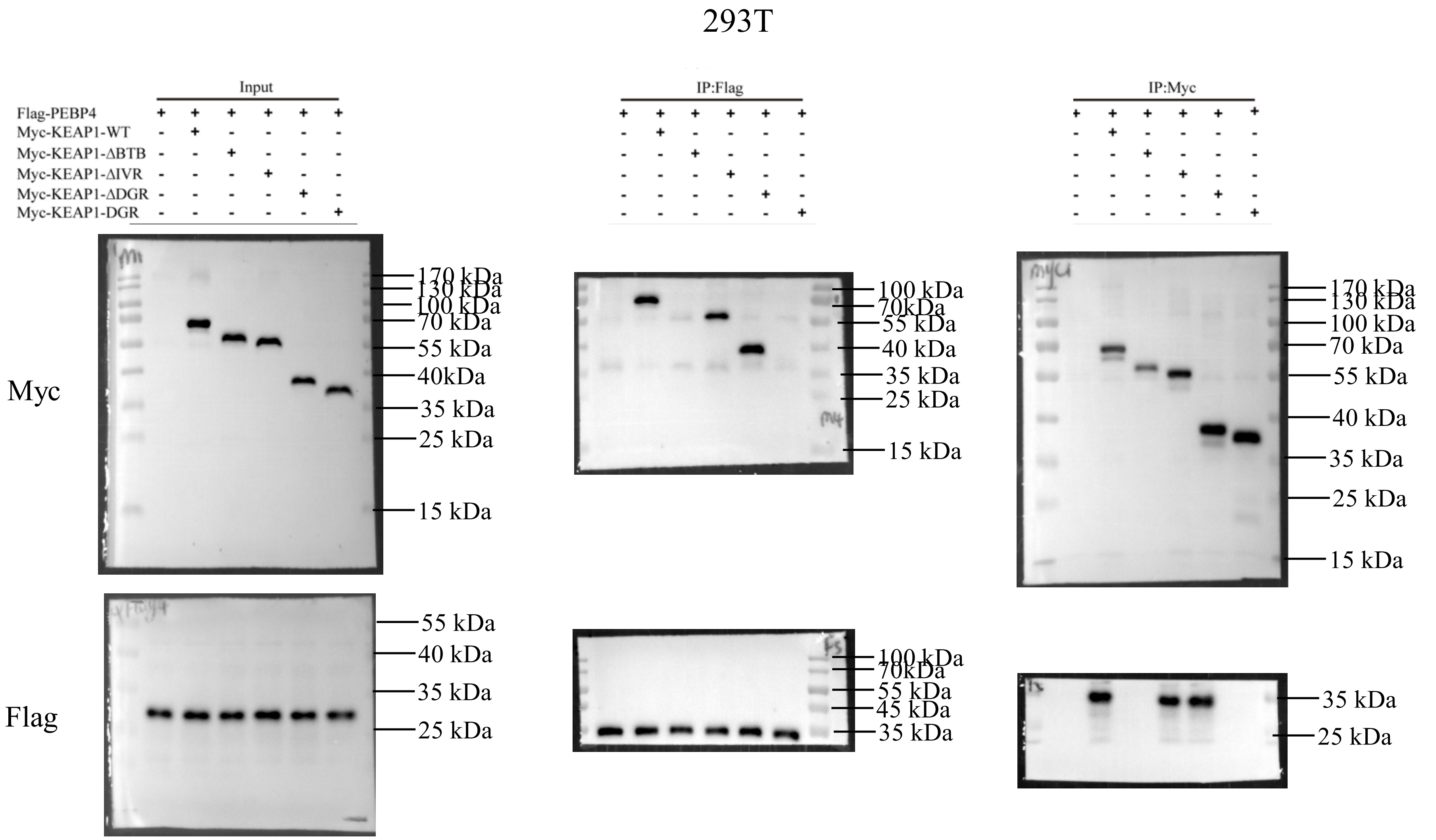

Figure S8

Figure S8B

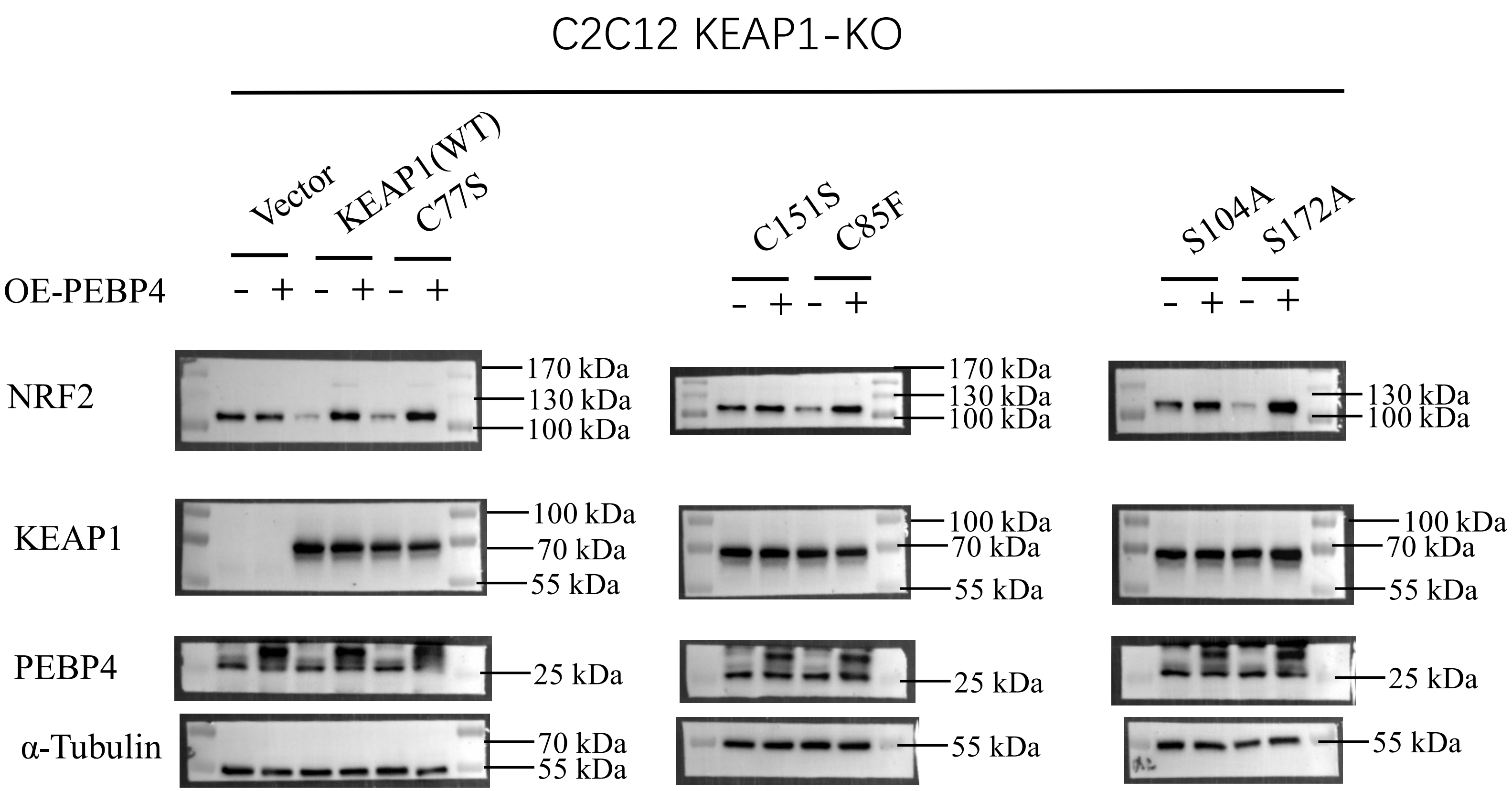

Figure S8

Figure S8C

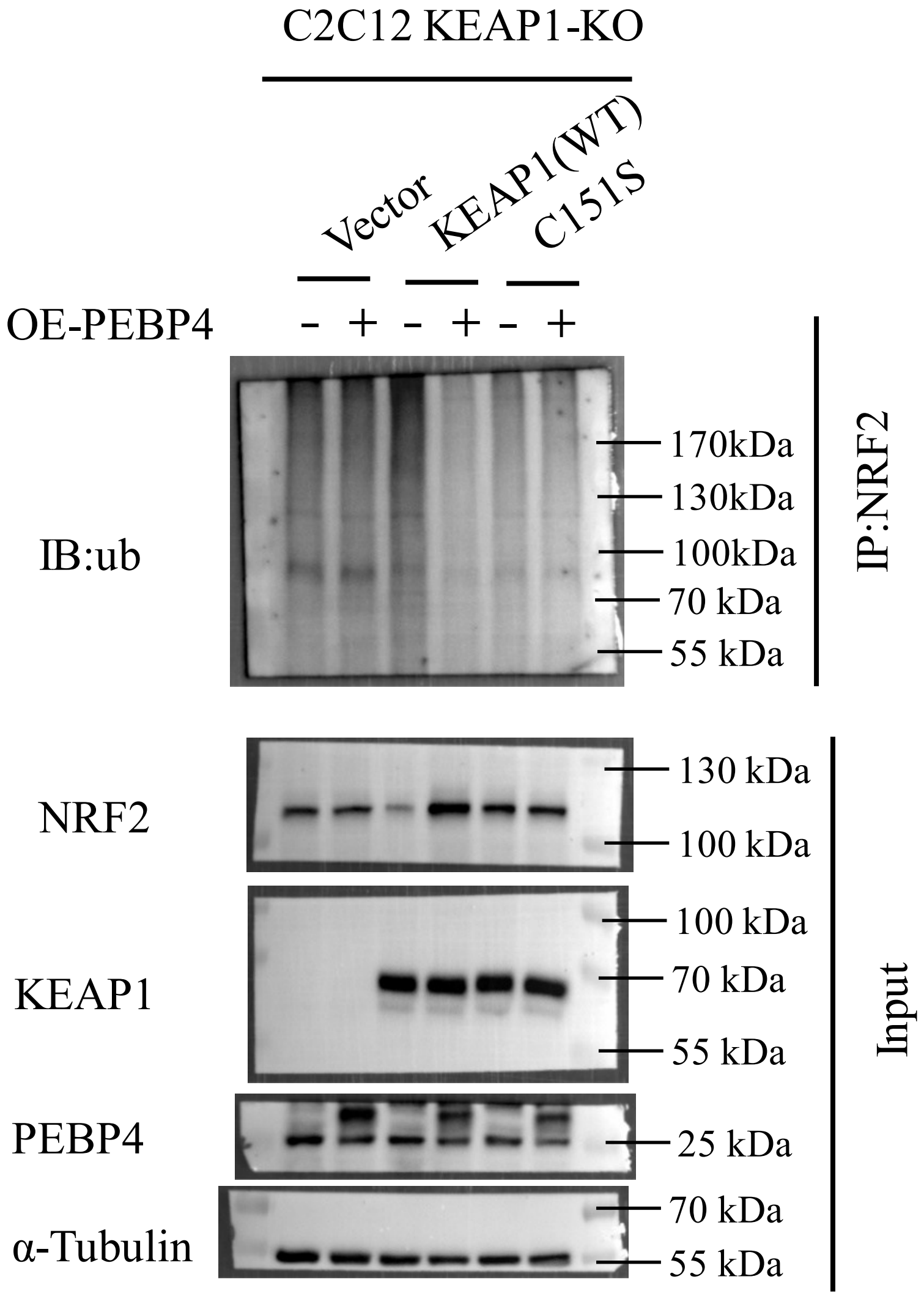

Figure S8

Figure S8D

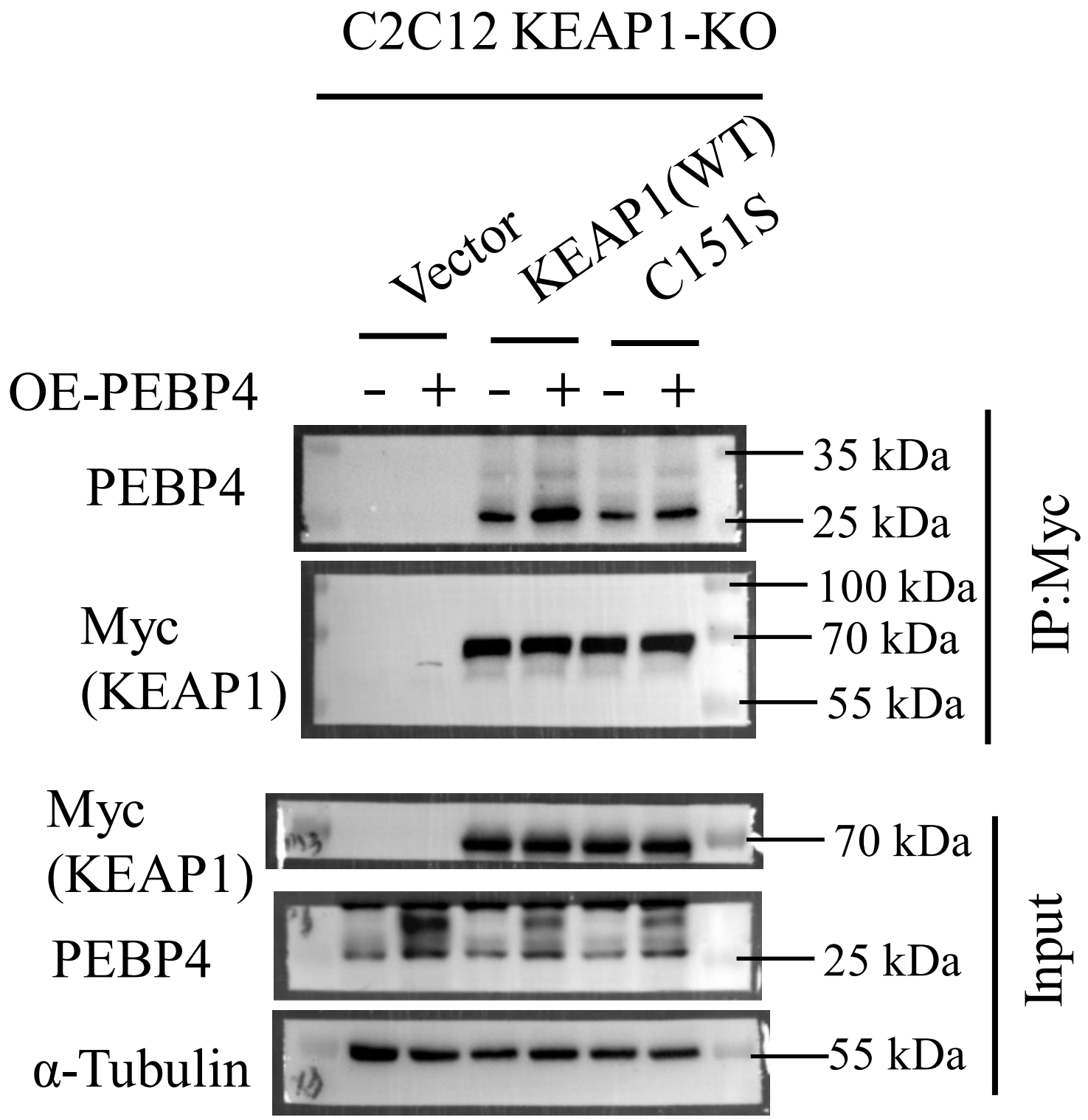

Supplement: Supplementary file 1 [file 41419_2026_8925_MOESM11_ESM.pdf]
