# Supplementary material for: PEBP4 alleviates muscle wasting in lung cancer cachexia via KEAP1–NRF2-mediated redox homeostasis
Source: Cell Death Dis. 2026 Jun 3;17(1):692. doi: 10.1038/s41419-026-08925-5 (PMC13443934; doi:10.1038/s41419-026-08925-5)
Supplement: Supplementary file 2 [file 41419_2026_8925_MOESM10_ESM.docx]

# PEBP4 alleviates muscle wasting in lung cancer cachexia via KEAP1-NRF2-mediated redox homeostasis

Yaru Xia ^1^, Yuqi Han ^2^, Weiquan Li ^2^, Tiexi Yu ^2^, Diaoyi Tan ^2^, Daojia Miao ^2^, Wen Li ^1^, Jiaming Wu ^1^, Qianqian Luo ^1^, Jiemin Li ^1^, Pian Liu ^5^, Hongli Liu ^5^, Guixiao Huang ^3^, Xiaoping Zhang ^2,4^, Hongmei Yang ^1*^

^1^ Department of Pathogenic Biology, School of Basic Medicine, Tongji Medical College, Huazhong University of Science and Technology, Wuhan 430030, China.

^2^ Department of Urology, Union Hospital, Tongji Medical College, Huazhong University of Science and Technology, Wuhan 430022, China.

^3^ Department of Urology, The Third Affiliated Hospital of Shenzhen University: Shenzhen Luohu Hospital Group Luohu People's Hospital, Shenzhen 518000, China.

^4^ Shenzhen Huazhong University of Science and Technology Research Institute, Shenzhen 518000, China.

^5^ Cancer Center, Union Hospital, Tongji Medical College, Huazhong University of Science and Technology, Wuhan 430022, China.

***** Corresponding authors: Hongmei Yang; E-mail address: hyang@hust.edu.cn (H. Yang).

**Competing Interests statement**

The authors declare no competing interests.

**Figure S1. Validation of the LLC-induced cachexia model and identification of candidate genes associated with CAC-induced muscle atrophy.** (A) Time course of body weight changes in PBS and LLC-TB mice (*n* = 6 mice per group). (B) Comparison of tumor-free body weight between PBS and LLC-TB groups (*n* = 6 mice per group). (C, D) Representative images (C) and quantification (D) of epididymal white adipose tissue (eWAT) and inguinal white adipose tissue (iWAT) weight in PBS and LLC-TB mice (*n* = 5 mice per group). (E, F) Representative images (E) and quantification (F) of gastrocnemius (GA) and tibialis anterior (TA) muscles weight in PBS and LLC-TB mice (*n* = 5 mice per group). (G, H) H&E staining of eWAT, iWAT, GA and TA showing cross-sectional morphology in PBS and LLC-TB mice (*n* = 3 mice per group, scale bar = 100 μm). (I) Relative mRNA expression levels of MuRF1, Fbxo32, Saa3, Lcn2, and Scgb1a1 in control and LCM-treated C2C12 myotubes, as determined by qPCR. (J) Representative Western blot images showing SAA3 and SCGB1A1 protein expression in C2C12 myotubes following LCM treatment. (K) qPCR analysis of Scgb1a1, Saa3, and Lcn2 mRNA expression levels in gastrocnemius (GA) muscles of PBS- and LLC-TB–treated mice (*n* = 8 mice per group). (L, M) qPCR analysis demonstrating the efficiency of siRNA-mediated knockdown of Saa3 (L) and Scgb1a1 (M) at the mRNA level in C2C12 myotubes. (O) Representative Western blot analysis confirming siRNA-mediated knockdown of SAA3 and SCGB1A1 at the protein level in C2C12 myotubes. (P, Q) Effects of Saa3 (P) and Scgb1a1 (Q) knockdown on the protein expression of cachexia-associated markers MuRF1 and FBXO32 in LCM-treated C2C12 myotubes. Data are expressed as the mean ± SEM from at least three independent experiments (p < 0.05, **p** < 0.01, ***p*** < 0.001, **p** < 0.0001; ns, not significant).

**Figure S2. Lactate levels in LLC-TB mice tissues, LCM and LCM-treated myotubes.** (A, B) mRNA and protein expression levels of FBXO32 and PEBP4 in C2C12 myotubes treated with LCM, LCM-boiled, or control medium (*n* = 4). (C, D) Lactate concentrations in the serum of LLC-TB mice (C), in LCM compared to control DMEM (D). (E, F) Lactate concentrations in the GA (E) and TA (F) muscles of LLC-TB mice and PBS (*n* = 8 mice per group). (G) Quantification of lactate levels in C2C12 myotubes treated with LCM (*n* = 4). The data are expressed as the mean ± SEM from at least three independent experiments (**p < 0.05*, ***p < 0.01*, ****p < 0.001*; *****p < 0.0001*, ns = not signiﬁcant).

**Figure S3. LLC-derived lactate affects *Pebp4* transcription via H3K18 lactylation in C2C12 myotubes.** (A) Cell viability of C2C12 myotubes following 72 h exposure to increasing concentrations (0–90 mM) of sodium lactate. (B, C) Pebp4 mRNA (B) and PEBP4 protein (C) levels in C2C12 myotubes treated with sodium lactate (5–90 mM). (D) Western blot analysis of pan-histone lactylation in C2C12 myotubes treated with LCM. (E) Lactate concentrations in the supernatant of LLC cells treated with or without the glycolysis inhibitor 2-DG (20 mM) for 48 h. (F) Lactate concentrations in the supernatant of LLC cells treated with or without the LDHA inhibitor GSK-2837808A (10 µM) for 48 h. (G) Genome browser snapshot showing two distinct H3K18la-enriched regions (sites a and b) within the Pebp4 promoter in primary myotubes (GSE195856 [1]), visualized in IGV and aligned to the mm10 (GRCm38) mouse reference genome. (H) ChIP–qPCR analysis of H3K18la occupancy at the *Pebp4* promoter in C2C12 myotubes treated with LCM or exogenous sodium lactate. The data are expressed as the mean ± SEM from at least three independent experiments (**p < 0.05*, ***p < 0.01*, ****p < 0.001*; *****p < 0.0001*, #*p < 0.0001*, ns = not signiﬁcant).

**Figure S4. Statistical analysis of myotube diameter and length, and assessment of PEBP4 overexpression in C2C12 cells and GA muscle tissues.** (A) Scatter plots showing the length and diameter of C2C12 myotubes following LCM treatment, as assessed by MHC immunofluorescence staining. (B) qPCR analysis of *Pebp4* mRNA expression in C2C12 myotubes infected with PEBP4-overexpressing lentivirus. (C) Scatter plots of myotube length and diameter in C2C12 myotubes overexpressing PEBP4. (D) qPCR analysis of *Pebp4* mRNA levels in GA muscles of mice injected with AAV9-PEBP4 or AAV9-vector (*n* = 6 mice per group). The data are expressed as the mean ± SEM from at least three independent experiments (**p < 0.05*, ***p < 0.01*, ****p < 0.001*; *****p < 0.0001*, ns = not signiﬁcant).

**Figure S5. Analysis of NRF2 signaling pathway.** (A) Quantification of PEBP4, NRF2, CAT1, HO-1, and SOD2 protein levels in C2C12 myotubes treated with control medium or LCM. (B, C) qPCR quantification of *Cat1*, *Ho-1*, and *Sod2* mRNA expression levels in GA (B) and TA (C) muscles of PBS and LLC-TB mice (*n* = 6 mice per group). (D) Quantification of *Cat1*, *Ho-1*, and *Sod2* mRNA expression in C2C12 myotubes overexpressing PEBP4. (E) Quantification of NRF2, CAT1, HO-1, and SOD2 protein expression in C2C12 myotubes with PEBP4 overexpression. (F) Quantification of *Pebp4*, *Nox4*, *Duox1* and *Duox2* mRNA expression in C2C12 myotubes with stable PEBP4 overexpression. (G) qPCR analysis of Cat1 and Sod2 mRNA levels in GA muscles following intramuscular administration of AAV9-PEBP4 or control vector (*n* = 6 mice per group). (H) Western blot analysis of NRF2 and SOD2 protein expression in GA muscles from mice injected with AAV9-vector or AAV9-PEBP4 (*n* = 6 mice per group). The data are expressed as the mean ± SEM from at least three independent experiments (**p < 0.05*, ***p < 0.01*, ****p < 0.001*; *****p < 0.0001*, ns = not signiﬁcant).

**Figure S6. Effects of LCM, H_2_O_2_ and ML385 on myotube morphology and protein expression.** (A) Quantification of NRF2, MHC, MuRF1, and FBXO32 protein levels in C2C12 myotubes treated with LCM, with or without PEBP4 overexpression. (B) Scatter plots showing the distribution of myotube length and diameter based on MHC immunofluorescence in LCM-treated or PEBP4-overexpressing myotubes. (C) Cell viability of C2C12 myotubes treated with increasing concentrations of H₂O₂ (0–300 μM) for 48h. (D) Quantification of NRF2, MHC, MuRF1, and FBXO32 protein expression in PEBP4-overexpressing C2C12 cells treated with H₂O₂ in the LCM-induced atrophy model. (E) Distribution of myotube length and diameter following PEBP4 overexpression or H₂O₂ treatment in LCM-treated C2C12 myotubes. (F) Cell viability of C2C12 myotubes treated with increasing concentrations of ML385 (0–100 μM) for 48h. (G) Quantification of NRF2, MHC, MuRF1, and FBXO32 protein levels in PEBP4-overexpressing cells treated with ML385 in the LCM-induced atrophy model. (H) Distribution of myotube length and diameter following PEBP4 overexpression or ML385 treatment in LCM-treated C2C12 myotubes. The data are expressed as the mean ± SEM from at least three independent experiments (**p < 0.05*, ***p < 0.01*, ****p < 0.001*; *****p < 0.0001*, ns = not signiﬁcant).

**Figure S7. Regulatory Interactions Between PEBP4 and KEAP1 or AKT2**. (A) Molecular docking analysis (Z-DOCK and EMBL-EBI tools) predicted binding interactions between PEBP4 and KEAP1 (ΔG = –20.0 kcal/mol), PSMA3 (ΔG = –14.9 kcal/mol), and AKT2 (ΔG = –4.7 kcal/mol), with the strongest affinity observed for KEAP1. (B) Western blot quantification of NRF2, KEAP1 and CUL3 in C2C12 cells overexpressing PEBP4. (C) Western blot analysis of p-AKT2, total AKT2, GSK-3β, p-GSK-3β, and p-NRF2 in PEBP4-overexpressing myotubes. (D) Western blot analysis of AKT2, p-AKT2, GSK-3β, p-GSK-3β, and p-NRF2 in PEBP4-overexpressing C2C12 cells treated with or without the AKT2 inhibitor CCT128930. (E) Endogenous co-immunoprecipitation (co-IP) demonstrating interaction between PEBP4 and NRF2 in C2C12 and HEK293T cells. (F) Co-IP analysis validating exogenous interaction between Flag-tagged PEBP4 and Myc-tagged KEAP1 in HEK293T cells. (G) Co-IP assays using truncated KEAP1 constructs confirming interaction with PEBP4 in HEK293T cells.

**Figure S8. Cys151 within the KEAP1 BTB domain is required for PEBP4-mediated NRF2 stabilization.** (A) Alignment of human and mouse KEAP1 BTB domains showing conservation of Cys77, Cys151, Tyr85, Ser104, and Ser172. (B) Keap1-knockout (Keap1-KO) C2C12 cells stably expressing empty vector or OE-PEBP4 were transfected with pcDNA3 empty vector, wild-type mouse Keap1 (WT), or BTB point mutants (C77S, C151S, Y85F, S104A, S172A). NRF2, KEAP1, and PEBP4 levels were analyzed by Western blot. (C) Keap1-KO C2C12 cells expressing vector or OE-PEBP4 were reconstituted with WT or C151S mutant KEAP1. NRF2 ubiquitination was assessed by immunoprecipitation followed by Western blot. (D) Co-immunoprecipitation assays in Keap1-KO C2C12 cells reconstituted with WT or C151S mutant KEAP1 to evaluate KEAP1 interactions with PEBP4. The data are expressed as the mean ± SEM from at least three independent experiments (**p < 0.05*, ***p < 0.01*, ****p < 0.001*; *****p < 0.0001*, ns = not signiﬁcant).

**Figure S9. Statistical analysis of tumor-free body weight, TA muscle weights, and grip strength in animal experiments.** (A) Tumor-free body weight in LLC-TB mice treated with or without AAV9-PEBP4 (*n* = 8 mice per group). (B) TA muscle weights in LLC-TB mice with or without AAV9-PEBP4 treatment (*n* = 8 mice per group). (C) Grip strength of all limbs across experimental groups (*n* = 8 mice per group). (D) Tumor-free body weight at endpoint in LLC-TB, AAV9-PEBP4, and ML385-treated mice (*n* = 7 mice per group). (E) TA muscle weights in each group (*n* = 7 mice per group). (F) Grip strength of all limbs across treatment groups (*n* = 7 mice per group). The data are expressed as the mean ± SEM from at least three independent experiments (**p < 0.05*, ***p < 0.01*, ****p < 0.001*; *****p < 0.0001*, ns = not signiﬁcant).7的有

**Table S1. Primer sequences.**

| **Name** | **Primer sequences** |
| --- | --- |
| *Fbxo32* | Forward: 5’- TCAGAGAGGCAGATTCGCAA -3’ |
|  | Reverse: 5’- GGGTGACCCCATACTGCTCT -3’ |
| *MuRF1* | Forward: 5’- GTGTGAGGTGCCTACTTGCTC -3’ |
|  | Reverse: 5’- TGAGAGATGATCGTCTGCACT -3’ |
| *Pebp4* | Forward: 5’- ATCAGACTACTCGCCGCCTA -3’ |
|  | Reverse: 5’- GTGCTTGTGTCGGGATCTCT -3’ |
| *Sod2* | Forward: 5’- CAGACCTGCCTTACGACTATGG -3’ |
|  | Reverse: 5’- CTCGGTGGCGTTGAGATTGTT -3’ |
| *Cat1* | Forward: 5’- AGCGACCAGATGAAGCAGTG -3’ |
|  | Reverse: 5’- TCCGCTCTCTGTCAAAGTGTG -3’ |
| *Hmox1* | Forward: 5’- AGGTACACATCCAAGCCGAGA -3’ |
|  | Reverse: 5’- CATCACCAGCTTAAAGCCTTCT -3’ |
| *Nrf2* | Forward: 5’- TAGATGACCATGAGTCGCTTGC -3’  Reverse: 5’- GCCAAACTTGCTCCATGTCC -3’ |
| *Keap1* | Forward: 5’-TCGAAGGCATCCACCCTAAG-3’  Reverse: 5’-CTCGAACCACGCTGTCAATCT -3’ |
| *Saa3* | Forward: 5’-TGCCATCATTCTTTGCATCTTGA-3’ |
|  | Reverse: 5’-CCGTGAACTTCTGAACAGCCT-3’ |
| *Scgb1a* | Forward: 5’-ATGAAGATCGCCATCACAATCAC-3’ |
|  | Reverse: 5’-GGATGCCACATAACCAGACTCT-3’ |
| *Lcn2* | Forward: 5’-GGGAAATATGCACAGGTATCCTC-3’ |
|  | Reverse: 5’-CATGGCGAACTGGTTGTAGTC-3’ |
| *Duox1* | Forward: 5’-AGCCCCTGAAAGAACCCTAC-3’ |
|  | Reverse: 5’- TCCCCATGCGGGATGTAAATG-3’ |
| *Duox2* | Forward: 5’-TCCCGAAGGAAGGTTCAGACA-3’  Reverse: 5’-CAGATTGGGGTTCTCCCGAAT-3’ |
| *Nox3* | Forward: 5’-TGGCAGTAAACGCCTATCTGT-3’ |
|  | Reverse: 5’-CGGAACCCAGAATAACTCGTGTA-3’ |
| *Gapdh* | Forward: 5’- CCACAGTCCATGCCATCACT -3’ |
|  | Reverse: 5’- GACGGACACATTGGGGGTAG -3’ |
| *Pebp4-*  *Promoter a* | Forward: 5’- ACCCAGGGACTTGCTTATCC -3’ |
|  | Reverse: 5’- TTGGAAGGTGGAAGACGCTG -3’ |
| *Pebp4-*  *Promoter b* | Forward: 5’- TAGGGCCTTGGGTATATTAAGCA -3’ |
|  | Reverse: 5’- TATTATAAGGGATTGGGGTGGGGG -3’ |

**Table S2. Antibodies used for western blotting (WB), immunofluorescence (IF) or** **chromatin immunoprecipitation (CHIP).**

| **Primary antibodies** | **Company** | **Cat.n** | **Use** | **Concentration** |
| --- | --- | --- | --- | --- |
| PEBP4 | Abclonal | A17961 | IF  WB | 1:200  1:1000 |
| NRF2 | Abclonal | A21176 | IF  WB | 1:200  1:1000 |
| Phospho-NRF2-S40 Rabbit mAb | Abclonal | AP1133 |  |  |
| Flag-tag | Abclonal | AE005 | WB | 1:1000 |
| Myc-tag | Abclonal | AE010 | WB | 1:1000 |
| LDHA Rabbit mAb | Abclonal | A26493 | WB | 1:1000 |
| AKT2 Rabbit pAb | Abclonal | A18019 | WB | 1:1000 |
| Phospho-AKT2-S474 Rabbit pAb | Abclonal | AP1403 | WB | 1:1000 |
| HA-tag | Abclonal | AE008 | WB | 1:1000 |
| CAT1 | Abclonal | A25422 | WB | 1:1000 |
| HO-1 | Abclonal | A1346 | WB | 1:1000 |
| SOD2 | Abclonal | A19576 | WB | 1:1000 |
| Lamin B1 | Abclonal | A11495 | WB | 1:1000 |
| SAA3 | Abclonal | A11948 | WB | 1:1000 |
| SCGB1A1 | Abclonal | A16997 | WB | 1:1000 |
| Histone H3 Rabbit mAb | Abclonal | A17562 | WB | 1:1000 |
| Cy3-conjugated Goat anti-Mouse IgG (H+L) | Abclonal | AS008 | WB | 1:200 |
| ABflo® 647-conjugated Goat anti-Rabbit IgG (H+L) | Abclonal | AS060 | WB | 1:200 |
| HRP-conjugated Goat anti-Mouse IgG (H+L) | Abclonal | AS003 | WB | 1:2500 |
| HRP-conjugated Goat anti-Rabbit IgG (H+L) | Abclonal | (AS014) | WB | 1:2500 |
| Atrogin-1 | Abcam | Ab168372 | WB | 1:1000 |
| MuRF1 | Proteintech | 55456-1-AP | WB | 1:1000 |
| KEAP1 | Proteintech | 60027-1-Ig | IF  WB | 1:200  1:1000 |
| α-Tubulin | Proteintech | 66031-1-Ig | WB | 1:1000 |
| ubiquitin | Proteintech | 10201-2-AP | WB | 1:1000 |
| Myosin Heavy Chain (MHC) | R&D system | MAB4470 | IF  WB | 1:200  1:1000 |
| Pan Kla | Bioprofile | SHBP0618 | WB | 1:1000 |
| H3K9la | PTM bio | PTM-1419RM | WB | 1:1500 |
| H3K14la | PTM bio | PTM-1414RM | WB | 1:1500 |
| H3K18la | PTM bio | PTM-1427RM | WB  CHIP | 1:1500  6 μg/5x10^6^ cells |
| H2BK16la | PTM bio | PTM-1424RM | WB | 1:1500 |
| Histone H2B | PTM bio | PTM-1006 | WB | 1:1500 |
| H4K5la | PTM bio | PTM-1407RM | WB | 1:1500 |
| H4K8la | PTM bio | PTM-1415 | WB | 1:1500 |
| H4K12la | PTM bio | PTM-1411RM | WB | 1:1500 |
| H4K16la | PTM bio | PTM-1417RM | WB | 1:1500 |
| Histone H4 | PTM bio | PTM-1015RM | WB | 1:1500 |

**Table S3. Clinical characteristics of advanced lung adenocarcinoma with cachexia in this study**

| **Case NO** | **Age** | **Gender** | **Height (cm)** | **Body weight (kg)** | **LDH (U/L)** | **Cancer stage** | **BMI (kg/m^2^)** | **Cachexia (Yes or No)** |
| --- | --- | --- | --- | --- | --- | --- | --- | --- |
| **1** | **46** | **M** | **180** | **54** | **259** | **IIIA** | **16.67** | **Yes** |
| **2** | **54** | **F** | **160** | **35** | **391** | **IV** | **13.67** | **Yes** |
| **3** | **74** | **M** | **170** | **52** | **257** | **IV** | **17.99** | **Yes** |
| **4** | **80** | **M** | **163** | **50** | **352** | **IV** | **18.82** | **Yes** |
| **5** | **55** | **M** | **170** | **52** | **215** | **IV** | **17.99** | **Yes** |
| **6** | **79** | **M** | **171** | **55** | **196** | **IV** | **18.81** | **Yes** |
| **7** | **54** | **M** | **168** | **50** | **277** | **IV** | **17.72** | **Yes** |
| **8** | **53** | **F** | **160** | **35** | **198** | **IV** | **13.67** | **Yes** |
| **9** | **53** | **M** | **160** | **48.5** | **234** | **IV** | **18.95** | **Yes** |
| **10** | **64** | **M** | **169** | **50** | **192** | **IV** | **17.51** | **Yes** |
| **11** | **64** | **F** | **155** | **43.5** | **207** | **IV** | **18.11** | **Yes** |
| **12** | **71** | **M** | **172** | **51** | **245** | **IV** | **17.24** | **Yes** |
| **13** | **61** | **F** | **160** | **47.5** | **220** | **IV** | **18.55** | **Yes** |
| **14** | **60** | **F** | **150** | **40** | **269** | **IV** | **17.78** | **Yes** |
| **15** | **76** | **F** | **157** | **49** | **238** | **IV** | **19.88** | **Yes** |
| **16** | **67** | **F** | **163** | **52** | **205** | **IV** | **19.57** | **Yes** |
| **17** | **48** | **M** | **160** | **47** | **196** | **IV** | **18.36** | **Yes** |
| **18** | **78** | **M** | **174** | **59** | **193** | **IV** | **19.49** | **Yes** |
| **19** | **56** | **F** | **166** | **42.5** | **224** | **IV** | **15.42** | **Yes** |
| **20** | **60** | **F** | **158** | **43** | **236** | **IV** | **17.22** | **Yes** |
| **21** | **58** | **M** | **173** | **57** | **227** | **IV** | **19.05** | **Yes** |
| **22** | **60** | **M** | **175** | **60** | **246** | **IV** | **19.59** | **Yes** |
| **23** | **57** | **M** | **180** | **64** | **193** | **IV** | **19.75** | **Yes** |
| **24** | **69** | **F** | **153** | **42** | **496** | **IV** | **17.94** | **Yes** |
| **25** | **63** | **M** | **158** | **41** | **408** | **IV** | **16.42** | **Yes** |
| **26** | **70** | **M** | **175** | **55** | **184** | **IV** | **17.96** | **Yes** |
| **27** | **30** | **F** | **163** | **53** | **204** | **IV** | **19.95** | **Yes** |
| **28** | **73** | **M** | **172** | **52** | **213** | **IV** | **17.58** | **Yes** |
| **29** | **66** | **M** | **158** | **48** | **284** | **IV** | **19.23** | **Yes** |
| **30** | **68** | **M** | **180** | **61.5** | **305** | **IIIA** | **18.98** | **Yes** |
| **31** | **71** | **F** | **167** | **55** | **180** | **IV** | **19.72** | **Yes** |
| **32** | **64** | **F** | **158** | **48** | **526** | **IV** | **19.23** | **Yes** |
| **33** | **56** | **M** | **178** | **63** | **244** | **IV** | **19.88** | **Yes** |
| **34** | **56** | **M** | **162** | **49** | **284** | **IV** | **18.67** | **Yes** |
| **35** | **73** | **M** | **168** | **53** | **339** | **IV** | **18.78** | **Yes** |
| **36** | **60** | **M** | **172** | **57** | **309** | **IV** | **19.27** | **Yes** |
| **37** | **62** | **F** | **164** | **52** | **269** | **IV** | **19.33** | **Yes** |
| **38** | **61** | **F** | **158** | **48** | **270** | **IIIB** | **19.23** | **Yes** |
| **39** | **84** | **M** | **175** | **55** | **526** | **IV** | **17.96** | **Yes** |
| **40** | **57** | **M** | **186** | **65** | **324** | **IV** | **18.79** | **Yes** |
| **41** | **51** | **F** | **160** | **47** | **339** | **IV** | **18.36** | **Yes** |
| **42** | **55** | **F** | **162** | **50** | **269** | **IIIA** | **19.05** | **Yes** |
| **43** | **65** | **M** | **170** | **57** | **395** | **IV** | **19.72** | **Yes** |
| **44** | **73** | **M** | **168** | **52** | **270** | **IV** | **18.42** | **Yes** |
| **45** | **55** | **F** | **162** | **48** | **302** | **IV** | **18.29** | **Yes** |
| **46** | **70** | **M** | **165** | **51** | **272** | **IV** | **18.73** | **Yes** |
| **47** | **51** | **F** | **165** | **52** | **269** | **IV** | **19.10** | **Yes** |
| **48** | **61** | **M** | **175** | **57** | **260** | **IV** | **18.61** | **Yes** |
| **49** | **26** | **M** | **175** | **57** | **391** | **IV** | **18.61** | **Yes** |
| **50** | **62** | **F** | **157** | **49** | **298** | **IA** | **19.88** | **Yes** |
| **51** | **62** | **M** | **173** | **57** | **267** | **IV** | **19.05** | **Yes** |
| **52** | **51** | **M** | **165** | **50.5** | **317** | **IV** | **18.55** | **Yes** |
| **53** | **67** | **F** | **155** | **48** | **382** | **IV** | **19.98** | **Yes** |
| **54** | **62** | **M** | **172** | **54** | **372** | **IV** | **18.25** | **Yes** |
| **55** | **53** | **M** | **172** | **59** | **329** | **IV** | **19.94** | **Yes** |
| **56** | **42** | **M** | **172** | **57** | **249** | **IV** | **19.27** | **Yes** |
| **57** | **59** | **M** | **171** | **55** | **260** | **IV** | **18.81** | **Yes** |
| **58** | **66** | **M** | **165** | **45** | **624** | **IV** | **16.53** | **Yes** |
| **59** | **62** | **M** | **170** | **50** | **435** | **IV** | **17.30** | **Yes** |
| **60** | **53** | **M** | **170** | **48** | **431** | **IV** | **16.61** | **Yes** |
| **61** | **77** | **M** | **170** | **49** | **608** | **IV** | **16.96** | **Yes** |
| **62** | **62** | **M** | **172** | **54** | **323** | **IV** | **18.25** | **Yes** |

**Table S4. Clinical characteristics of patients with non-cachectic pulmonary nodules in this study**

| **Case NO** | **Age** | **Gender** | **Height (cm)** | **Body weight (kg)** | **LDH (U/L)** | **BMI (kg/m^2^)** | **Cachexia (Yes or No)** |
| --- | --- | --- | --- | --- | --- | --- | --- |
| **1** | **68** | **M** | **168** | **76** | **216** | **26.93** | **No** |
| **2** | **59** | **M** | **170** | **73** | **156** | **25.26** | **No** |
| **3** | **51** | **M** | **165** | **70** | **154** | **25.71** | **No** |
| **4** | **73** | **M** | **173** | **80** | **181** | **26.73** | **No** |
| **5** | **62** | **M** | **172** | **72** | **177** | **24.34** | **No** |
| **6** | **52** | **M** | **173** | **67** | **136** | **22.39** | **No** |
| **7** | **54** | **M** | **165** | **90** | **135** | **33.06** | **No** |
| **8** | **78** | **M** | **168** | **60** | **173** | **21.26** | **No** |
| **9** | **63** | **M** | **160** | **54** | **170** | **21.09** | **No** |
| **10** | **58** | **M** | **168** | **68** | **133** | **24.09** | **No** |
| **11** | **49** | **M** | **170** | **72** | **117** | **24.91** | **No** |
| **12** | **67** | **F** | **163** | **66** | **155** | **24.84** | **No** |
| **13** | **67** | **M** | **176** | **65** | **169** | **20.98** | **No** |
| **14** | **56** | **F** | **153** | **50** | **125** | **21.36** | **No** |
| **15** | **57** | **M** | **170** | **80** | **126** | **27.68** | **No** |
| **16** | **56** | **M** | **170** | **65** | **116** | **22.49** | **No** |
| **17** | **70** | **M** | **175** | **74** | **159** | **24.16** | **No** |
| **18** | **65** | **F** | **160** | **60** | **154** | **23.44** | **No** |
| **19** | **66** | **M** | **169** | **70** | **156** | **24.51** | **No** |
| **20** | **70** | **F** | **160** | **65** | **126** | **25.39** | **No** |
| **21** | **63** | **F** | **163** | **58** | **151** | **21.83** | **No** |
| **22** | **51** | **M** | **177** | **80** | **110** | **25.54** | **No** |
| **23** | **60** | **F** | **157** | **54** | **148** | **21.91** | **No** |
| **24** | **60** | **F** | **160** | **63** | **145** | **24.61** | **No** |
| **25** | **65** | **F** | **160** | **64** | **141** | **25** | **No** |
| **26** | **33** | **F** | **165** | **56** | **129** | **20.57** | **No** |
| **27** | **45** | **M** | **165** | **58** | **156** | **21.3** | **No** |
| **28** | **53** | **F** | **168** | **58** | **111** | **20.56** | **No** |
| **29** | **56** | **F** | **164** | **62.5** | **173** | **23.24** | **No** |
| **30** | **72** | **M** | **180** | **80** | **188** | **24.69** | **No** |
| **31** | **72** | **F** | **155** | **59** | **170** | **24.56** | **No** |
| **32** | **74** | **F** | **156** | **56** | **151** | **23.01** | **No** |
| **33** | **70** | **M** | **170** | **72** | **147** | **24.91** | **No** |
| **34** | **70** | **M** | **168** | **63** | **175** | **22.32** | **No** |
| **35** | **28** | **M** | **185** | **74** | **138** | **21.62** | **No** |
| **36** | **45** | **M** | **171** | **60** | **116** | **20.52** | **No** |
| **37** | **52** | **M** | **168** | **65** | **178** | **23.03** | **No** |
| **38** | **55** | **M** | **180** | **88** | **183** | **27.16** | **No** |
| **39** | **58** | **M** | **170** | **81** | **202** | **28.03** | **No** |
| **40** | **55** | **M** | **167** | **76** | **158** | **27.25** | **No** |
| **41** | **52** | **M** | **170** | **85** | **220** | **29.41** | **No** |
| **42** | **51** | **F** | **160** | **53** | **124** | **20.7** | **No** |
| **43** | **52** | **F** | **156** | **60** | **170** | **24.65** | **No** |
| **44** | **54** | **F** | **157** | **54** | **161** | **21.91** | **No** |
| **45** | **56** | **F** | **164** | **59** | **141** | **21.94** | **No** |
| **46** | **61** | **F** | **150** | **57** | **157** | **25.33** | **No** |
| **47** | **63** | **F** | **168** | **70** | **167** | **24.8** | **No** |
| **48** | **68** | **F** | **158** | **61** | **158** | **24.44** | **No** |
| **49** | **69** | **F** | **158** | **69** | **174** | **27.64** | **No** |
| **50** | **62** | **M** | **160** | **62** | **135** | **24.22** | **No** |
| **51** | **63** | **M** | **170** | **75** | **144** | **25.96** | **No** |
| **52** | **61** | **M** | **182** | **100** | **165** | **30.19** | **No** |
| **53** | **61** | **M** | **176** | **81** | **182** | **26.15** | **No** |
| **54** | **66** | **M** | **170** | **60.5** | **130** | **20.93** | **No** |
| **55** | **61** | **M** | **171** | **67** | **154** | **22.91** | **No** |
| **56** | **68** | **M** | **180** | **75** | **176** | **23.15** | **No** |
| **57** | **62** | **M** | **170** | **64** | **173** | **22.15** | **No** |
| **58** | **71** | **M** | **160** | **54** | **162** | **21.09** | **No** |
| **59** | **68** | **M** | **176** | **70** | **202** | **22.6** | **No** |
| **60** | **65** | **M** | **165** | **65** | **124** | **23.88** | **No** |
| **61** | **69** | **M** | **171** | **65** | **141** | **22.23** | **No** |
| **62** | **65** | **M** | **162** | **66** | **168** | **25.15** | **No** |

Reference：

[1] E. Galle, C.W. Wong, A. Ghosh, T. Desgeorges, K. Melrose, L.C. Hinte, et al., H3K18 lactylation marks tissue-specific active enhancers, *Genome Biol* **23**, 207 (2022).
